# A mutation in mouse Krüppel-like factor 15 alters the gut microbiome and response to obesogenic diet

**DOI:** 10.1371/journal.pone.0222536

**Published:** 2019-09-25

**Authors:** Karen L. Svenson, Lauren L. Long, Steven L. Ciciotte, Mark D. Adams

**Affiliations:** 1 The Jackson Laboratory, Bar Harbor, Maine, United States of America; 2 The Jackson Laboratory, Farmington, Connecticut, United States of America; East Tennessee State University, UNITED STATES

## Abstract

We identified a mouse strain, HLB444, carrying an *N-*ethyl*-N-*nitrosourea (ENU)-induced mutation in a highly conserved C2H2 zinc-finger DNA binding motif of the transcriptional regulator KLF15 that exhibits resistance to diet-induced obesity. Characterization of the HLB444 mutant model on high-fat and chow diets revealed a number of phenotypic differences compared to wild-type controls. When fed a high fat diet, HLB444 had lower body fat, resistance to hepatosteatosis, lower circulating glucose and improved insulin sensitivity compared to C57BL/6J controls. Gut microbial profiles in HLB444 generated from 16S rRNA sequencing of fecal samples differed from controls under both chow and high fat diets. HLB444 shares similar phenotypic traits with engineered full- and adipose-specific *Klf15* knockout strains; however, some phenotypic differences between this mutant and the other models suggest that the *Klf15* mutation in HLB444 is a hypomorphic variant. The HLB444 model will inform further annotation of transcriptional functions of KLF15, especially with respect to the role of the first zinc-finger domain.

## Introduction

Metabolic Syndrome (MetS) is a highly prevalent clinical and public health problem worldwide and is now considered pandemic, affecting an estimated 34% of adults in the US [1, 2]. Clinicians recognize MetS as a complex disorder characterized by coincident presentation of multiple aberrant physiological traits including obesity, hyperglycemia, hypertriglyceridemia, lipidopathies and hypertension. Broad variation in clinical features of MetS among patients presents significant challenges to treating this increasingly prevalent disorder among children and adults. Using the mouse, we aimed to discover new genetic mutations underlying clinical features of MetS using whole-genome ENU mutagenesis in a phenotype-driven approach [3]. One of the strains developed in this effort was initially identified with elevated circulating triglycerides, an important risk factor for cardiometabolic disorders [4, 5] and a high priority target for the development of pharmaceutical interventions [6, 7]. Further phenotypic evaluation of this model revealed that it was resistant to diet-induced obesity, leading us to select this strain for more in-depth functional characterization. Given compelling evidence for an important role of the gut microbiome in obesity [8–11], we also evaluated the microbial profile in the mutant strain and how the community composition differed from controls under standard chow feeding and in response to a high fat diet. Genetic mapping and whole genome sequence analysis confirmed the strain carries a novel missense mutation in the first zinc finger of kidney Krüppel-like factor 15 (kKLF15; KLF15), a known metabolic regulator involved in glucose homeostasis and adipogenesis [12].

Previously developed mouse models with engineered alterations in *Klf15* include a full knockout (*Klf15*^*-/-*^ KO, [13]), an adipose-specific knockout (AK15 KO, [14]) and an adipose-specific transgenic (aP2-KLF15 Tg, [15]), all with the C57BL/6 genetic background. To the extent possible, we compared phenotypes measured in HLB444 to similar measurements reported for these other strains to evaluate functional consequences of the H340Y mutation. This newly identified mutant mouse strain is phenotypically distinct from mice fully deficient in or overexpressing KLF15 and offers a new tool for further elucidating the complex metabolic regulatory activities driven by this important transcription factor.

## Methods

### Mice and husbandry

#### Identification of the ENU mutant

Hypertriglyceride mutant HLB444 was identified from a high-throughput ENU (*N-*ethyl-*N-*nitrosourea) mutagenesis screen that was described previously [3, 16, 17] using inbred strain C57BL/6J (B6), obtained from The Jackson Laboratory (Bar Harbor, ME). A 3-generation breeding scheme generated both dominant and recessive mutations among “G3” progeny. G3 mice were subject to a battery of high-throughput noninvasive phenotyping tests from 6–14 weeks of age, including evaluation of plasma lipids at 8 weeks of age. G3 values were compared to control B6 values to identify phenodeviants. Heritability and mode of inheritance were determined by testing (B6 x HLB444) F1 progeny. The strain was bred to homozygosity prior to genetic mapping by selecting mice with elevated plasma triglycerides for an intercross breeding scheme to establish the mutant strain HLB444 described in this report. This strain is available from The Jackson Laboratory under stock #010952.

#### Animal husbandry

All mice used in this study were housed in a specific pathogen-free facility and maintained on a 12h:12h light:dark cycle beginning at 0600. Mice were housed in pressurized, individually-ventilated Thoren #3 duplex cages (Thoren Caging Systems, Hazelton, PA) with shaved pine bedding (Crobb Box, Ellsworth, ME). Mice had *ad libitum* access to acidified water and standard laboratory chow (LabDiet 5K52) or a purified synthetic high-fat high sucrose diet (HF diet; TD.08811, Teklad diets, ENVIGO, Madison, WI) containing 45% of calories from fat and 41% from carbohydrates (sucrose, maltodextrin, corn starch). All procedures used in this study were approved by the Jackson Laboratory Animal Care and Use Committee and research was conducted in conformity with the Public Health Service *Policy on Humane Care and Use of Laboratory Animals*.

### Phenotyping

#### Experimental cohorts

Phenotype data described in this report were generated from multiple cohorts of HLB444 and control B6 mice. Measurement of baseline phenotypes (under standard chow feeding) in the HLB444 colony was performed on mice aged 8–12 weeks. To investigate whether HLB444 mice harbor additional metabolic phenotypes, we performed a diet study in which mutant and control animals were fed either standard chow or HF diet for seven weeks, when mice were 15–22 weeks of age. During the diet study, body weight was measured weekly and body composition (lean and fat tissue content) was measured at the beginning and end of the study period. Plasma chemistries were measured at the start and end of the diet study and glucose tolerance testing was performed near the end of the study. Organ weights were obtained after necropsy. All measurements were made on 5–8 animals per group.

#### Clinical chemistry assessments

Blood was drawn from the retro-orbital sinus using a heparinized microcapillary tube, after administration of topical anesthetic (tetracaine HCl), and collected into a 1.5 mL Eppendorf tube containing 2μl 10% sodium heparin. Plasma was separated by centrifugation at 10,000 rpm for 10 minutes at 4°C and removed into a clean Eppendorf tube. Before blood collection, food was removed from animals for four hours in the morning, beginning at 0700. Plasma triglycerides were measured using a Beckman Coulter AU680 Clinical Chemistry Analyzer. Measurements were made when mice were eight weeks old; animals with elevated TG values were retested one week later to confirm. Confirmed animals were used for subsequent breeding. In the 7-week diet study, plasma samples were analyzed for TG, total cholesterol, HDL-cholesterol, and glucose. Insulin and leptin were measured using a multiplexed electro-chemiluminescent assay (Meso Scale Discovery, Rockville, MD).

#### Glucose tolerance testing

Glucose tolerance testing was conducted after mice were fasted overnight for 16 hours. The following morning mice were weighed to calculate volume required to administer 2mg glucose/g body weight intraperitoneally. Prior to glucose administration mice were gently restrained to score tail tip with a lancet for blood collection. An initial sample was taken for baseline analysis, followed immediately by bolus administration. Additional samples were obtained at 15, 30, 60, 120 and 180 minutes after glucose injection. Samples were analyzed using the Abbott AlphaTRAK Glucometer with a fresh test strip at each time point.

#### Measurement of body fat

Body fat was measured *in vivo* using the EchoMRI^™^ body composition analyzer (EchoMRI^™^, Houston, TX) on unanesthetized live animals.

#### Histology

Tissues (liver, inguinal fat depot) were harvested for histological analysis after unfasted mice were euthanized by cervical dislocation. Tissues were fixed in Telly’s fixative prior to embedding in paraffin for sectioning and staining with hematoxylin and eosin. Additional liver sections were stained with periodic acid-Schiff (PAS) to evaluate glycogen content.

#### Measurement of liver triglycerides

Total protein was measured using a modified Lowry method (Thermo Scientific catalog #23240) of 3μl of homogenate from 20-40mg of frozen liver sample. This was compared to triglycerides from the same sample. Liver triglyceride values were obtained using a MeOH/chloroform extraction and quantified compared to a standard curve using the Wako Lipid Calibrator (Cat #464–01601), measured at 600nm. Data were normalized to tissue weight and are reported as μg triglycerides/mg of total protein.

#### Fecal sample collection, DNA extraction, library preparation and 16S rRNA sequencing

Fresh fecal pellets were collected for 16S rRNA analysis. Mice were removed from home cages and placed individually in clean cages without bedding and were provided with food and water. Pellets were collected at the same time of day within one hour for each mouse at every time point and stored at -20°C. Samples were collected at the beginning of the study, while mice consumed standard chow (T0). Immediately after this collection, mice were fed HF diet. Fecal samples were collected again after one, two, and three days consuming HF diet (T1, T2, T3, respectively) and at the end of the study, after consuming HF diet for five weeks (TT). Metagenomic DNA from mouse stool pellets was extracted using a modified Qiagen DNEasy protocol. A single stool pellet (10-60mg total weight) was mechanically lysed using Qiagen PowerBead garnet tubes with 1mL of Qiagen InhibitEX buffer. The lysate was then further processed and metagenomic DNA was extracted onboard a QiaCube HT instrument. The 16S rRNA V1-V3 region was amplified using primers 27F (5’-AGAGTTTGATCCTGGCTCAG) and 534R (5’-ATTACCGCGGCTGCTGG) that incorporated Illumina dual indices and sequencing adapters. The amplicons were pooled for sequencing using Illumina 2x300 base V3 reagents on a MiSeq instrument at The Jackson Laboratory for Genomic Medicine Genome Technologies core facility.

### Genetic mapping and identification of candidate gene

To determine the genetic locus containing the mutation leading to elevated TG in HLB444, we performed a backcross breeding scheme using a single affected HLB444 male bred to female C57L/J mice (C57L). C57L mice were obtained from the Jackson Laboratory (Bar Harbor, ME; stock #000668). A preliminary backcross between C57L and non-mutagenized B6 was performed to evaluate whether quantitative trait loci (QTL) for plasma lipids arose due to epistatic interactions between these two strains. No significant QTL were observed in the test cross. For mapping, female progeny of C57L x HLB444 were bred back to their HLB444 male founder to generate N1F1 backcross progeny. All backcross progeny were tested for circulating plasma triglyceride levels as described and genotyped for 130 informative SNPs (i.e., that differ between B6 and C57L) spanning the genome. Evaluation of genotype-phenotype association was performed using R/qtl (https://github.com/rqtl; [18]).

#### Whole genome sequencing

High molecular weight DNA was purified from a tail biopsy of an HLB444 male mouse using a Qiagen DNeasy Blood and Tissue Kit. DNA was fragmented by Covaris E220, and using a Truseq DNA nano library prep generated an average insert size of 300-400bp. The resulting sequencing library was quantified and sequenced on the HiSeq X Ten Illumina platform.

#### Liver RNAseq and analysis

Livers for RNAseq were harvested in the morning from chow-fed nonfasted B6 and HLB444 male mice that had been perfused with DEPC-treated PBS (N = 3 for each genotype). Tissue samples were stored in RNAlater (Thermo-Fisher) per manufacturer's instructions and homogenized in TRIzol (Invitrogen, Carlsbad, CA). Total RNA was isolated by TRIzol® Plus (Thermo-Fisher) according to manufacturer’s methods including on the column DNase digestion. The quality of the isolated RNA was assessed using an Agilent 2100 Bioanalyzer and RNA 6000 Nano LabChip assay. 500ng of total RNA was then reverse transcribed with random decamers and M-MLV RT using the Message Sensor RT Kit (Ambion, Austin, TX). mRNA was then purified from total RNA using biotin-tagged poly-dT oligonucleotides and streptavidin-coated magnetic beads followed by QC using the Agilent Technologies 2100 Bioanalyzer. The mRNA was then fragmented and double stranded cDNA generated by random priming. Illumina-specific adaptors were ligated to the fragments to create libraries, which were then sequenced on an Illumina HiSeq2000.

#### Analysis of whole genome and RNAseq data

Reads were mapped to the mouse reference genome GRCm38 using Burrows-Wheeler Aligner [19]. For RNAseq analysis, normalized counts per gene were determined using DESeq2 [20]. Gene Set Enrichment Analysis (GSEA) [21] was conducted to identify functional groups of liver genes with differential expression. Mouse gene symbols were mapped to human gene symbols using orthology relationships from the Mouse Genome Informatics (MGI) database (www.informatics.jax.org). This approach allowed annotated gene sets from the Molecular Signatures Database [22, 23] to be used for enrichment analysis. GSEA analysis was performed with the HALLMARK gene sets [23] (N = 50 gene sets) and with the Gene Ontology [24] classifications (N = 3713 gene sets with 15–500 component genes).

### 16S data processing, OTU generation, data analysis and statistics

Using Trimmomatic (version 0.32; [25]), primers and adapters were removed and all sequences less than one hundred base pairs were excluded from further processing. Reads were assembled into contiguous amplicon sequences using FLASh (version 1.2.11; [26]). Chimeric sequences were detected and removed using UCHIME (version 4.2.40; [27]). After applying quality control filters as described above, using USEARCH 10 [27], duplicate sequences were collapsed and singletons (minimum size < 5 reads) were removed. Sequences were clustered into 337 OTUs using the cluster_otus command and the original amplicon sequences were mapped to the OTU sequences using the usearch_global command. Using the 2017 release of the Ribosomal Database Project (RDP) classifier version 2.11 training set 16 [28, 29], taxonomy was mapped to the OTUs using a 50% confidence threshold. Taxonomic assignments less than 50% confidence threshold were considered unclassified at the relevant taxonomic resolution.

After OTU generation and taxonomic classification, OTUs that were annotated as unclassified Root or Bacteria were removed from the dataset (total = 2). To further identify spurious OTUs, OTU sequences were aligned with CLUSTALW [30] and a Neighbor-Joining tree was constructed using MEGA [31]. Four OTUs with long branches were determined not to represent 16S rRNA sequences by BLAST search at NCBI and were removed from the dataset. Two samples that had less than 4,000 reads (BM2_1D, 4M3_TD) were removed from the analysis.

Once a final OTU set was generated (331 OTUs), alpha diversity (Shannon and observed) and beta diversity metrics (Bray-Curtis) were performed using Phyloseq (version 1.25.2 [32, 33]) and vegan (version 2.5.2, [34]) packages in R 3.4.1 (R Core Team, 2017). Sequence abundance was normalized to constrain the dynamic range between 0 and 1 and all subsequent analyses were performed on this normalized abundance. A PERMANOVA [35] was performed using the adonis component of the vegan package with 999 permutations for each time point [36] to determine the relative contribution of genotype, sex and their interactions to the overall significance of microbial community alterations. Wilcoxon rank-sum and signed rank-sum tests along with paired and unpaired t-tests corrected for multiple testing comparisons by the Benjamini and Hochberg method (false discovery rate (FDR) < 0.05, [37]) were utilized to assess mean differences between groups. Kruskal-Wallis one-way analysis of variance and mixed design repeated-measures ANOVAs were employed to assess alterations in alpha diversity metrics over time followed by Tukey multiplicity adjustments.

### 16S sequence data availability

All 16S sequence data have been uploaded to the NCBI Sequence Read Archive (SRA) database under BioProject accession number PRJNA505515.

## Results

### Initial characterization of a hypertriglyceridemia mutant

A male G3 presented elevated plasma TG (187 mg/dL) at 8 weeks of age and retesting one week later (204 mg/dL) confirmed the deviant trait. Plasma triglycerides for males of background control strain B6 were 129 ± 12 mg/dL. Breeding of the founder G3 male to B6 females revealed that 0/11 F1 progeny had elevated TG. F1 progeny were then intercrossed and proportionate resultant F2 progeny presented elevated TG, suggesting the trait was heritable and recessive. Plasma TG values differed among sexes in control strain B6 (females: 91±6; males 129±12), therefore sex-specific thresholds were used to identify female and male deviants used to build the HLB444 colony. Threshold values for identification of phenodeviants were set at 110 mg/dL for females and 150 mg/dL for males.

### HLB444 carries a mutation in Klf15

Genetic mapping of increased plasma TG in HLB444 identified a single significant locus on chromosome 6 near 34cM (77Mb), with LOD score of 5.6. Permutation analysis provided the threshold for significance at LOD = 3.44. The 95% confidence interval of the locus was 20cM (44Mb), defining the target candidate gene region as 26–46 cM (53-99Mb).

**Fig 1:**
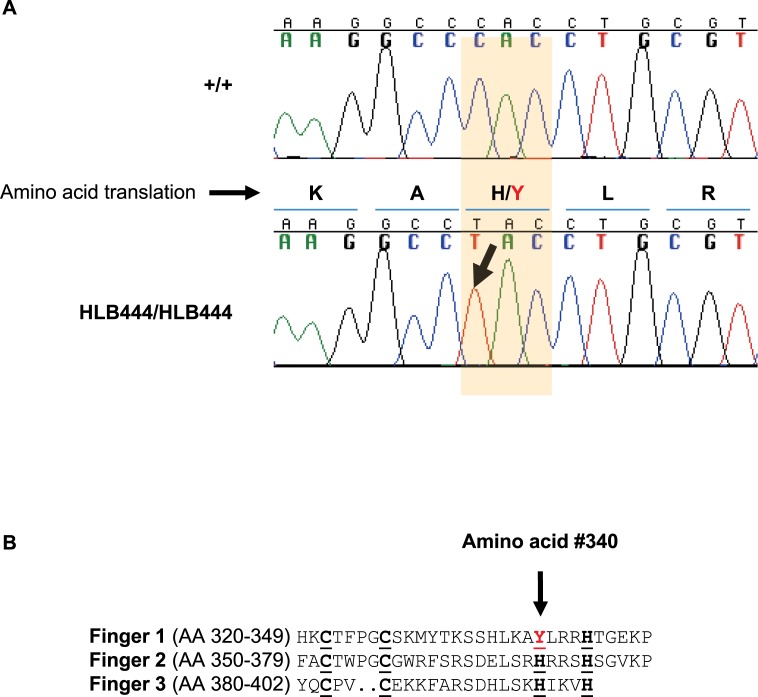
The single base mutation H340Y in KLF15 in HLB444. (A) Chromatograph showing the cytosine to thymine transition mutation, resulting in amino acid change from Histidine to Tyrosine at position 340 in the mouse sequence. B) The H340Y mutation in HLB444 resides within the first of three C2H2 zinc finger motifs that are highly conserved in the KLF family of transcriptional regulators.

Analysis of the chromosome 6 target interval in whole-genome sequence data revealed that HLB444 carries a C to T transition mutation at nucleotide position 1197 in the coding region of Krüppel-like Factor 15 (*Klf15*), resulting in a missense mutation at amino acid 340 in the protein (Fig 1). This mutation, H340Y, changing a histidine residue to tyrosine, occurs in the first of three highly conserved C2H2 zinc-finger binding domains present in KLF15 and all members of the KLF family of transcriptional regulators [38, 39]. No other coding mutations were found in the target interval. Sequencing of PCR amplicons around the mutation site verified that the HLB444 colony was homozygous for the mutation. Therefore, the HLB444 mutant mouse strain represents a new allele of *Klf15*. The strain is publically available as Jackson Laboratory strain stock #010952. Primer sequences used to generate amplicons are in S1 File.

### Metabolic phenotypes of HLB444 differ from Klf15 knockout strain

To determine whether HLB444 exhibited other features of MetS in addition to elevated plasma TG, we determined additional metabolic phenotypes under standard chow feeding and after consumption of a high fat/high sucrose diet.

**Fig 2:**
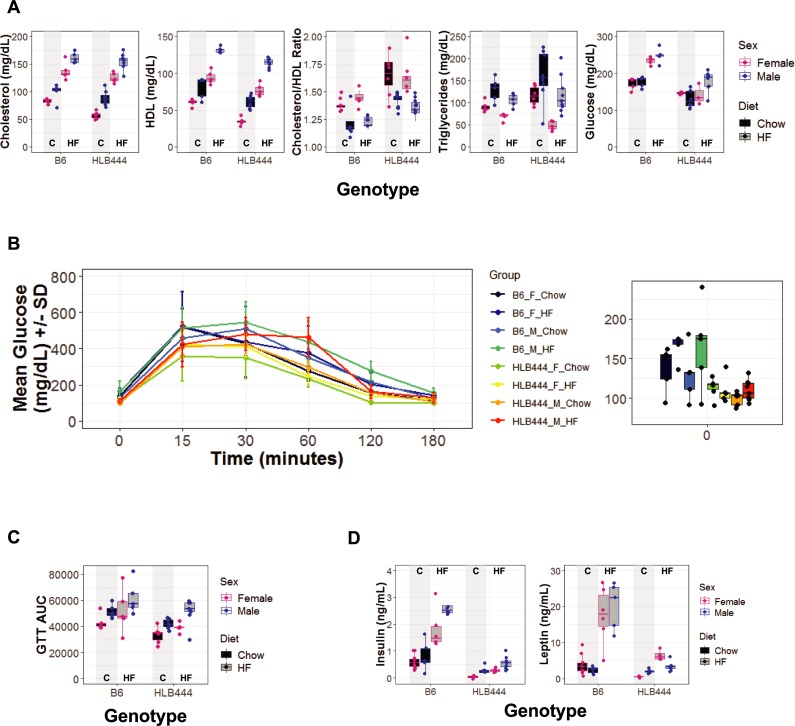
Plasma chemistries and glucose clearance in HLB444 on chow and high fat diets. (A) Plasma chemistries after a 4-hour food removal period (0700–1100). (B) Glucose tolerance testing after overnight fasting; left panel shows mean glucose values in mg/dL ± standard deviation at designated time points after glucose bolus injection at time point 0; right panel shows glucose levels in mg/dL for T = 0, after overnight fasting. (C) Median values for area under the curve for glucose tolerance test presented in panel B, along with the distribution for each experimental group. (D) Non-fasted plasma insulin (left panel) and leptin (right panel) values. In A, C, and D, box and whisker plots show females values in red and male values in blue; chow values are filled with black; high-fat values are filled with gray. Vertical shading in each graph highlights chow values for each strain.

While on the chow diet, HLB444 animals had lower levels of plasma glucose, cholesterol, and HDL than B6 controls (Fig 2A and S2 File). Lower HDL resulted in significantly increased cholesterol:HDL ratios for HLB444 animals. HLB444 animals also had higher triglycerides. On the HF diet, both strains had an increase in cholesterol and HDL and a decrease in plasma triglycerides, as expected [40]. HLB444 animals had a greater decrease in triglyceride levels in response to the HF diet than did control animals. Only B6 controls exhibited an increase in plasma glucose on the HF diet, but did not reach diabetic levels [41].

Overnight fasted glucose levels in HLB444 fed HF diet were significantly lower than controls, but the animals were not overtly hypoglycemic (Fig 2B, left, 0 minutes), in contrast to the *Klf15*^*-/-*^ KO in which glucose levels fell to 45 and 58 mg/dL after an overnight fast for females and males, respectively [12]. The *Klf15*^*-/-*^ KO demonstrated significantly improved glucose clearance compared to controls, explained by increased insulin secretion during the glucose tolerance test [12]. In contrast, glucose clearance was not significantly different between HLB444 and controls on either chow or HF diet (Fig 2B and 2C).

Circulating insulin and leptin were measured from non-fasted animals at the end of the diet study. Insulin levels were similar in HLB444 and B6 on the chow diet, and increased to a much larger extent on HF diet in controls than in HLB444 animals (Fig 2D). Baseline insulin was also similar to controls in the *Klf15*^*-/-*^ and the adipose-specific KO strains. In the transgenic strain with overexpression of KLF15 in adipose (aP2-KLF15 Tg), insulin was significantly higher in animals on HF diet compared to controls [15]. Leptin levels did not differ between strains at baseline and did not change with HF diet in HLB444, but increased in B6 animals on HF diet (Fig 2D). By comparison, leptin levels decreased significantly in aP2-KLF15 Tg animals on HF diet [15]. Plasma chemistry values for B6 were consistent with previous similar studies represented in the Mouse Phenome Database (MPD; http://phenome.jax.org).

### HLB444 is resistant to fat gain and hepatic steatosis with high fat feeding

**Fig 3:**
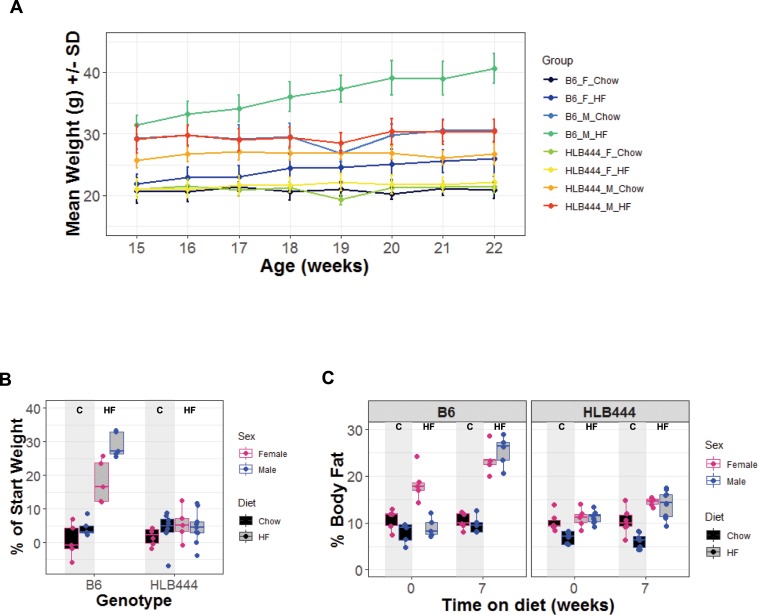
Weight gain and body fat accumulation in HLB444 on chow and high fat diets. (A) Weight curve over a 7-week study of control and HLB444 strains fed chow or high-fat diet, beginning when animals were 15 weeks old. (B) Weight gain in control and HLB444 as percent of start weight in 7-week study. (C) Body fat content measured by EchoMRI in control and HLB444 at start and end of 7-week study. In B and C, box and whisker plots show females values in red and male values in blue; chow values are filled with black; high-fat values are filled with gray. Vertical shading in each graph highlights chow values for each strain.

Body weight and composition were remarkably well maintained in in HLB444 after seven weeks of HF feeding (Fig 3). Over the diet study period total weight gain after HF feeding, expressed as percent of start weight, was minimal for both sexes of HLB444 animals, whereas B6 animals added significantly to start weights (Fig 3B and 3C). On chow, percent body fat did not differ between the strains. Weight gain after HF diet was accompanied by increased body fat in controls, whereas body fat in HLB444 animals did not change with HF diet. We investigated whether the attenuated weight gain on HF diet in HLB444 was due to lower food consumption using a metabolic cage monitoring system and found no differences from B6 on chow or HF diet in any metabolic parameters measured (food and water consumption, respiratory exchange ratio, diurnal activity; S1 Fig). Metabolic parameters were also unchanged during metabolic cage testing reported for AK15 KO and aP2-KLF15 Tg animals [14, 15].

**Fig 4:**
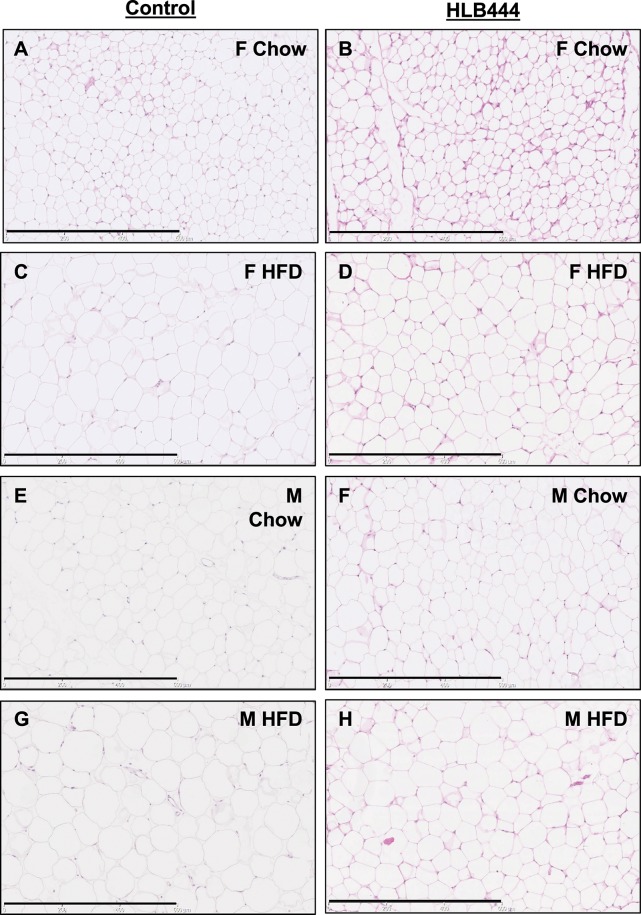
Adipocyte size on chow and high diets. Histologic sections of inguinal adipose tissue from age-matched mice fed chow (A, B, E, F) or HF diet (C, D, G, H); F, female; M, male. Sections were stained with hematoxylin and eosin. Images are shown at 20x magnification. Black bar is 600μm.

From histological sections, we observed that adipocytes appear smaller in HLB444 animals compared to controls on both diets (Fig 4), consistent with adipocyte assessments in the AK15 KO (chow diet). Interestingly, this was also observed in the aP2-KLF15 transgenic fed HF diet [15]. Smaller adipocyte size has been implicated in improved insulin sensitivity in animal models and in lean and obese humans [14, 42, 43].

**Fig 5:**
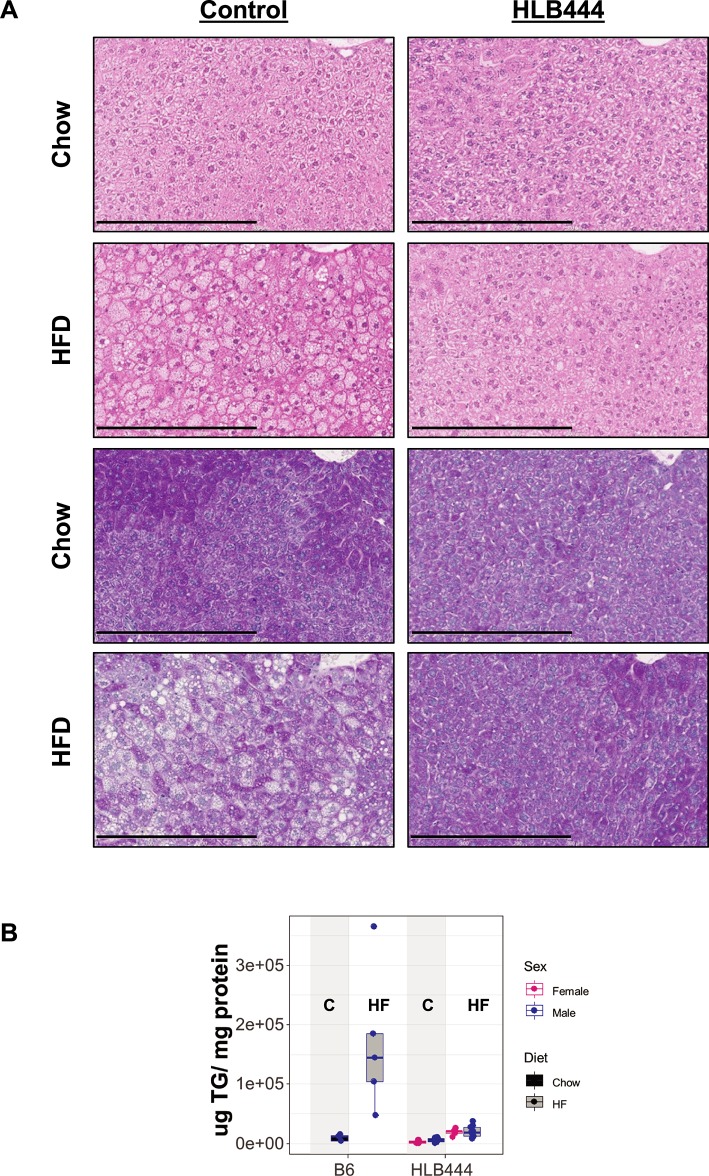
Liver histology on chow and high fat diets. (A) Histologic sections of livers from control (left panels) and HLB444 (right panels) male mice fed chow or HF diet. Sections were stained with hematoxylin and eosin. Lower sections were also stained with PAS to evaluate glycogen content. Images are shown at 20x magnification. Black bar is 300μm. Mice were not fasted prior to tissue harvest. (B) Liver triglyceride content in control and HLB444 on chow and HF diet. Box and whisker plots show females values in red and male values in blue; chow values are filled with black; high-fat values are filled with gray. Vertical shading highlights chow values for each strain.

Qualitative assessment of hepatic response to HF diet by histological evaluation revealed that HLB444 is resistant to HF-induced hepatosteatosis (Fig 5A) while maintaining normal glycogen storage. B6 accumulated hepatic lipid depots with HF feeding, whereas HLB444 did not. Liver sections from males are shown in Fig 5, but similar results were seen in female HLB444. Further, hepatic triglyceride content did not differ between B6 and HLB444 at baseline (Fig 5B). The HF diet did not significantly change hepatic triglyceride content in HLB444, but produced an increase of up to 16-fold from baseline in controls. Lower liver triglyceride levels in HLB444 than control animals are consistent with histological observations of lower fat accumulation in HLB444 livers on HF diet. By contrast, hepatic triglycerides on HF diet were nearly two-fold greater than controls in the aP2-KLF15 transgenic mouse strain [15].

### Metabolic and ER stress pathways are altered in HLB444 liver

Transcriptional profiling was performed on livers from chow-fed males of control and HLB444 strains. We identified 3,286 genes as differentially expressed, out of 12,086 with measurable expression levels, using a false discovery rate threshold of <0.01. Analysis of “hallmark” gene expression using GSEA [21, 23] found that HLB444 livers had decreased expression of genes related to fatty acid, bile acid, and xenobiotic metabolism, adipogenesis, and interferon-g and interferon-α responses compared to B6, and had increased expression of genes related to the unfolded protein response (UPR; S1.1–S1.4 Table). Similar results were obtained when considering gene sets based on Gene Ontology (GO) terms, in which HLB444 livers showed significantly lower expression of genes related to amino acid and fatty acid catabolism. Reduced amino acid catabolism was associated with reduced gluconeogenesis in the KLF15 knockout mouse [12] and may contribute to lower steady-state glucose values maintained in HLB444 on chow and HF diet. Endoplasmic reticulum (ER) stress due to misfolded protein accumulation initiates the unfolded protein response [44] and is initiated by metabolic disorders including obesity, liver disease and type 2 diabetes through interactions with inflammatory and stress signal pathways and inadequate management of lipotoxicity in the liver [45, 46]. It may be the case that an activated baseline UPR in HLB444 allows better response to high fat feeding, resulting in resistance to HF-induced obesity. In intestinal epithelial cells, the UPR plays an important role in innate immune function [47, 48]. Jung, et al. demonstrated that mice lacking KLF15 exhibited increased hepatic ER stress, inflammation, and JNK activation compared to control mice, but did not show hepatic insulin resistance or fatty liver under high-fat feeding conditions, and hypothesized that these effects were mediated by decreased mTORC1 activity [49]. Expression of the *Mtor* gene in HLB444 liver did not differ from B6, however expression was reduced of certain mTORC1-regulated genes involved in cellular energy homeostatis, including *Pparg* (5-fold lower in HLB444) and *Rev-Erba* (2-fold lower). The Jung study identified KLF15 as an important molecular link between ER stress and insulin action.

Expression of *Klf15* has been demonstrated to be under control of the peripheral circadian clock in heart [50] and adipocyte progenitors [51], mediated by binding of PER3 and BMAL1/ARNTL at the *Klf15* promoter. In HLB444 liver, expression of *Per3* was strongly reduced (down 40-fold) and expression of *Arntl* was strongly increased (25-fold); both genes were in the top 15 most significantly differentially expressed. Thus, rather than being purely downstream of the clock signal, our results suggest that Klf15 can alter the expression of clock regulators in liver. This bears further investigation.

Six other members of the Klf Family (1, 9, 10, 11, 13, 16) were downregulated in HLB444 liver, suggesting a common coregulatory mechanism among them. Of these, KLF11 has been implicated in metabolic processes such as regulation of pancreatic beta cell function [52], and regulation of cholesterol-mediated gene expression [53]. As some KLFs are known to interact and even compete [54], this result suggests KLF15 may participate in as yet unrealized interactions with these family members.

To investigate tissue expression of *Klf15* in HLB444 we performed RT-PCR on a selection of tissues known to express *Klf15* (brown and white adipose, liver, skeletal muscle, heart, lung; http://biogps.org) in mutant and control males fed chow. Because KLF15 regulates PPARg in adipogenesis [44, 55] we also investigated expression of *Pparg* in these tissues. Expression of both *Klf15* and *Pparg* was detectable in each tissue from each strain. Expression of *Klf15* in HLB444 was increased in heart and lung, and expression of *Pparg* in HLB444 was increased in heart and reduced in liver (S2 Table). Primer sequences used for RT-PCR are in S1 File.

### Gut microbial profiles of HLB444 differ from controls on chow and high fat diet

Because of the important role of the gut microbiome in metabolic health [56–61], we investigated whether the microbial profile of HLB444 differs from B6 under either chow or HF diet. Fecal pellets from B6 and HLB444 animals were collected at baseline on chow diet (T0). Animals were then switched to HF diet and stool pellets were collected after 1, 2, and 3 days, and after five weeks on HF diet. 16S rRNA profiling was used to characterize changes in the microbial community between the two mouse strains and in response to HF diet.

**Fig 6:**
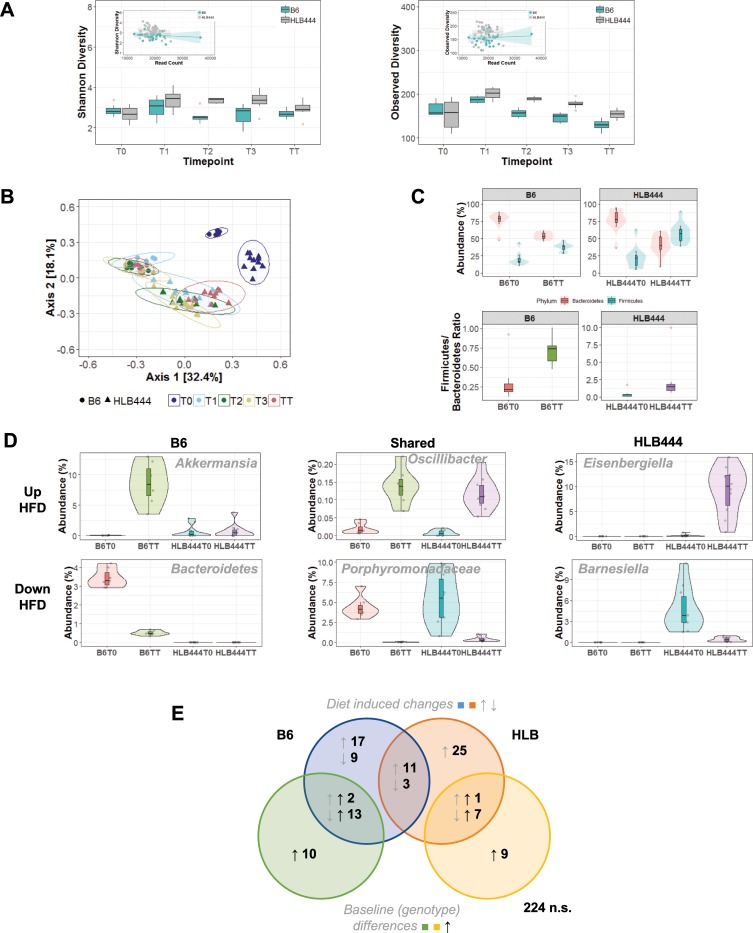
Microbial community composition on chow and high fat diets based on 16S analysis. **(**A) Box and whisker plots of Shannon diversity (left) and observed diversity (right) as a function of HF diet transition for B6 (teal) and HLB444 (gray). Red dots represent outliers. Insets on each graph represent the correlation between read depth and the relevant metrics for B6 and HLB444 where each dot is a sample. Shaded areas represent the 95% confidence interval. (B) PCoA plot using Bray-Curtis dissimilarity matrix for B6 (circles) and HLB444 (triangles) as a function of transition to HF diet (T0-TT, colors). Ellipses represent the 95% confidence intervals. (C) Top: Violin plots of abundance of Bacteriodetes (red) and Firmicutes (teal) on chow (T0) and HF diet (TT) for B6 (left) and HLB444 (right). Bottom: Firmicutes-to-Bacteriodetes ratio on chow (T0) and HF diet (TT) for B6 (left) and HLB444 (right). (D) Violin plots demonstrating the differential abundance patterns of representative OTUs. (E) Euler diagram summarizing the significant trends observed in communities between strains at baseline (chow; green/yellow circles) and on HF diet (blue/orange circles). Gray arrows represent OTUs that went either up or down when after HF diet; black arrows represent OTUs that were higher at baseline.

B6 and HLB444 animals exhibit similar alpha diversity on chow diet (Fig 6A). When transitioned to HF diet, there were transient differences in Shannon diversity between B6 and HLB444 animals that stabilized by five weeks. At baseline, the number of observed OTUs was not significantly different between B6 and HLB444 animals (Fig 6B). When HF diet was introduced, there were immediate increases in the number of observed OTUs for B6 and HLB444 animals. Over sustained exposure to HF diet, HLB444 animals continued to maintain higher microbial diversity than B6 animals (Fig 6A; see S3 Table for descriptive statistics; see S4.1 and S4.2 Table for ANOVA statistics). Since the power to detect rare organisms increases as sampling depth increases [62], we examined the relationship between read depth and alpha diversity metrics. Shannon and observed diversity metrics were insensitive to read depth for both genotypes and thus rarefaction was not performed (Fig 6A, insets; see S4.3 and S4.4 Tables for read depth statistics).

To evaluate the differences in community composition between B6 and HLB444 animals on chow and HF diet, we performed principle coordinates analysis (PCoA) on the Bray-Curtis dissimilarity matrix. PCoA revealed four distinct clusters where the first axis separated the data largely by diet and the second mostly by genotype (Fig 6B). Notably, HLB444 and B6 animals exhibited robust differences in community composition at baseline that changed immediately following exposure to HF diet. Immediate and sustained exposure to HF diet revealed distinct community shifts by genotype. To statistically support PCoA visualization, PERMANOVAs (call: Adonis, vegan) were performed at each time point and the model incorporated both genotype and sex (S4.5 Table). There were strong differences in community composition by genotype at each time point. For instance, *Akkermansia muciniphila* was greatly increased in B6 but not HLB444 on HF diet (Fig 6D). This result is in contrast to findings from previous studies showing a role for *A*. *muciniphila* in affecting attenuation of an obese phenotype [57, 63]. Because sex and the interaction between genotype and sex was not significant, data from males and females were pooled for all subsequent analyses (S4.5 Table). There was significant similarity in the community structure among all HF diet time points as demonstrated by extensive overlap of 95% confidence intervals (Fig 6B). We therefore chose to focus on the T0 (chow) and TT (after 5 weeks HF diet) datasets in subsequent analyses to assess differences in long-term response to HF diet in each mouse strain.

In mice and in humans, a shift from Bacteriodetes to Firmicutes has been observed with high fat feeding and obesity [56, 64]. The ratio of Firmicutes to Bacteriodetes increased with HF diet in both genotypes, as expected, but changed far less in the mutant strain than in controls (Fig 6C). The attenuated shift in HLB444 is consistent with resistance to diet-induced obesity.

Mouse stool microbial profiles clustered separately by genotype and diet because differential patterns of shared OTU relative abundance were present across the community landscape. Of the entire community (331 OTUs), 114 OTUs demonstrated significant differences in comparisons between mouse strains and diets, examples of which are shown in Fig 6D. The differential abundance of OTUs with respect to genotype and diet response are depicted by the Euler diagram in Fig 6E. Many OTUs were differentially abundant as a function of genotype only; others diet only. Only 14 OTUs exhibited similar changes in relative abundance (11 increased; 3 decreased) for both genotypes following transition to HF diet. In contrast, there were 25 OTUs that were differentially abundant only in HLB444 after shift to HF diet (all increased abundance), while in B6, 26 OTUs were uniquely altered in relative abundance on HF diet, suggesting substantially different microbial responses to HF diet in each strain. None of these groups of differentially abundant OTUs followed robust taxonomic classification profiles. See S2 Fig for violin plots of all OTUs, S3 File for taxonomic classification for all comparisons in Euler diagram and S4 File for all statistical comparisons.

#### Other observed phenotypes in HLB444

As part of our high-throughput phenotyping platform, numerous other tests were performed on HLB444 animals. Traits found to differ from controls are presented in supporting material (S3 and S4 Figs) and include lower grip strength (S3A Fig), consistent with reduced muscle metabolism reported in *Klf15*^*-/-*^ KO [65]. Further, it is known that during skeletal muscle hypertrophy *Klf15* is downregulated, as are targets of KLF15 that are involved in branched-chain amino acid degradation [66].

The HLB444 colony exhibits a high prevalence of cataracts and retinal defects not seen in age-matched controls (S4 Fig). Histological evaluation found retinal folds and lipid inclusions in HLB444 animals with cataracts. *Klf15* expression has been detected in the retina and KLF15 was found to repress transactivation of rhodopsin and interphotoreceptor retinoid binding protein promoters [67].

## Discussion

We present a new mouse model with a novel single-base mutation in *Klf15* that is resistant to weight gain and hepatosteatosis on a high fat diet. The phenotypes in HLB444 animals are largely concordant with results from a constitutive knockout of *Klf15* [12] and an adipose-specific KO [12], while also similar to some traits reported for an adipose-specific transgenic [15]. Resistance to diet-induced obesity is common to both the full and adipose-specific KO strains and, surprisingly, also to the adipose-specific transgenic strain. Reduced liver triglycerides (i.e., fat) in HLB444 compared to controls on a HF diet suggests that in the liver KLF15 acts similarly to adipocyte KLF15 in balancing lipogenesis and lipolysis in response to nutrient availability [14]. Lack of hepatic triglyceride accumulation in HF-fed HLB444 suggests that this balance is impaired in HLB444. The transgenic strain accumulates two-fold more hepatic triglyceride than controls on a HF diet. Additionally, insulin resistance has been demonstrated for both the transgenic and adipose-specific KO strains [14, 15], while it was not seen in HLB444 or the *Klf15*^*-/-*^ KO [12]. Baseline insulin did not differ from controls for any of the models. With HF feeding, insulin was reduced in HLB444 and increased in the transgenic. Lower plasma triglyceride levels in HLB444 males were also reported for *Klf15*^*-/-*^ KO males (female data not available) on chow diet [68]). Lower plasma glucose was observed in both HLB444 and the *Klf15*^*-/-*^ KO [12], but not in the adipose-specific KO.

Phenotypic similarities among *Klf15* KO strains and HLB444 suggest that the H340Y mutation results in reduced KLF15 function. Differences such as lower cholesterol, HDL-cholesterol and glucose in HLB444 distinguish it from the KO strains, suggesting the H340Y acts as a hypomorph rather than a true null mutation. Additionally, HLB444 did not show the severe hypoglycemia seen in the KO after an overnight fast. KLF15 has been shown to promote fasting-induced gluconeogenesis [12], possibly explaining this response in the KO and further supporting the likelihood that the HLB444 mutation is hypomorphic. Glucose clearance is improved in the full KO, but not in HLB444. In the adipose-specific KO, glucose clearance was not improved and fasting for 6 hours prior to glucose tolerance testing maintained plasma glucose levels of approximately 150 mg/dL. These variations in metabolic phenotypes among *Klf15* genetic models underscore the importance of using multiple novel mutations to further elucidate complexities in transcriptional regulation by KLF15.

Overexpression of *Klf15* in murine heart has been shown to inhibit cardiac hypertrophy [69]. An increased cholesterol:HDL ratio and elevated circulating triglycerides, as observed in HLB444, are risk factors for cardiovascular disease. However, no aberrant cardiovascular phenotypes were found in HLB444, as measured by electrocardiogram and blood pressure tests (S3B and S3C Fig). Jeyaraj and colleagues suggested a critical role for KLF15 in cardiac repolarization linked to circadian rhythms [70], and experiments in a cardiomyocyte-specific *Klf15* KO mouse strain (cK15; [50]) point to KLF15 as a key regulator of diurnal gene expression in the heart. Downregulation of KLF15 has also been found in rodent models of heart failure and in failing human hearts [68]. Expression of KLF15 in the heart has been identified as a key regulator of cardiac lipid metabolism through a mechanism involving recruitment and interaction with *Ep300* [68]. The absence of cardiac phenotypes in HLB444 may be attributable to increased expression of *Klf15* in HLB444 heart, or may suggest that the H340Y mutation has a subtler influence on cardiac KLF15 activity. Our assessments were done only during the light cycle, when mice are less active and further analysis of cardiac phenotypes in this model is warranted.

Members of the Krüppel-like family of transcription factors play diverse and complex physiologic roles as transcriptional activators and repressors. They are distinguished by sharing defined functional motifs, including three C2H2 zinc-finger DNA binding domains that are highly conserved across species [38, 39, 67, 71]. The H340Y mutation in HLB444 disrupts the first of these motifs. The presence of a single altered zinc finger domain may alter some but not all functions of KLF15 in regulating expression of its target genes. Repressor activity of KLF15 has been localized to N-terminal amino acids 68–265 in a luciferase assay, and without these residues KLF15 acted as a transcriptional activator [67]. Deletion of all three zinc-finger domains abolished KLF15 activity [67]. Transcriptional activity of sequential deletions of the C2H2 domains has not been reported, but a nuclear localization signal has been identified within the second and third zinc-finger domains [69]. Further defining the impact of the H340Y mutation on *Klf15* function will help identify targets of this important transcriptional regulator and may shed light on the roles for zinc finger binding in the KLF family of regulatory proteins. Certainly, additional studies are warranted to more clearly resolve the broad phenotypic effects of the H340Y mutation in *Klf15* and better understand its biological consequences. Such additional studies could include evaluating the tissue distribution of KLF15 protein expression in HLB444, especially in adipose, liver and heart. Additional characterization of the functional consequences of the altered first zinc finger motif on binding to direct targets of KLF15 is also needed.

Hepatic gene expression was recently examined in a liver-specific *Klf15* knockout mouse (Li-KO; [72]). Compared to controls, differentially expressed genes in the Li-KO were enriched for pathways related to drug metabolism, including xenobiotic metabolism, as well as in fatty acid biosynthesis and branched-chain amino acid metabolism, in agreement with our findings. Many of those genes were further examined and showed either increased or decreased expression compared to controls, including *Cyp2b9* that was strongly over-expressed in HLB444 animals and in the Li-KO. The Li-KO study specifically highlights a previously under-appreciated role for KLF15 in regulating metabolism and elimination of endobiotics and xenobiotics. Further interrogation of gain or loss of hepatic function in HLB444 livers, including challenge with toxicants or bile duct ligation, would help elucidate impacts of the H340Y mutation.

Differences in gut microbial profiles between HLB444 and controls may be a result of altered pathways of energy metabolism in HLB444 mice that result in shifts in the available nutrients in the gut. Alterations in KLF15 activity targeting metabolic tissues may lead to a differential gut community structure through alterations in microbial metabolites, short chain fatty acid production and inflammatory profiles. Typically, animals fed a standard chow diet exhibit lower Firmicutes to Bacteroidetes ratios than animals on HFD (10, 11). The attenuated increase in the Firmicutes to Bacteriodetes ratio in HLB444 compared to controls in response to HFD suggests a microbial phenotype that is more indicative of normal gut homeostasis ([73]). In previous studies, *Akkermansia* has been associated with healthier metabolic status ([74]). In contrast, we found low *Akkermansia* abundance in both chow- and HFD-fed HLB444 animals, but elevated *Akkermansia* abundance in HFD-fed B6 animals. Increased plasma triglycerides, smaller adipocyte size and lack of hepatosteatosis in HLB444 suggests this strain harbors alterations in host energy harvest and fat absorption.

Alternatively, the HLB444 microbiome might reinforce or exacerbate differences in metabolite profiles that depend primarily on the host genotype. Co-housing or fecal transfer experiments could be used to attempt to distinguish between these possibilities. The altered response to high fat feeding implies that significant differences in microbial metabolites will be present in HLB444 animals. Characterization of the transcriptional effects of the H340Y mutation in liver, fat, and gut tissue may help clarify whether altered metabolism and obesity resistance are direct effects of altered KLF15 target regulatory activities or indirectly a result of altered microbial community composition and the resulting differences in metabolic products. More than likely, the dynamic relationship between gut microbes and their non-linear interactions with host genotype promote the differential gut community structure observed in HLB444.

Since the first KLF was discovered, mouse models with nullified or overexpressed *Klf* genes have been instrumental in understanding the complex functions of this family of regulatory proteins. Functional annotation of known genes is accelerated by investigating novel mutations. We demonstrate that the HLB444 mouse model has particular metabolic phenotypes that distinguish it from mice engineered to either overexpress or eliminate KLF15. Allelic series will help to dissect functional specificities. We anticipate that the H340Y KLF15 mutant strain will provide an additional tool for further investigation of the complex transcriptional regulatory roles of KLF15, especially in metabolic and cardiac function.

## Supporting information

S1 Fig Metabolic cage data. (PDF)

S2 Fig Relative abundance of all OTUs described in Fig 6E. (PDF)

S3 Fig Other phenotypes in HLB444. (PDF)

S4 Fig Cataracts and retinal abnormalities in HLB444. (PDF)

S1 Table Liver gene set enrichment (GSEA). (PDF)

S2 Table Tissue qPCR. (PDF)

S3 Table 16S descriptive statistics. (PDF)

S4 Table 16S inferential statistics. (PDF)

S1 File Supplemental methods. (PDF)

S2 File T-tests for blood chemistries. (XLSX)

S3 File Euler OTUs. (XLSX)

S4 File T-tests OTU abundance. (XLSX)
