# Supplementary figures and images for: A mutation in mouse Krüppel-like factor 15 alters the gut microbiome and response to obesogenic diet
Source: PLoS One. 2019 Sep 25;14(9):e0222536. doi: 10.1371/journal.pone.0222536 (PMC6760833; doi:10.1371/journal.pone.0222536)

Fig S1

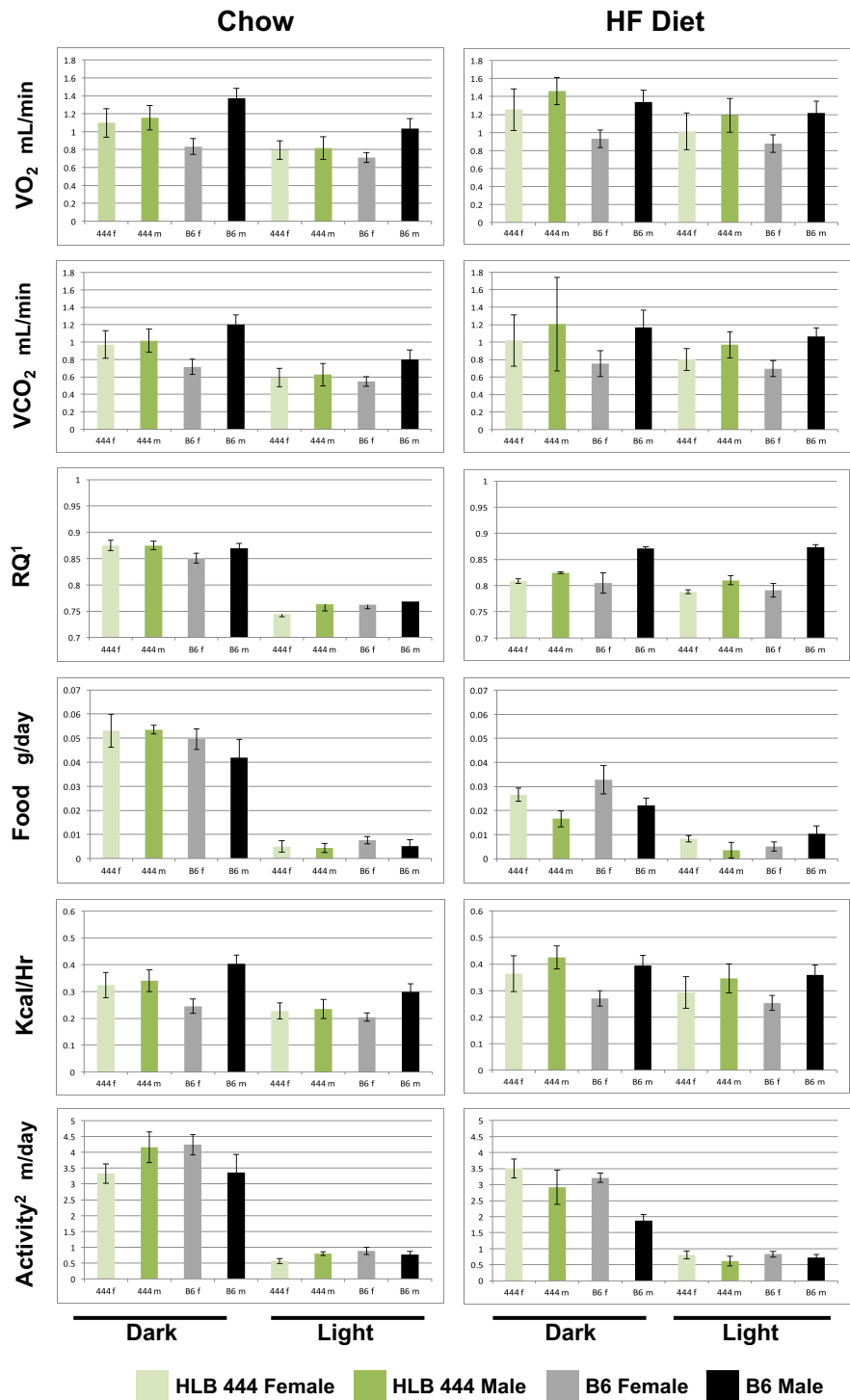

Supplement: S1 Fig — (PDF) [file pone.0222536.s001.pdf]

Supplemental Fig 3

A

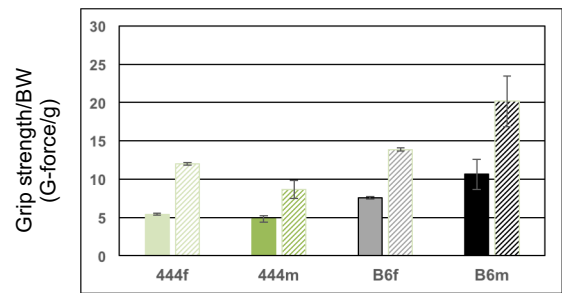

B

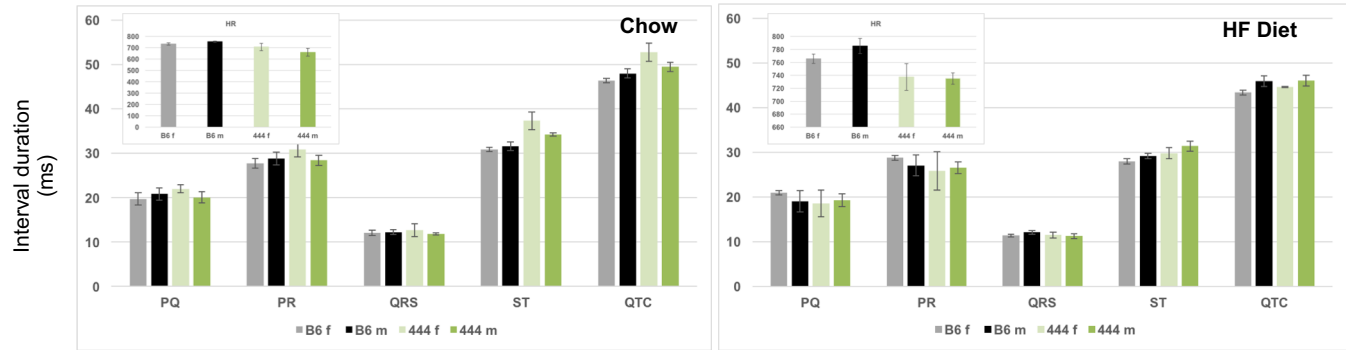

C

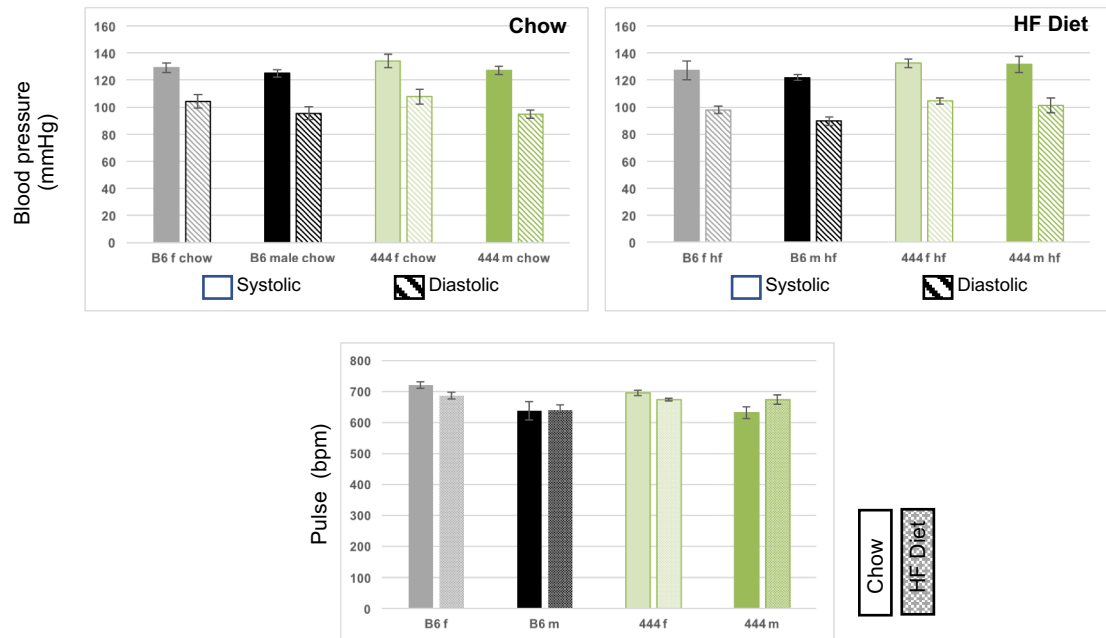

Supplement: S3 Fig — (PDF) [file pone.0222536.s003.pdf]

Supplemental Fig 4

B6

HLB444

A

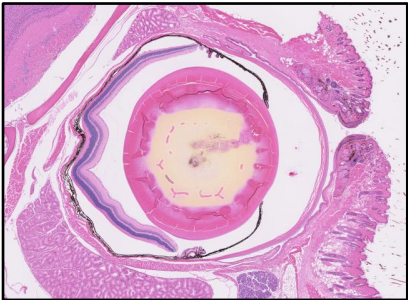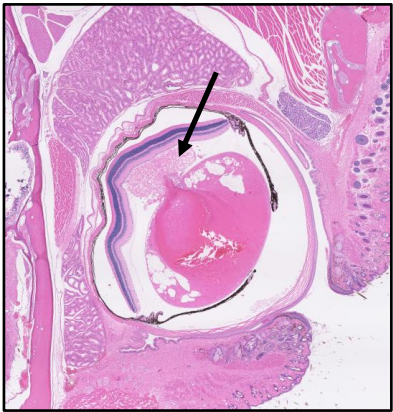

B

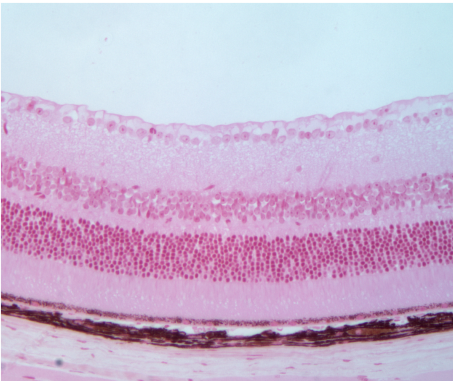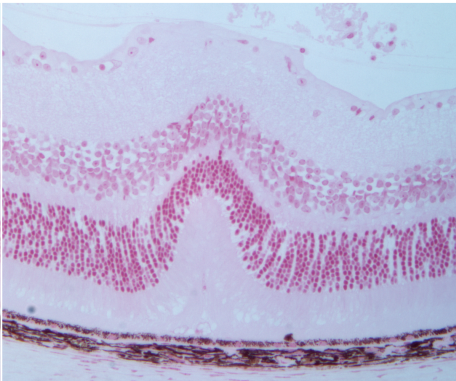

C

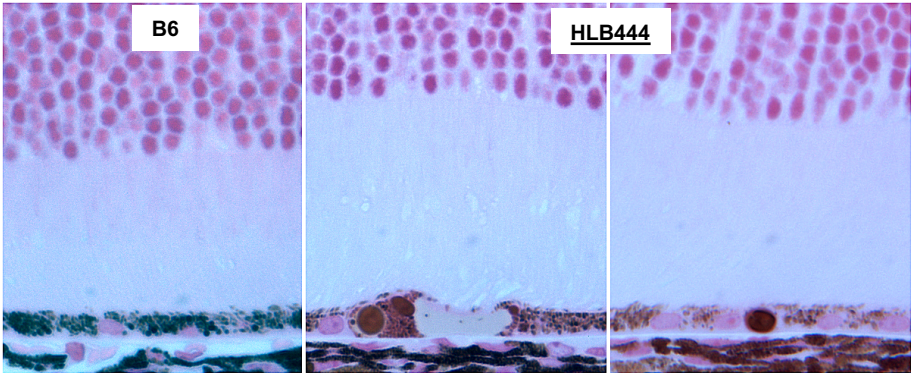

Supplement: S4 Fig — (PDF) [file pone.0222536.s004.pdf]
