# Supplementary material for: A mutation in mouse Krüppel-like factor 15 alters the gut microbiome and response to obesogenic diet
Source: PLoS One. 2019 Sep 25;14(9):e0222536. doi: 10.1371/journal.pone.0222536 (PMC6760833; doi:10.1371/journal.pone.0222536)
Supplement: S2 Fig — (PDF) [file pone.0222536.s002.pdf]

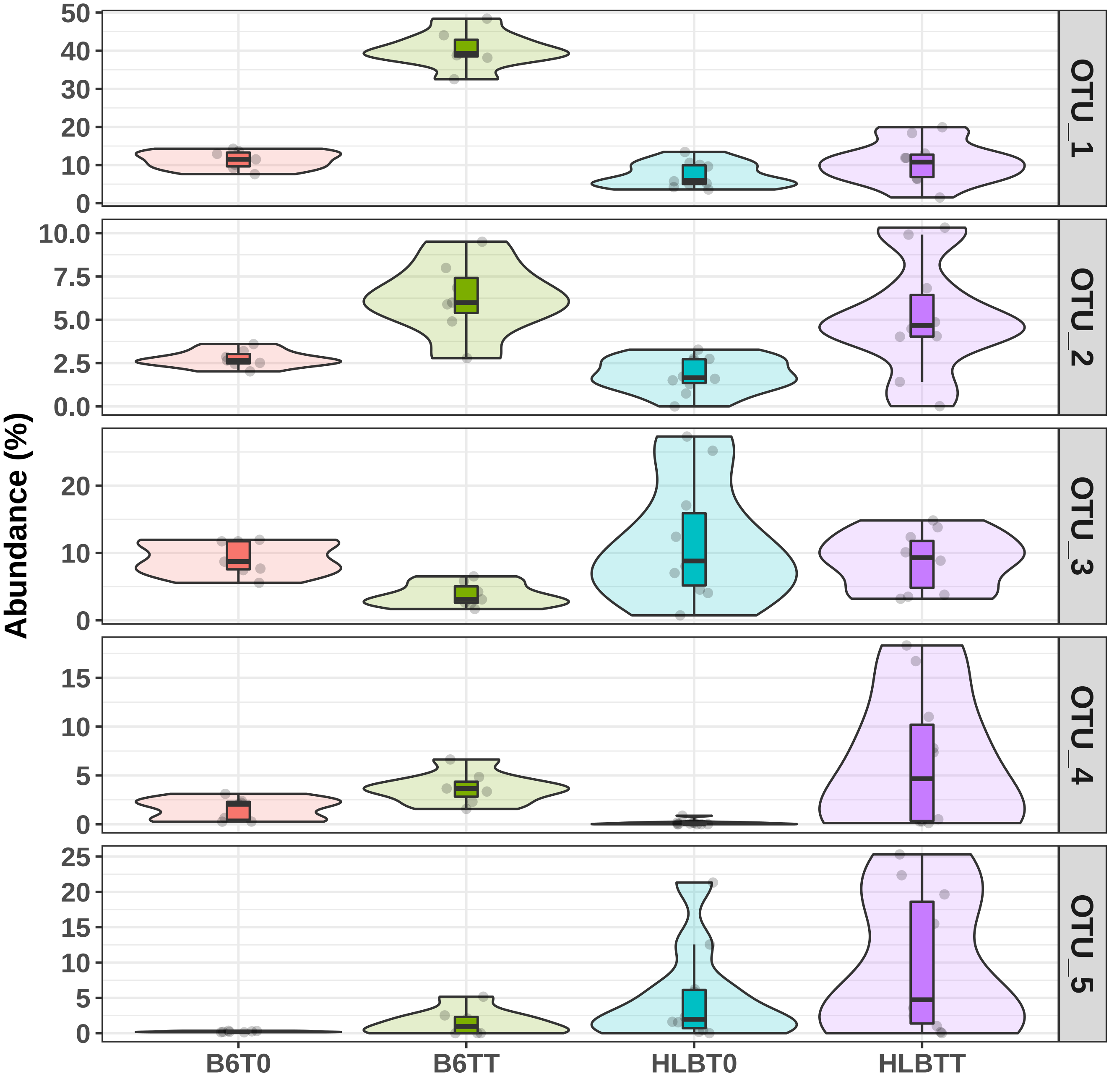
Abundance (%)
OTU_1
OTU_2
OTU_3
OTU_4
OTU_5
B6T0
B6TT
HLBT0
HLBTT

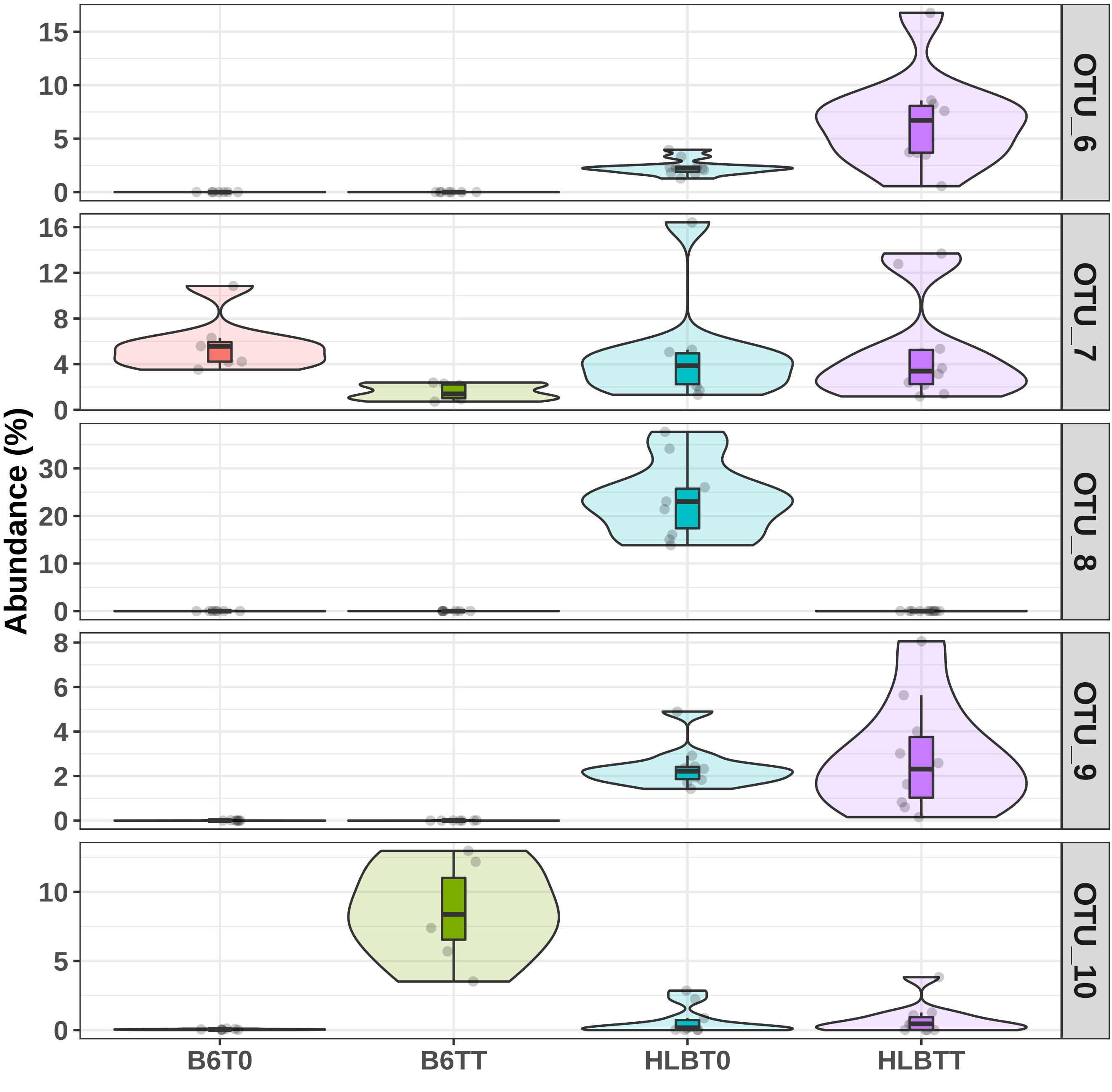

Abundance (%)
OTU_6
OTU_7
OTU_8
OTU_9
OTU_10
B6T0
B6TT
HLBT0
HLBTT
0
5
10
15
0
4
8
12
16
0
10
20
30
0
2
4
6
8
0
5
10

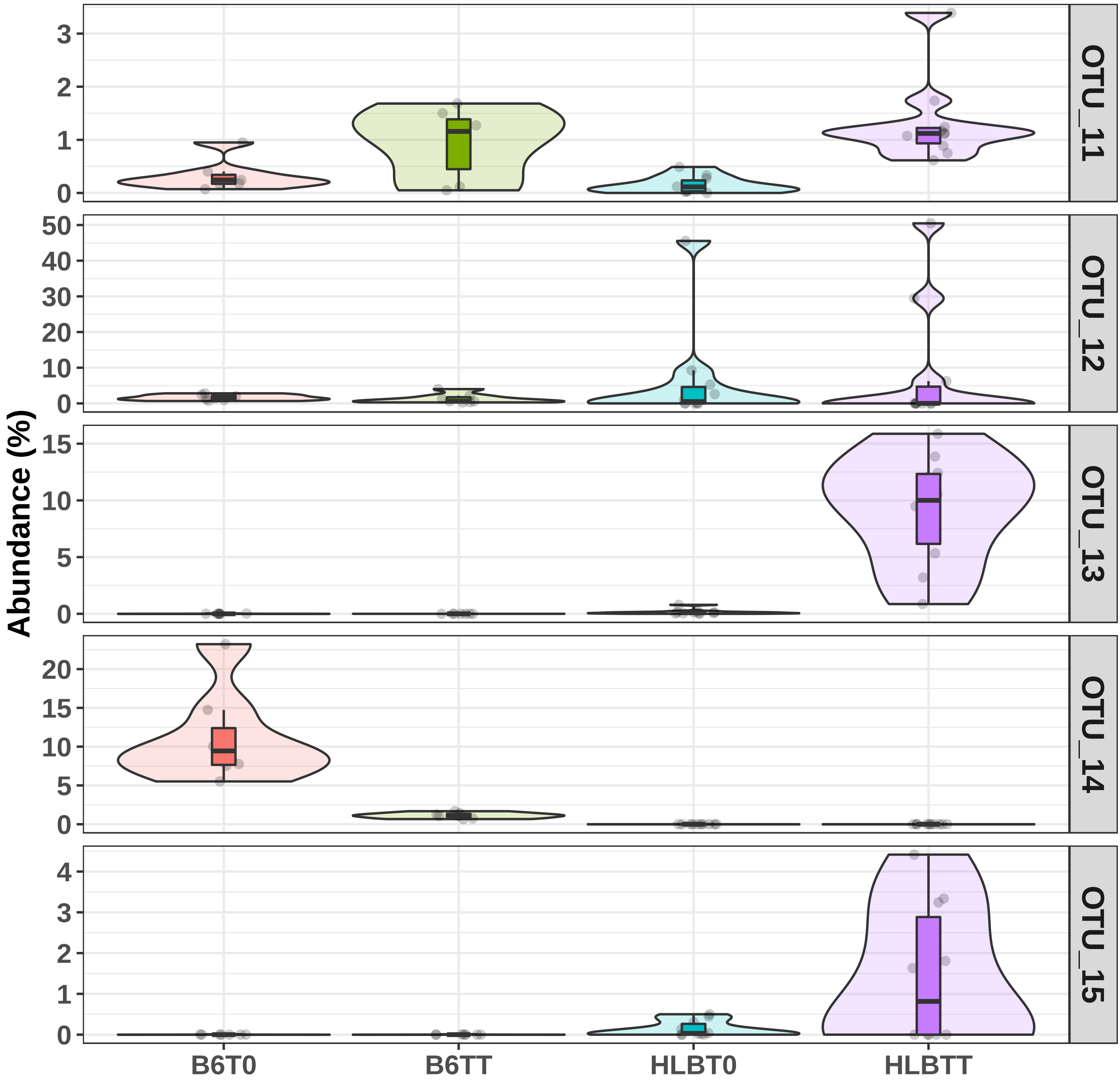

Abundance (%)
OTU_11
OTU_12
OTU_13
OTU_14
OTU_15
B6T0
B6TT
HLBT0
HLBTT

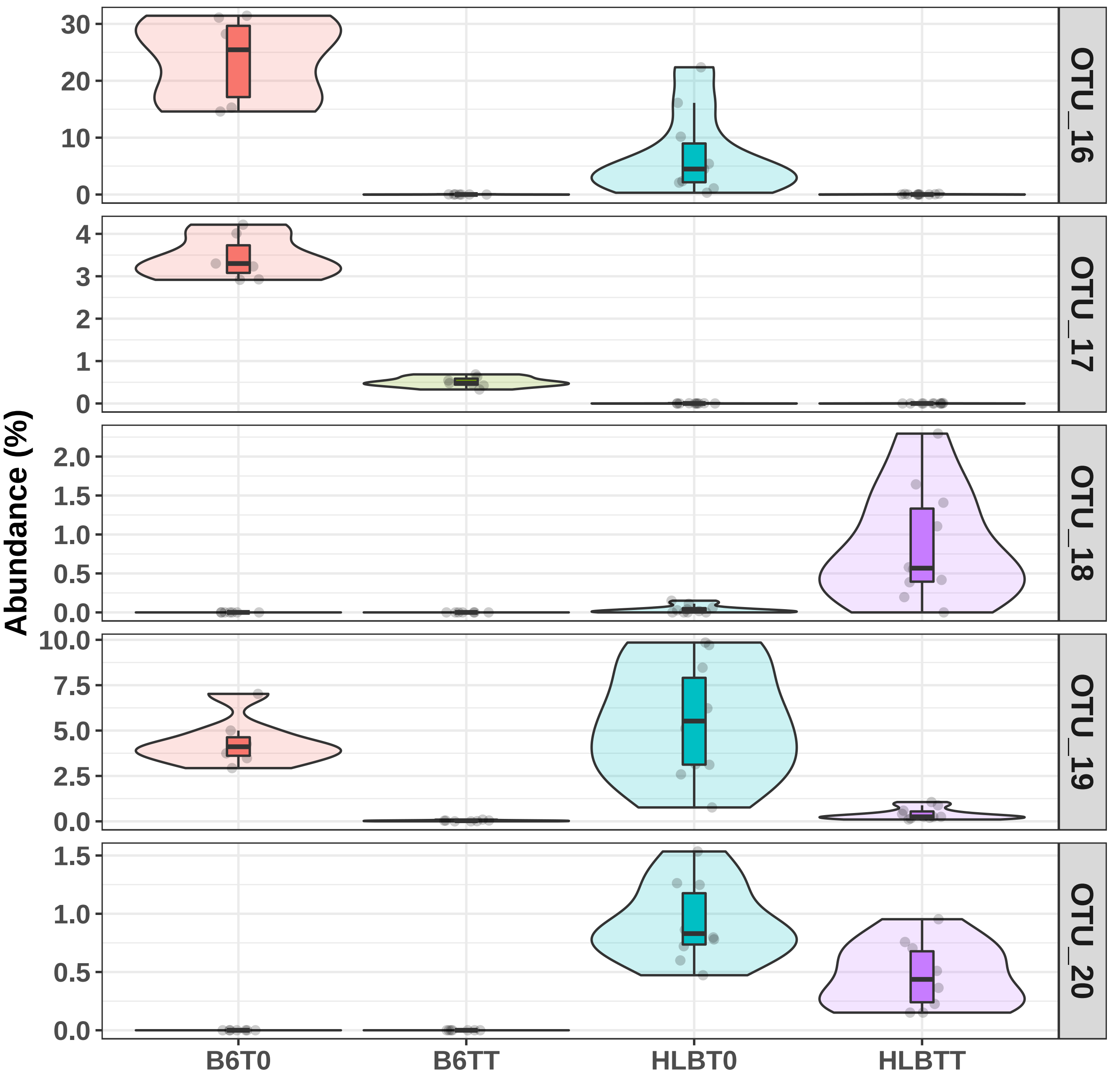

Abundance (%)
OTU_16
OTU_17
OTU_18
OTU_19
OTU_20
B6T0
B6TT
HLBT0
HLBTT
0
10
20
30
0
1
2
3
4
0.0
0.5
1.0
1.5
2.0
0.0
2.5
5.0
7.5
10.0
0.0
0.5
1.0
1.5

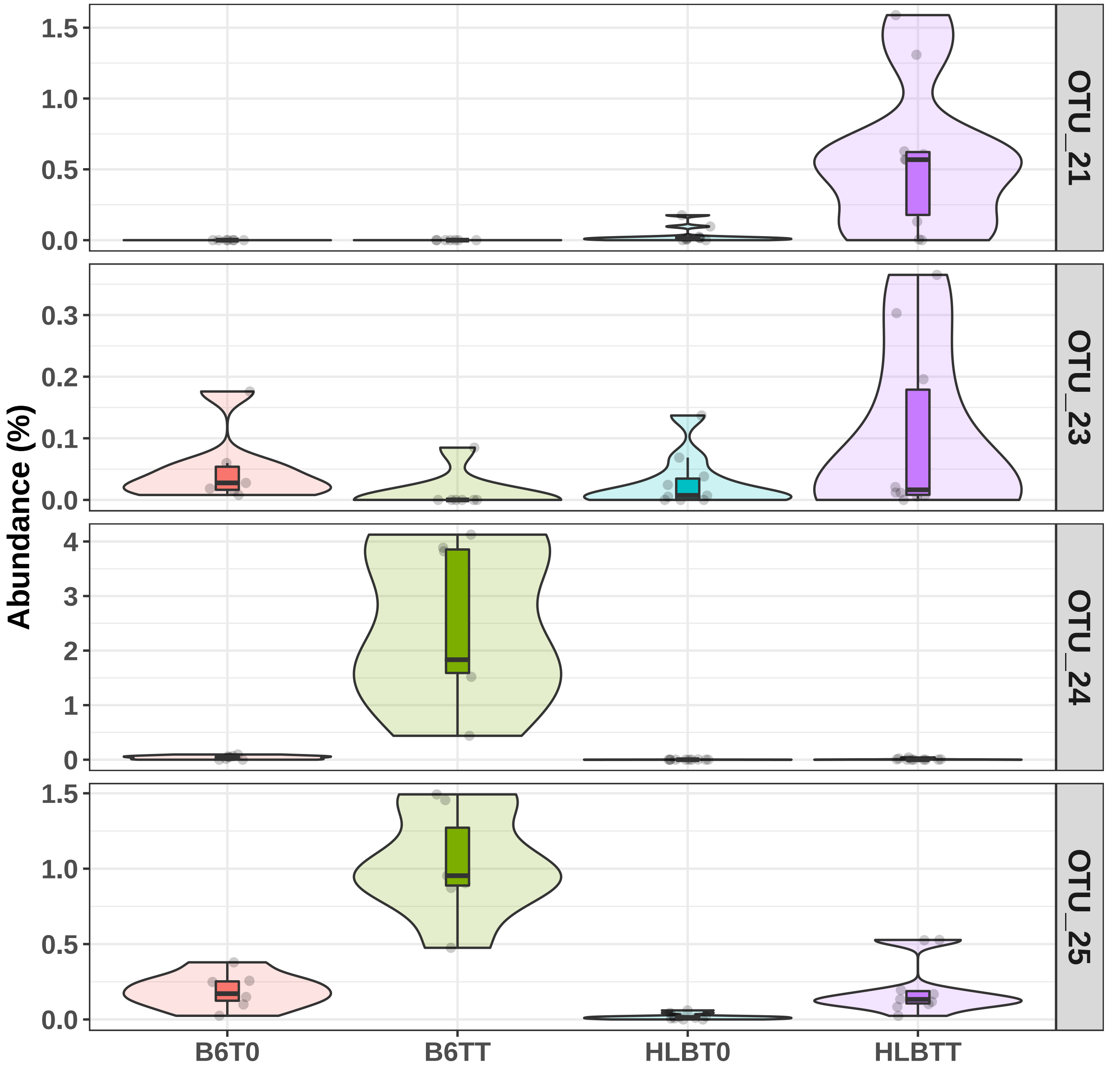

Abundance (%)
OTU_21
OTU_23
OTU_24
OTU_25
0.0
0.5
1.0
1.5
0.0
0.1
0.2
0.3
0
1
2
3
4
0.0
0.5
1.0
1.5
B6T0
B6TT
HLBT0
HLBTT

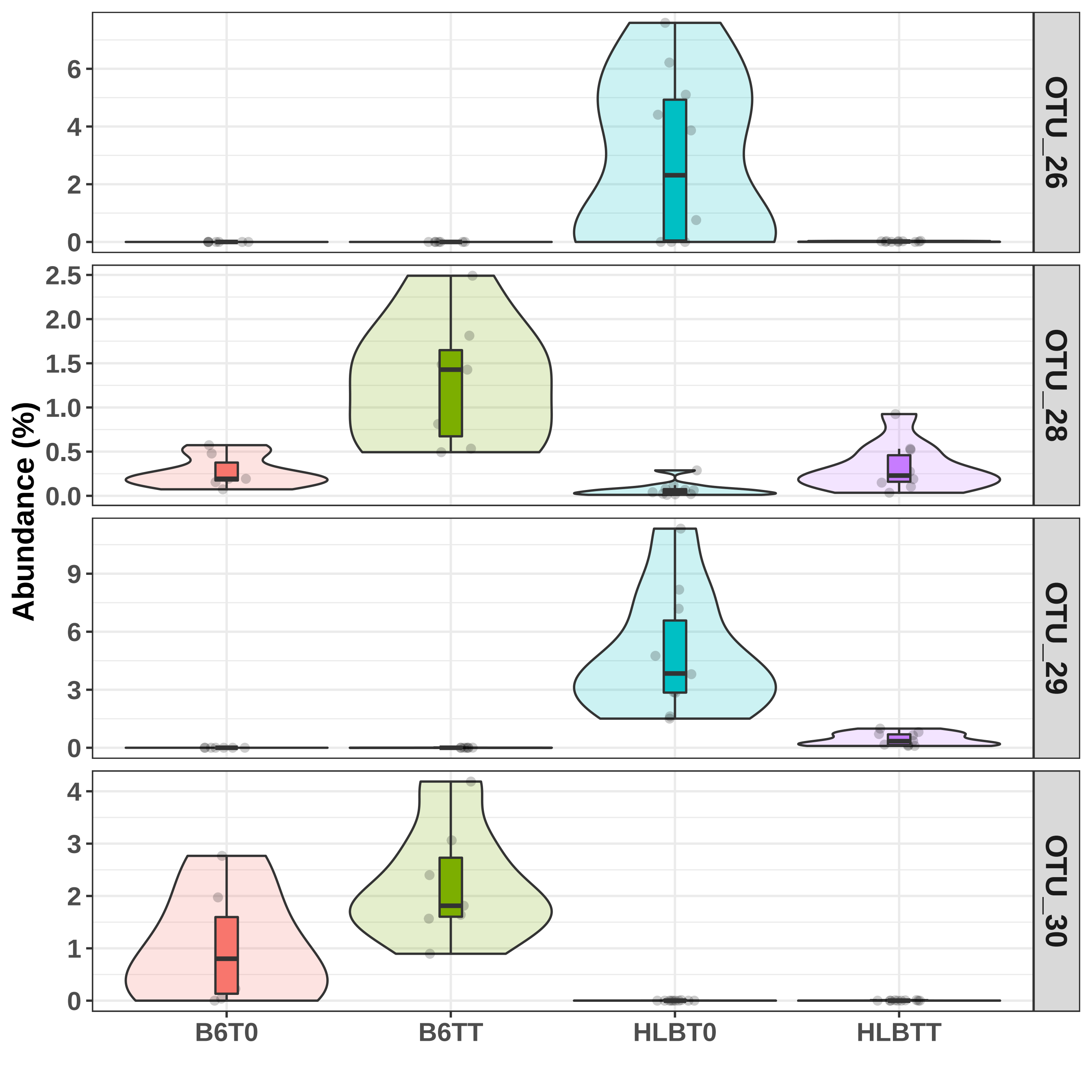

Abundance (%)
OTU_26
OTU_28
OTU_29
OTU_30
0
2
4
6
0.0
0.5
1.0
1.5
2.0
2.5
0
3
6
9
0
1
2
3
4
B6T0
B6TT
HLBT0
HLBTT

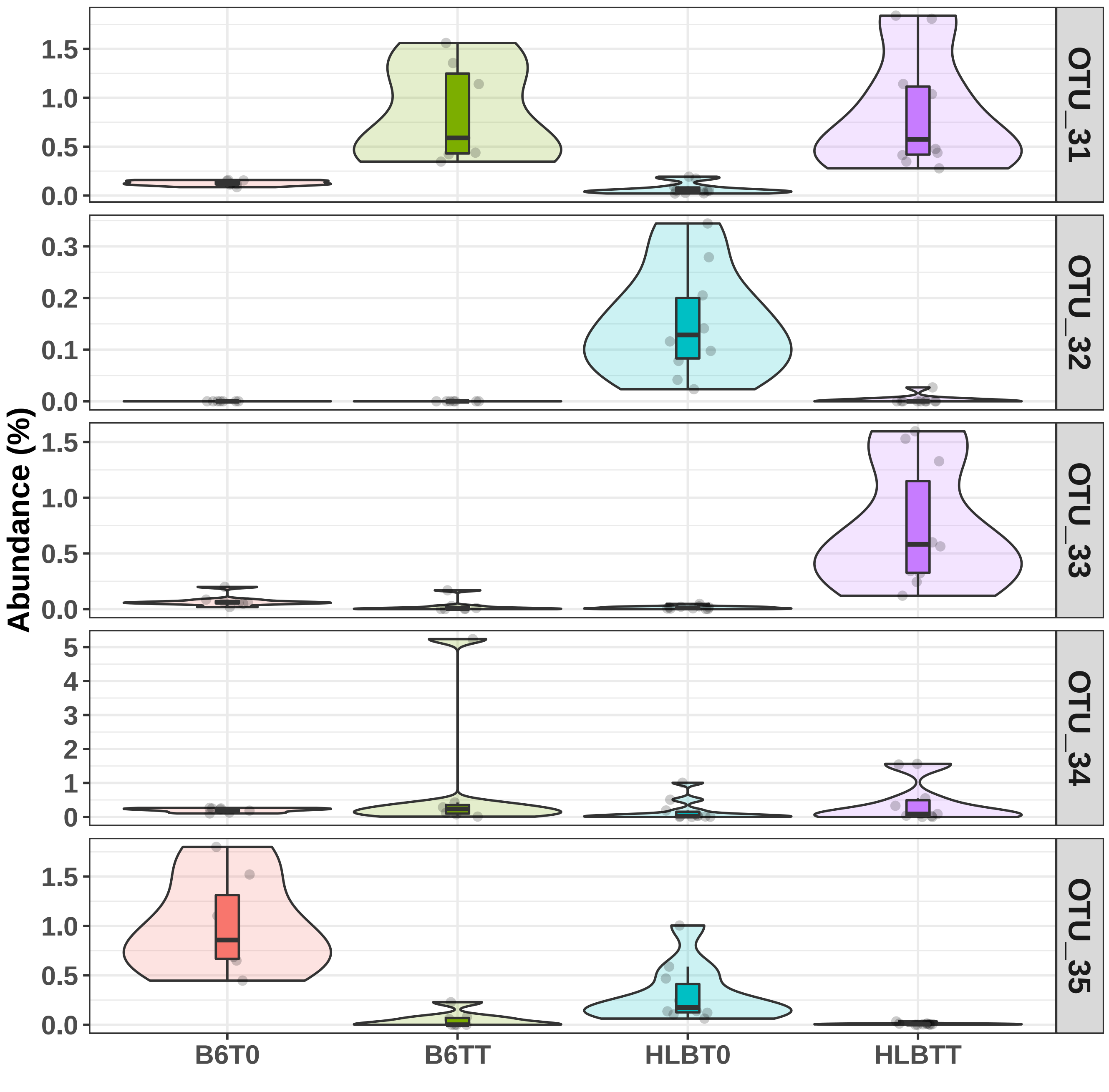
Abundance (%)
OTU_31
OTU_32
OTU_33
OTU_34
OTU_35
B6T0
B6TT
HLBT0
HLBTT

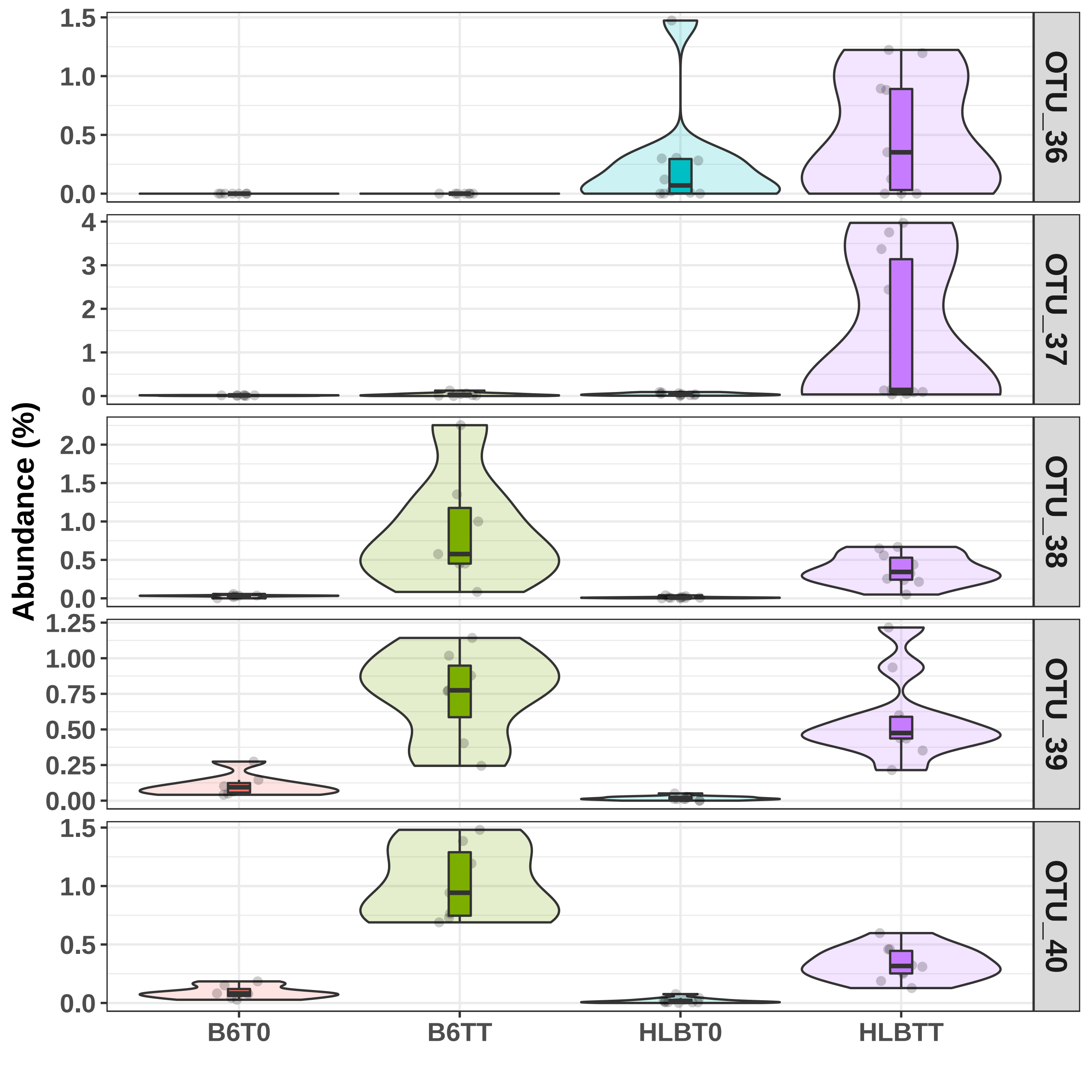

Abundance (%)
OTU_36
OTU_37
OTU_38
OTU_39
OTU_40
B6T0
B6TT
HLBT0
HLBTT

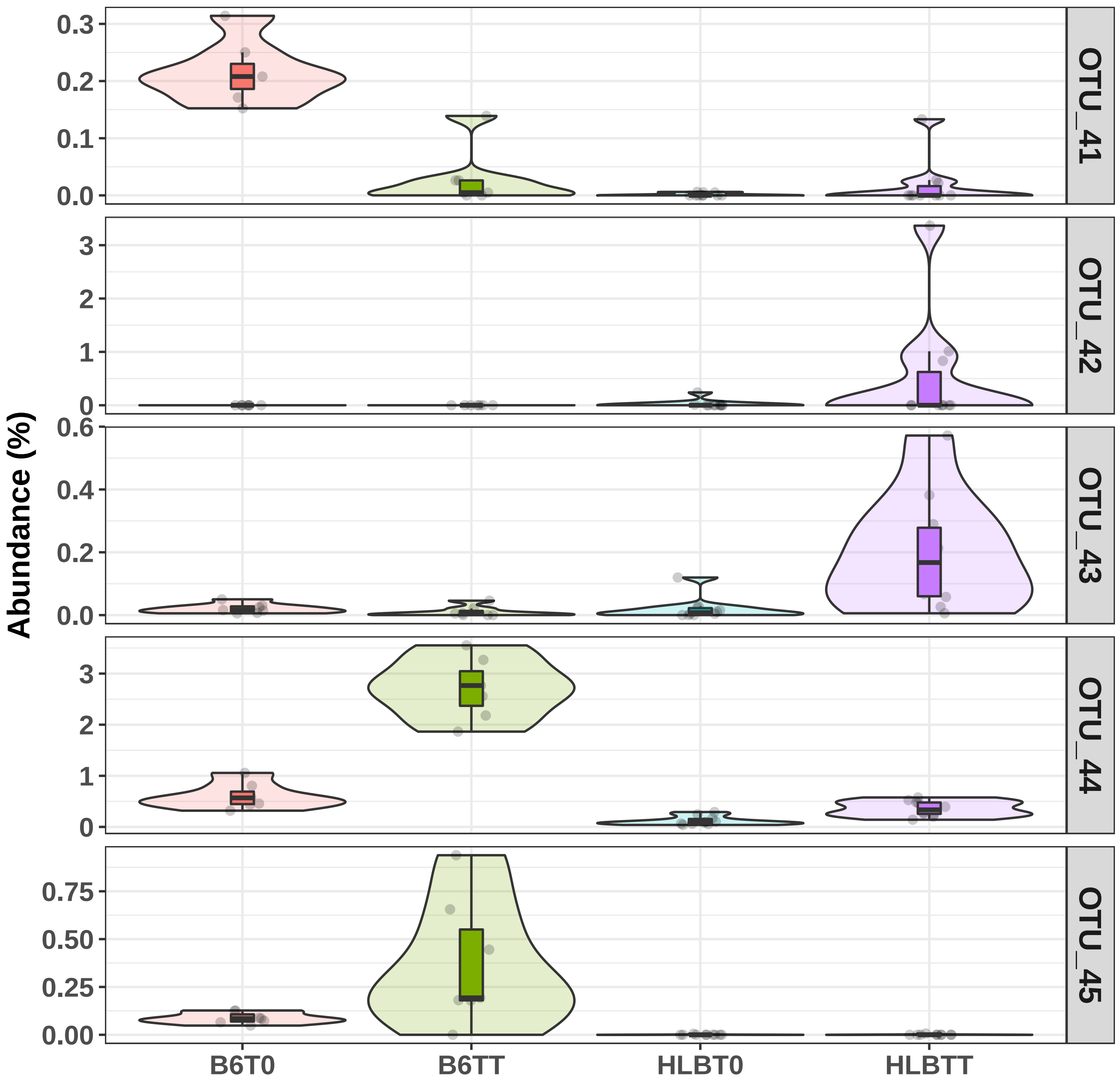
Abundance (%)
OTU_41
OTU_42
OTU_43
OTU_44
OTU_45
B6T0
B6TT
HLBT0
HLBTT

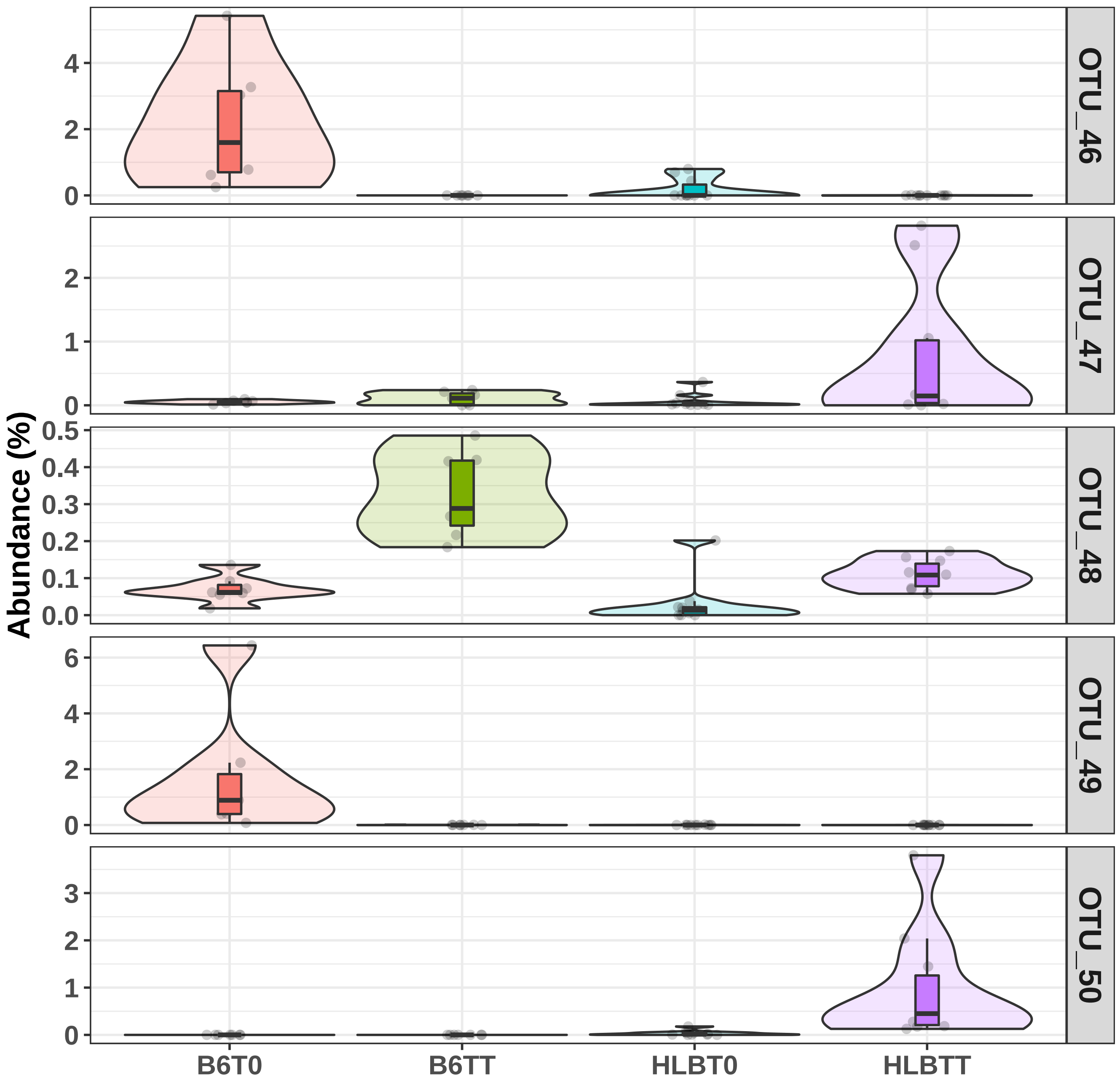
Abundance (%)
OTU_46
OTU_47
OTU_48
OTU_49
OTU_50
B6T0
B6TT
HLBT0
HLBTT

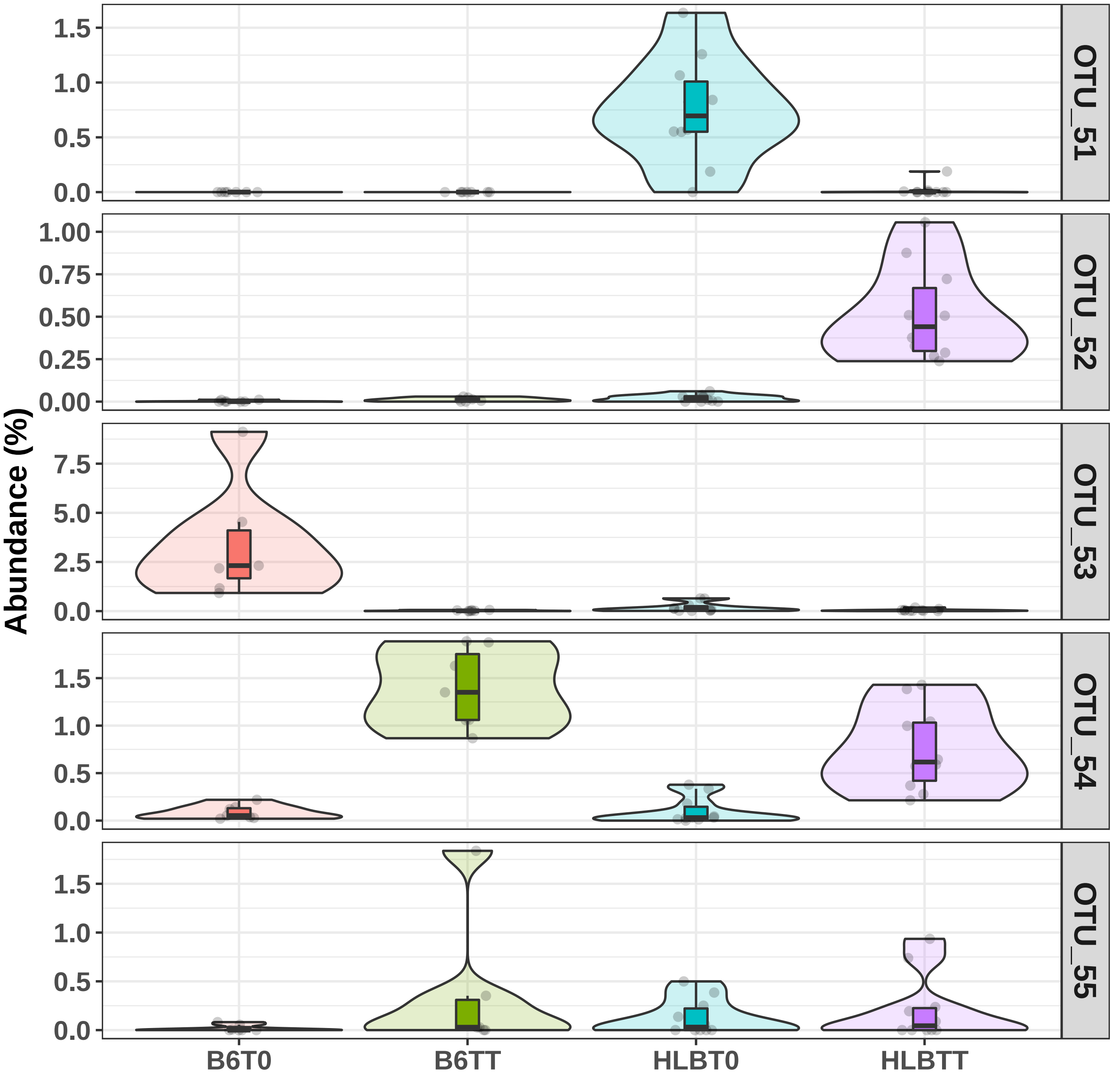

Abundance (%)
OTU_51
OTU_52
OTU_53
OTU_54
OTU_55
B6T0
B6TT
HLBT0
HLBTT

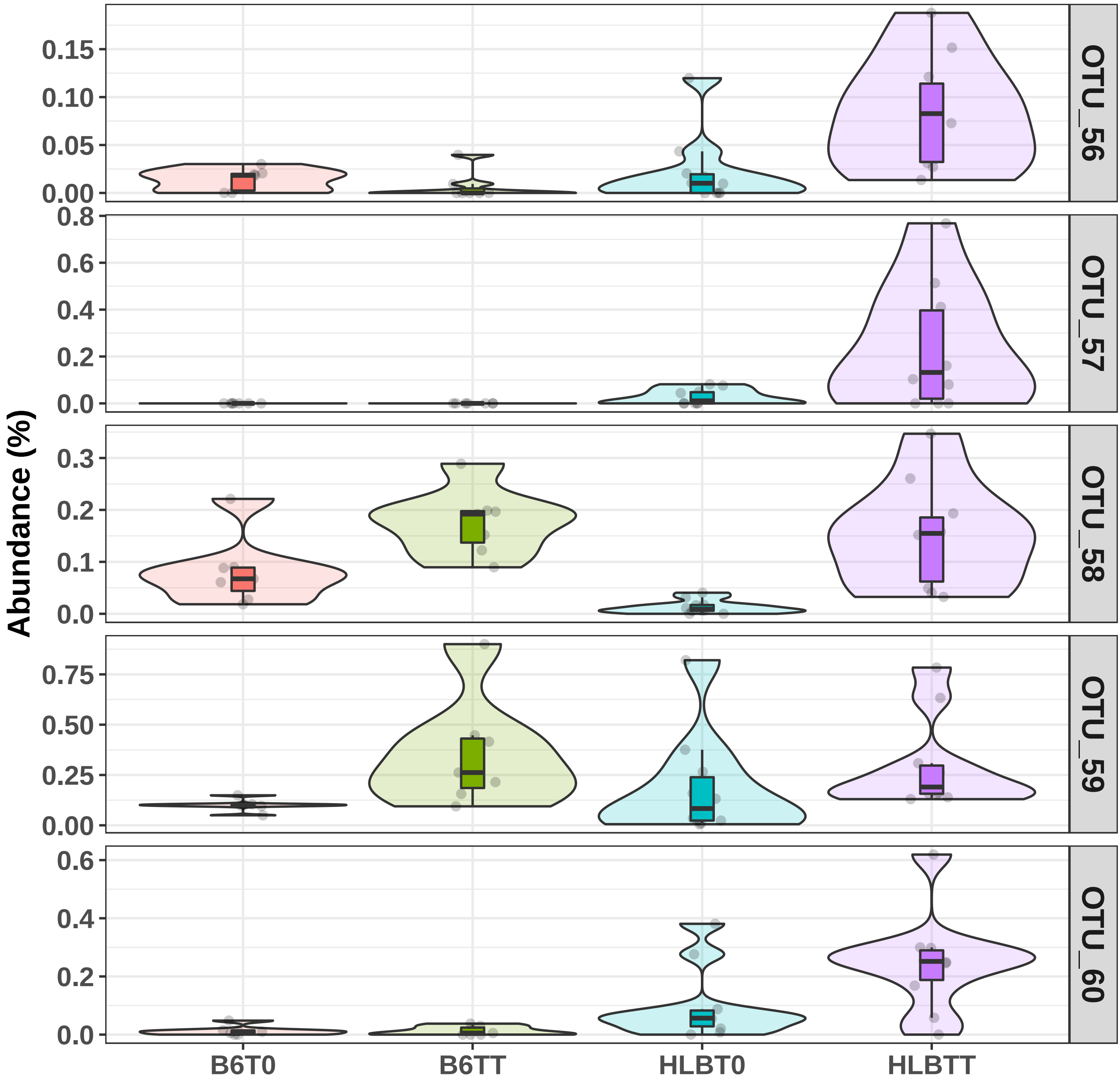

Abundance (%)
0.00
0.05
0.10
0.15
0.0
0.2
0.4
0.6
0.8
0.0
0.1
0.2
0.3
0.00
0.25
0.50
0.75
0.0
0.2
0.4
0.6
OTU_56
OTU_57
OTU_58
OTU_59
OTU_60
B6T0
B6TT
HLBT0
HLBTT

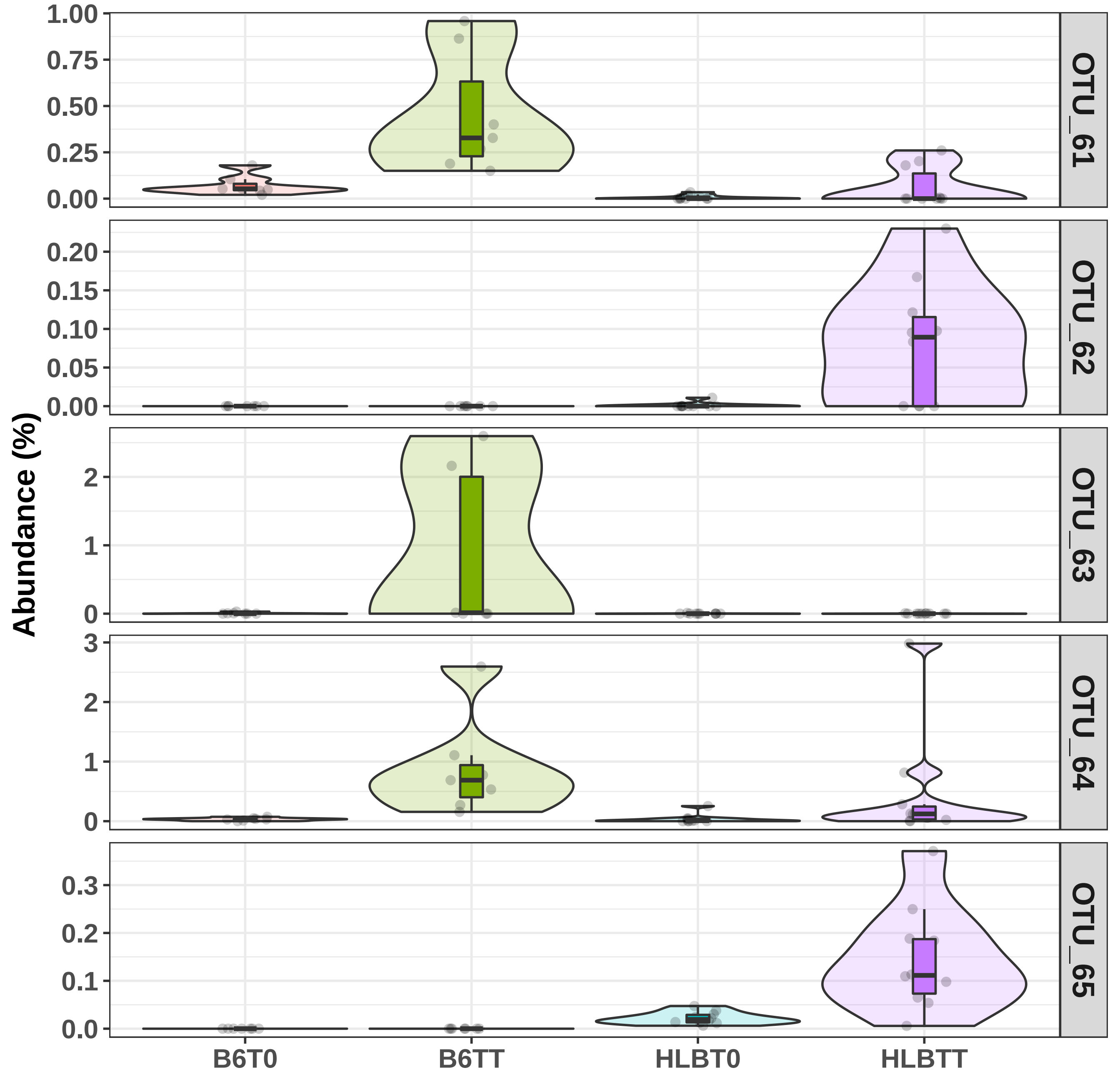
Abundance (%)
OTU_61
OTU_62
OTU_63
OTU_64
OTU_65
B6T0
B6TT
HLBT0
HLBTT
1.00
0.75
0.50
0.25
0.00
0.20
0.15
0.10
0.05
0.00
2
1
0
3
2
1
0
0.3
0.2
0.1
0.0

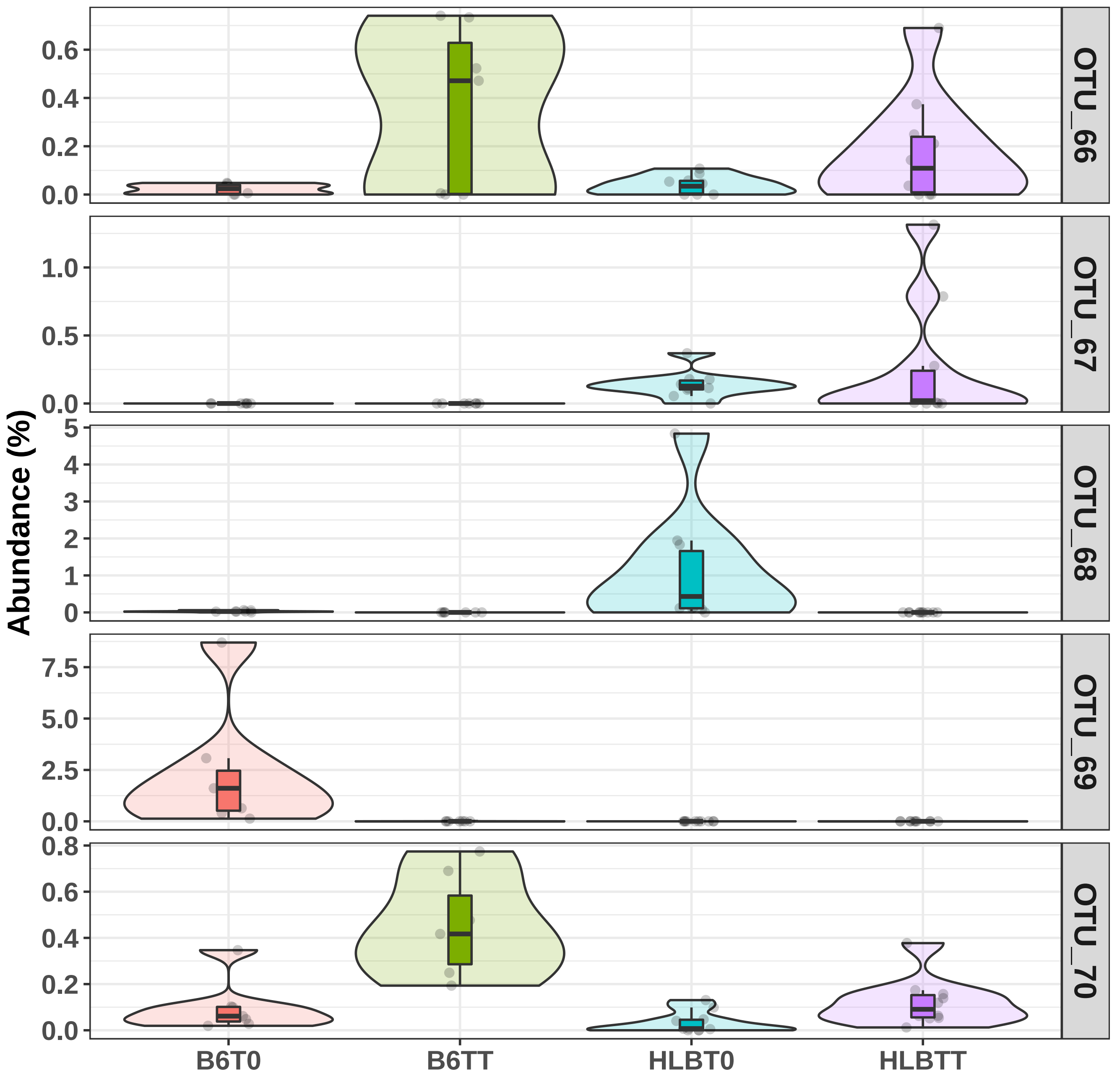

Abundance (%)
OTU_66
OTU_67
OTU_68
OTU_69
OTU_70
B6T0
B6TT
HLBT0
HLBTT

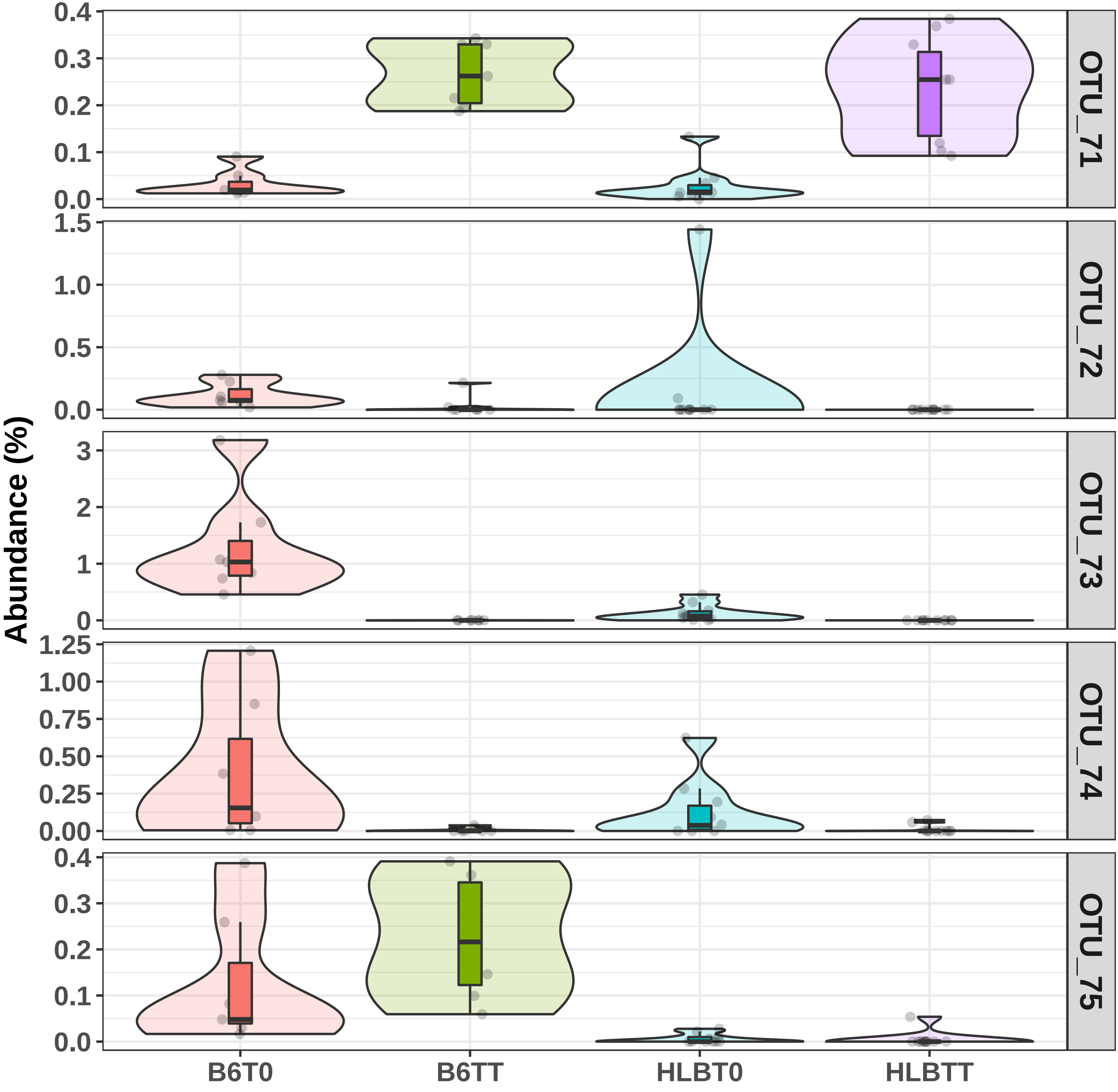

Abundance (%)
OTU_71
OTU_72
OTU_73
OTU_74
OTU_75
0.0
0.1
0.2
0.3
0.4
0.5
1.0
1.5
0
1
2
3
0.00
0.25
0.50
0.75
1.00
1.25
B6T0
B6TT
HLBT0
HLBTT

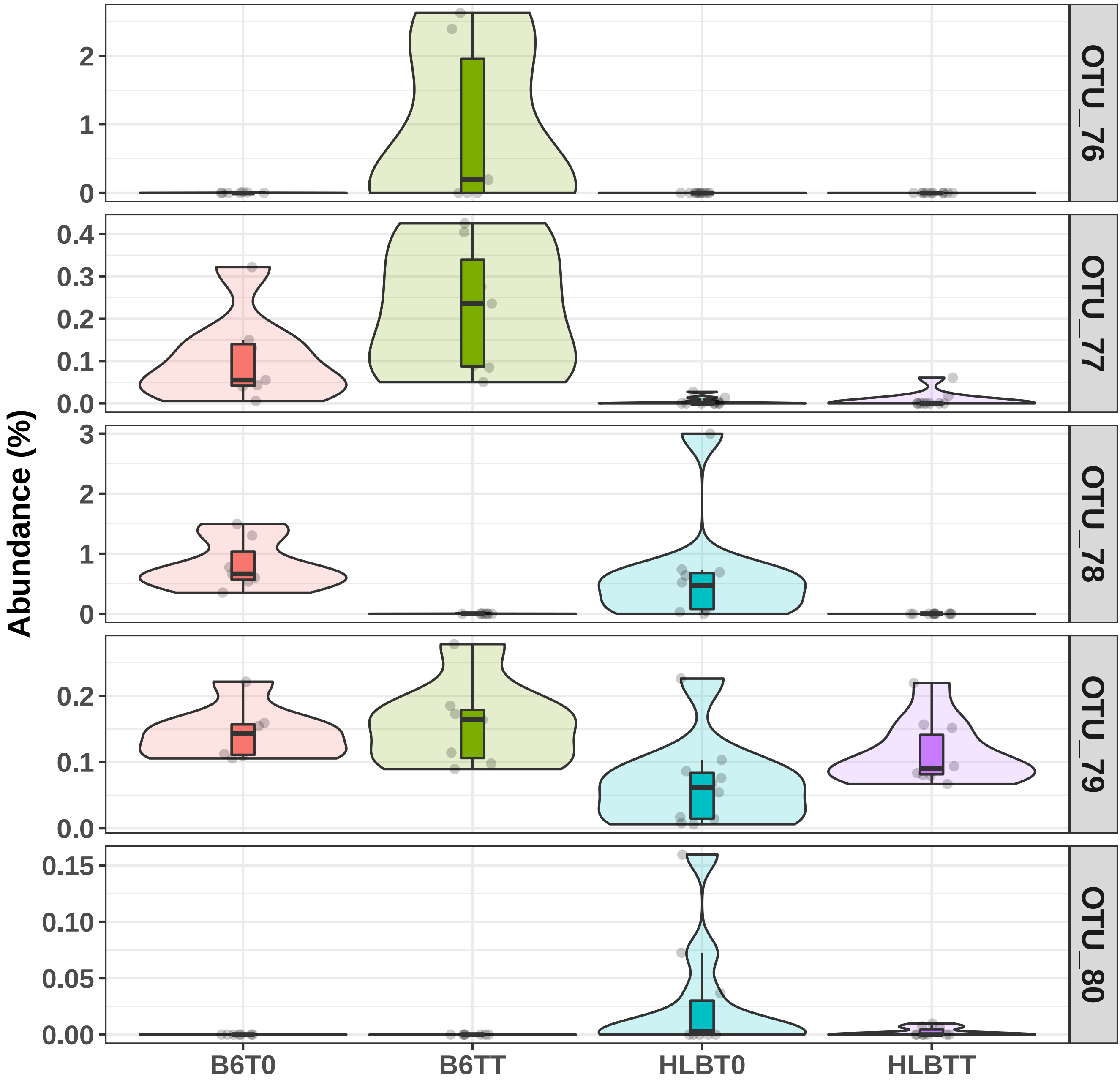

Abundance (%)
OTU_76
OTU_77
OTU_78
OTU_79
OTU_80
0
1
2
0.0
0.1
0.2
0.3
0.4
3
0.00
0.05
0.10
0.15
B6T0
B6TT
HLBT0
HLBTT

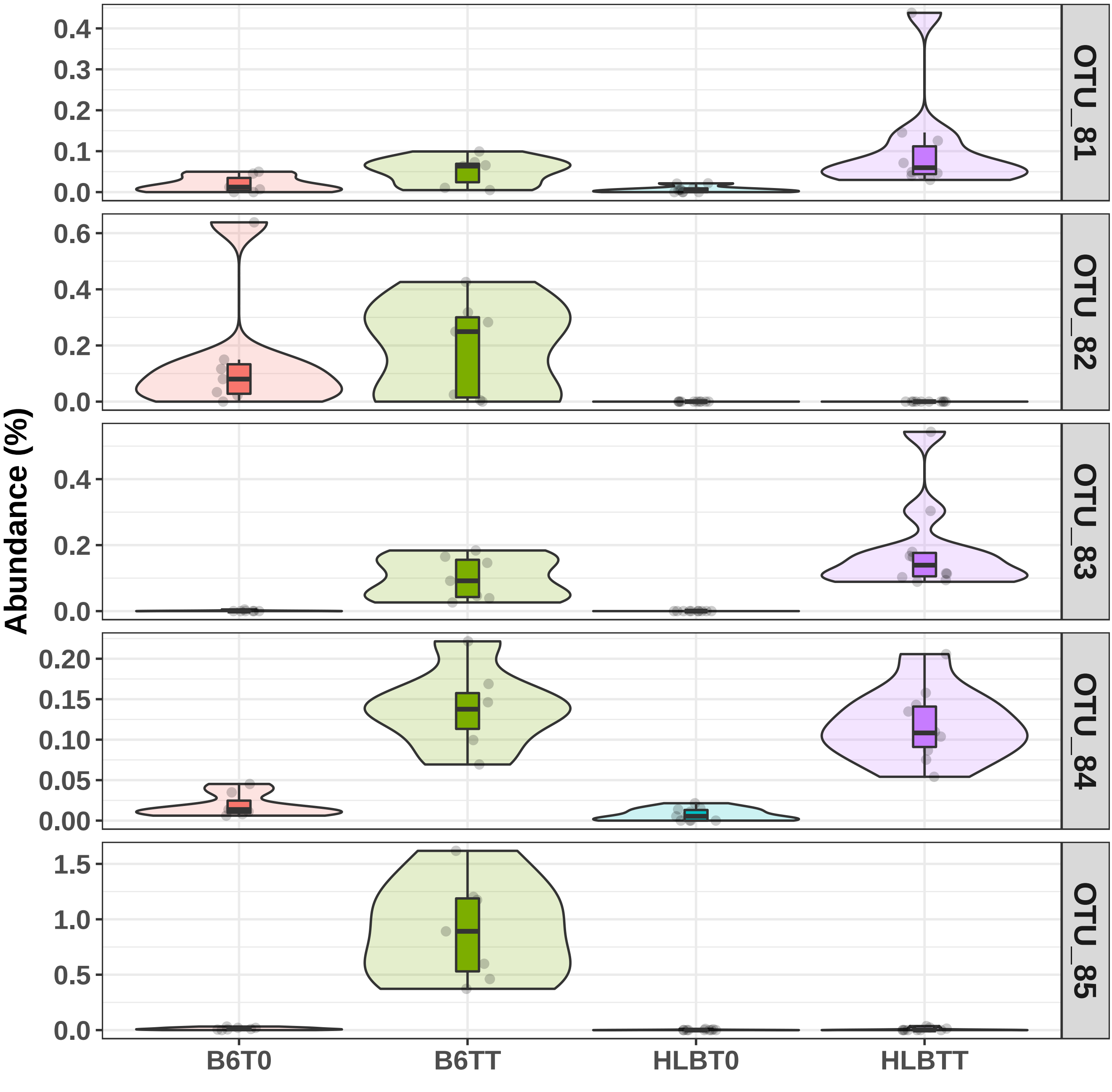
Abundance (%)
OTU_81
OTU_82
OTU_83
OTU_84
OTU_85
0.0
0.1
0.2
0.3
0.4
0.6
0.00
0.05
0.10
0.15
0.20
0.5
1.0
1.5
B6T0
B6TT
HLBT0
HLBTT

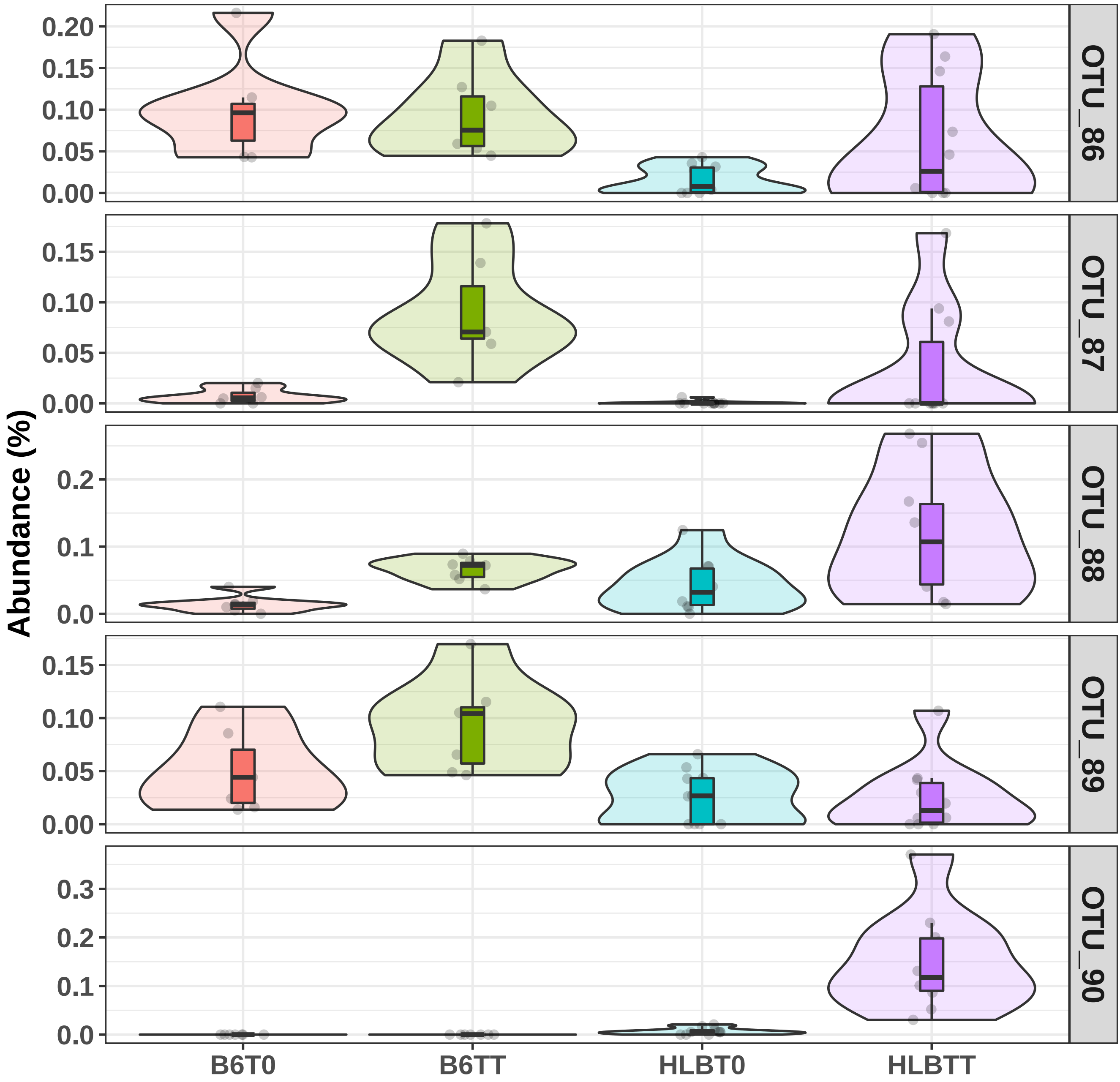
Abundance (%)
OTU_86
OTU_87
OTU_88
OTU_89
OTU_90
B6T0
B6TT
HLBT0
HLBTT

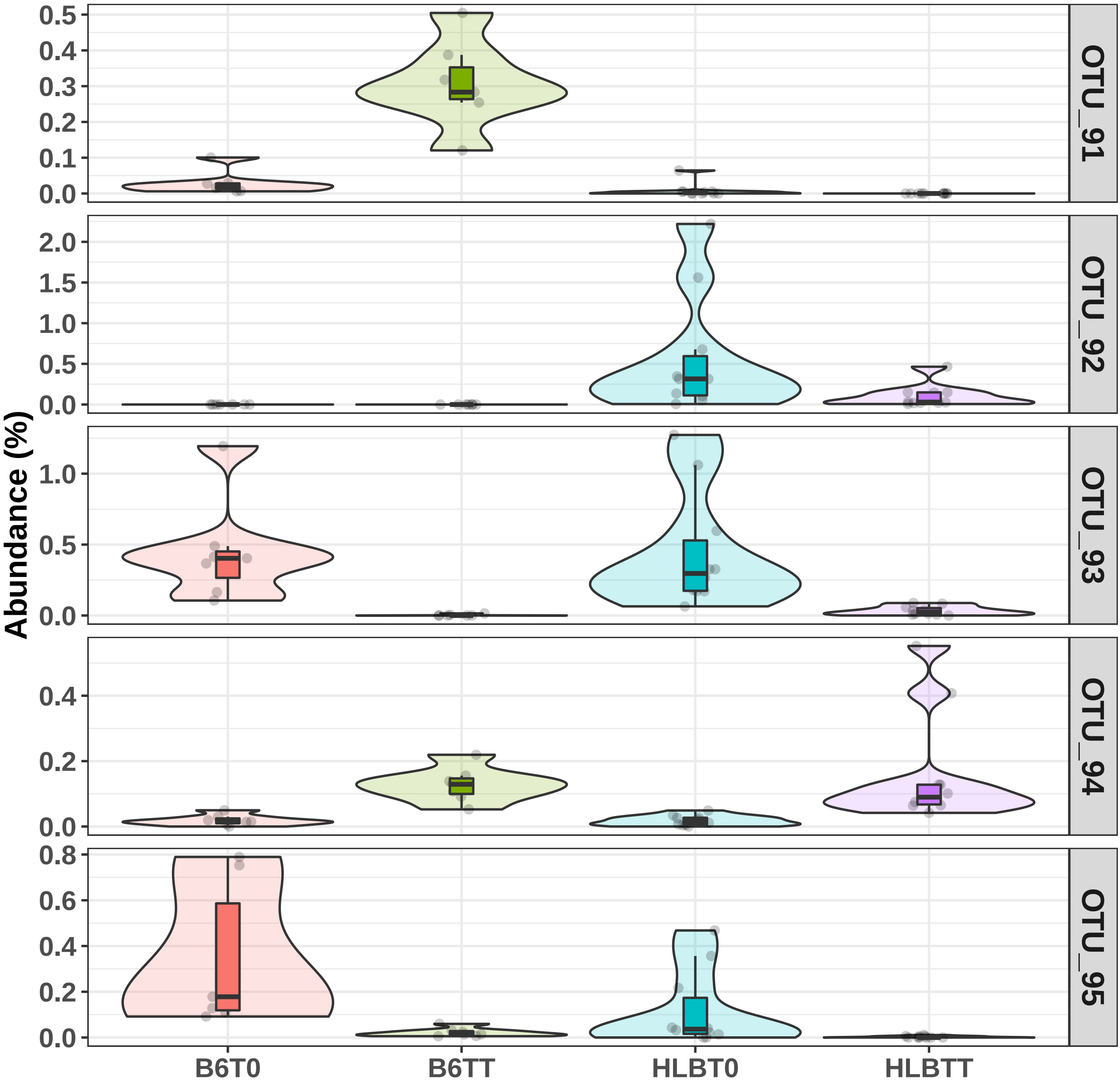
Abundance (%)
OTU_91
OTU_92
OTU_93
OTU_94
OTU_95
B6T0
B6TT
HLBT0
HLBTT

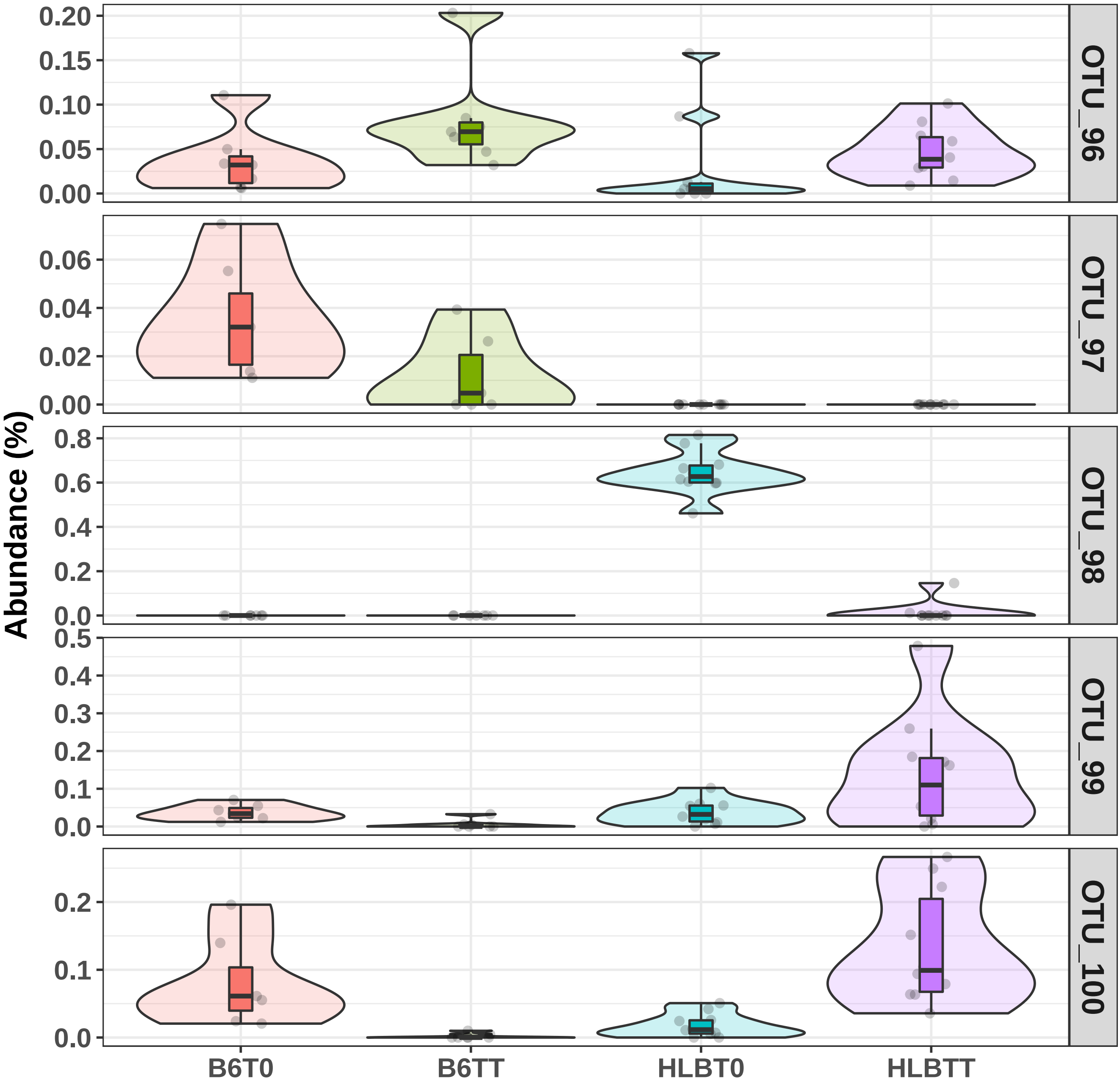

Abundance (%)
OTU_96
OTU_97
OTU_98
OTU_99
OTU_100
B6T0
B6TT
HLBT0
HLBTT
0.00
0.05
0.10
0.15
0.20
0.02
0.04
0.06
0.0
0.2
0.4
0.6
0.8
0.1
0.3
0.5

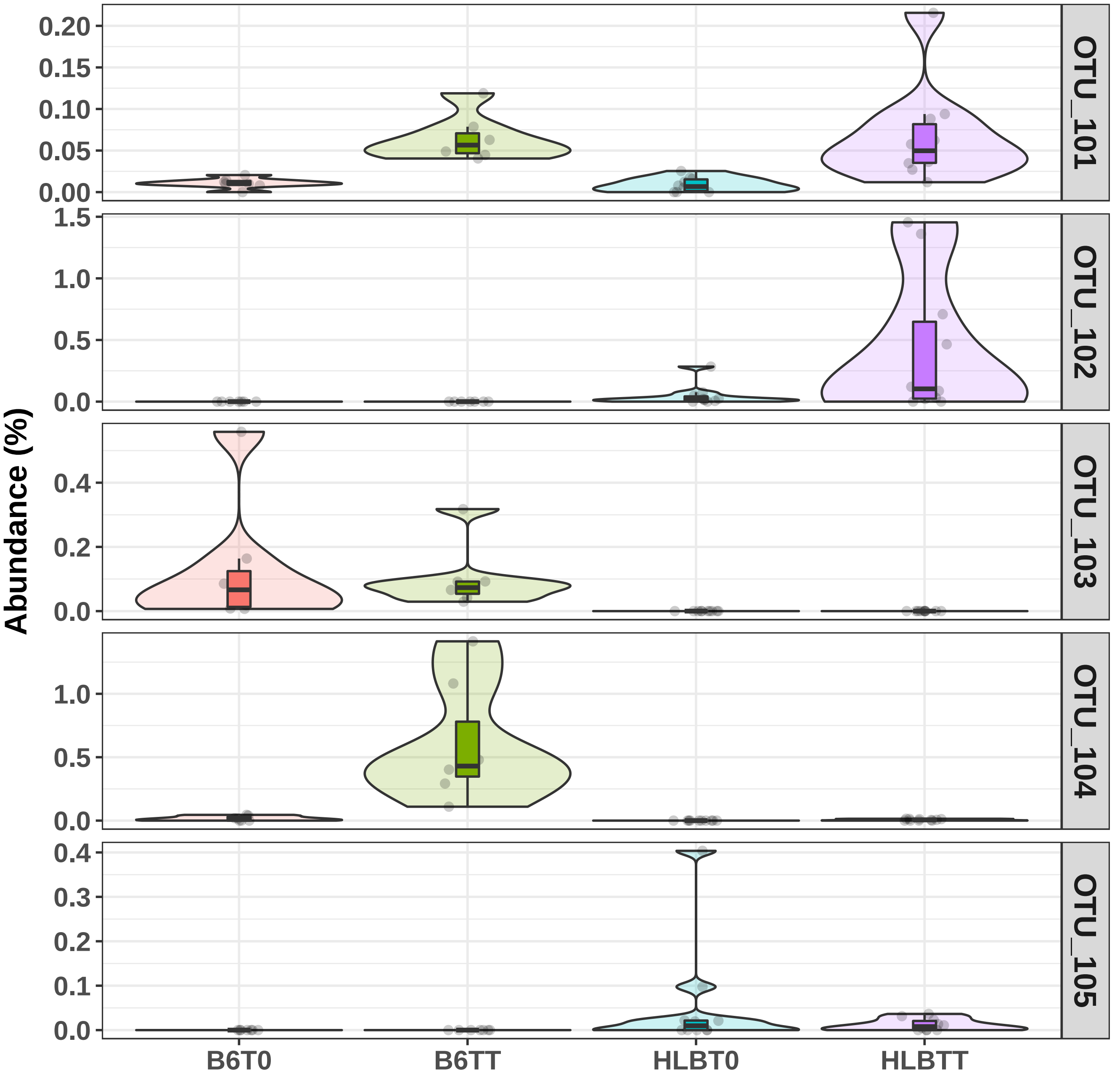

Abundance (%)
OTU_101
OTU_102
OTU_103
OTU_104
OTU_105
0.00
0.05
0.10
0.15
0.20
0.0
0.5
1.0
1.5
0.0
0.2
0.4
0.0
0.5
1.0
0.0
0.1
0.2
0.3
0.4
B6T0
B6TT
HLBT0
HLBTT

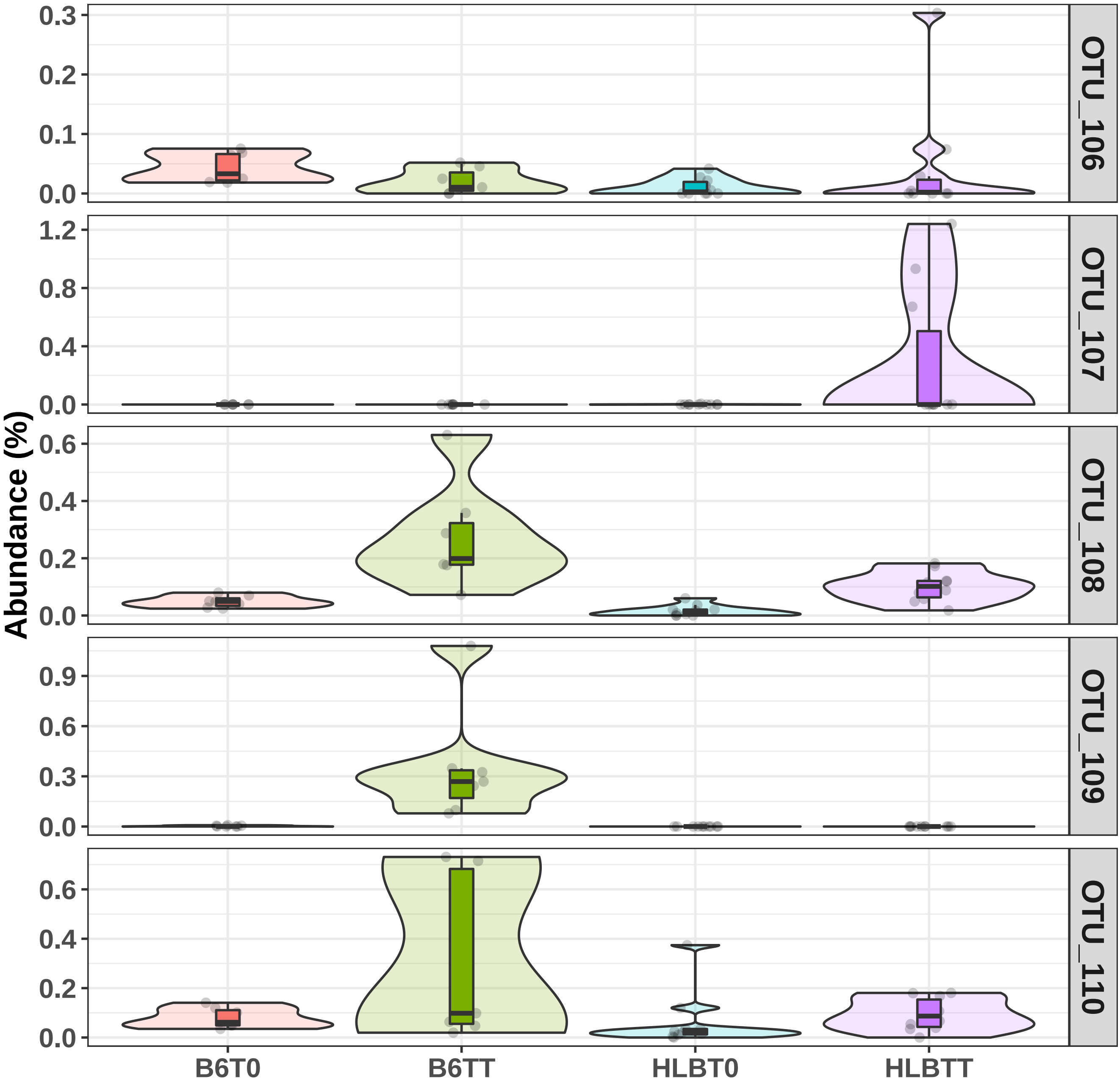

Abundance (%)
OTU_106
OTU_107
OTU_108
OTU_109
OTU_110
B6T0
B6TT
HLBT0
HLBTT
0.0
0.1
0.2
0.3
0.4
0.6
0.8
0.9
1.2

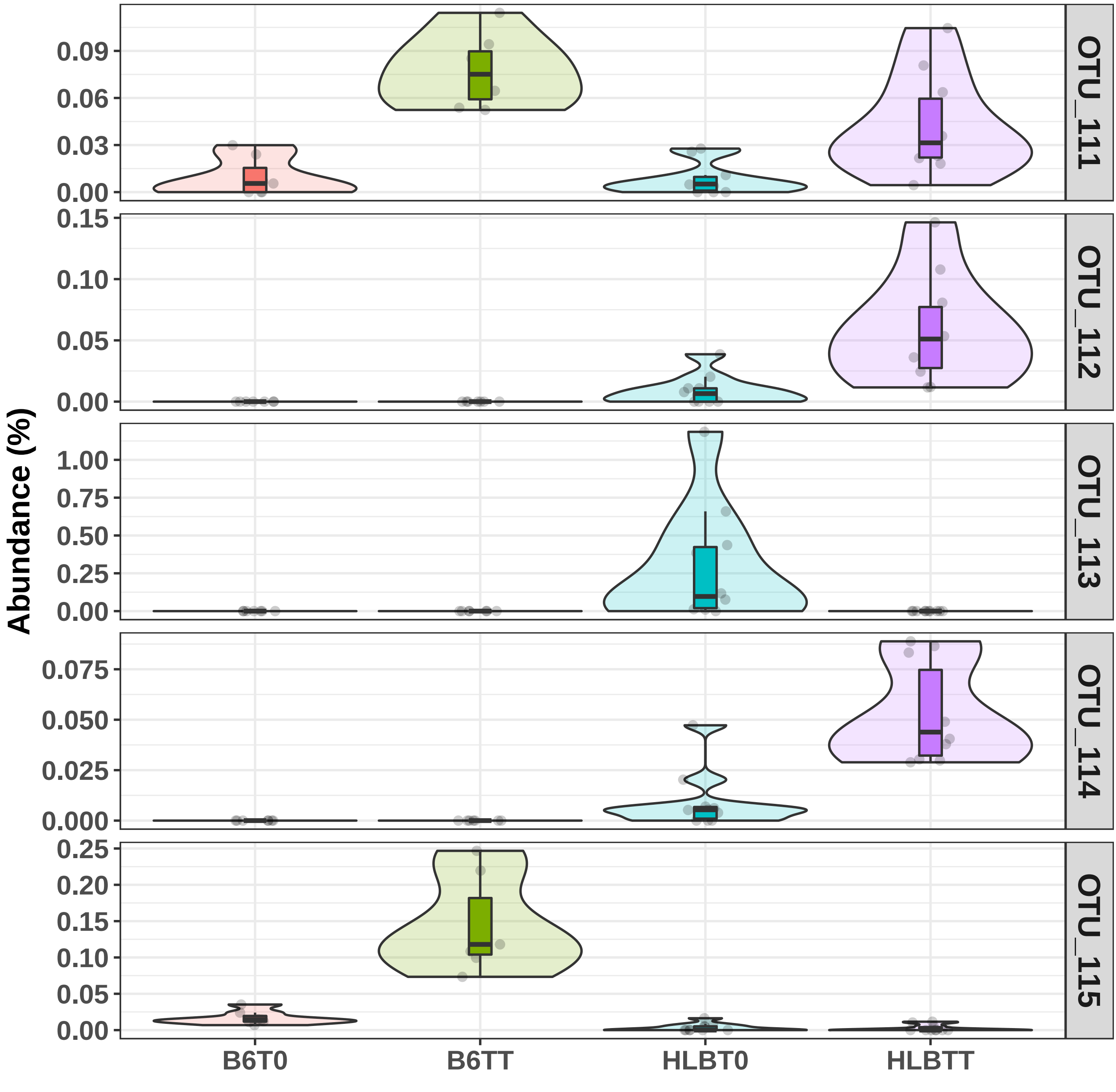
Abundance (%)
OTU_111
OTU_112
OTU_113
OTU_114
OTU_115
0.00
0.03
0.06
0.09
0.00
0.05
0.10
0.15
0.00
0.25
0.50
0.75
1.00
0.000
0.025
0.050
0.075
0.00
0.05
0.10
0.15
0.20
0.25
B6T0
B6TT
HLBT0
HLBTT

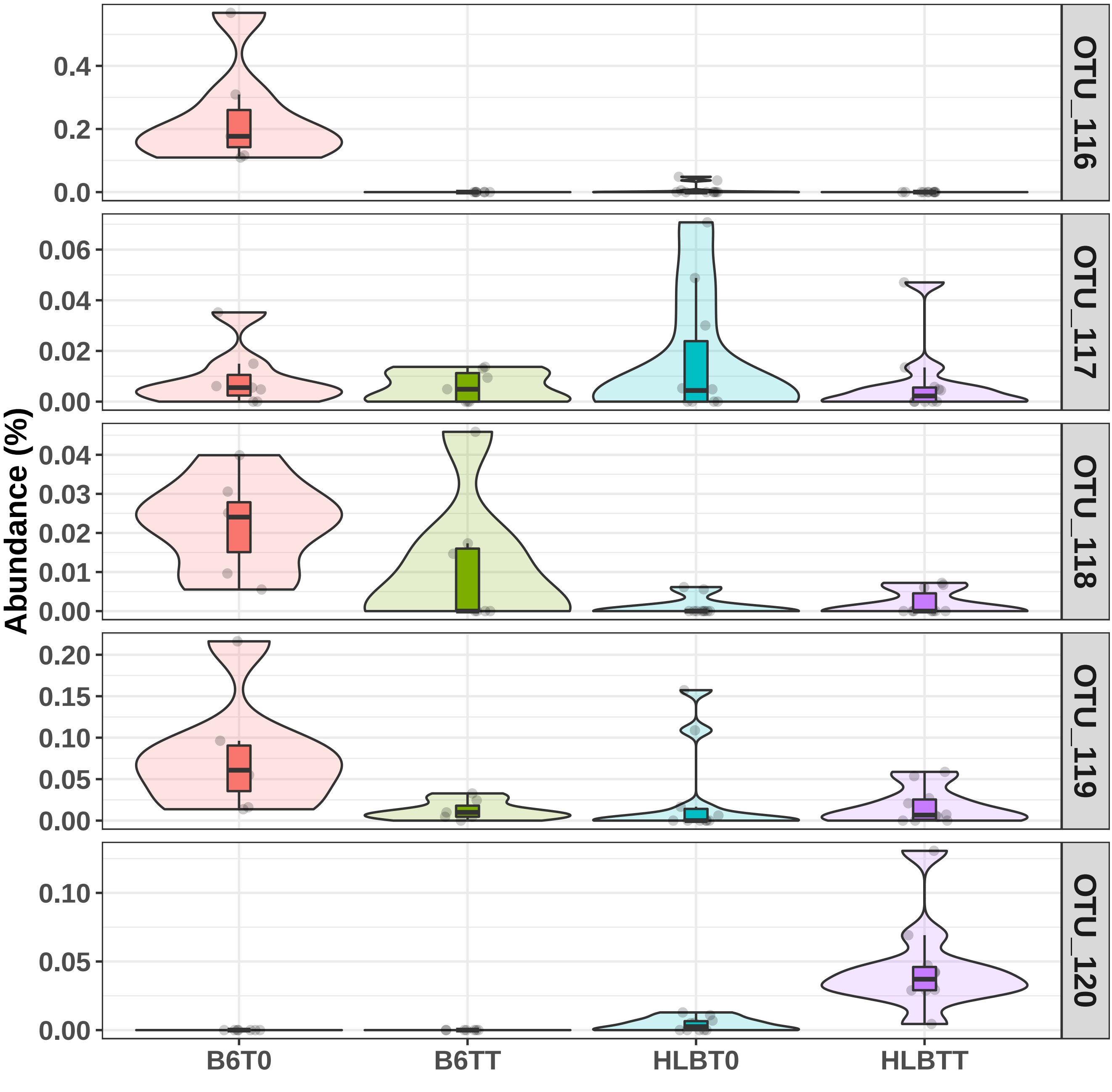

Abundance (%)
OTU_116
OTU_117
OTU_118
OTU_119
OTU_120
0.0
0.2
0.4
0.00
0.02
0.04
0.06
0.00
0.01
0.02
0.03
0.04
0.00
0.05
0.10
0.15
0.20
0.00
0.05
0.10
B6T0
B6TT
HLBT0
HLBTT

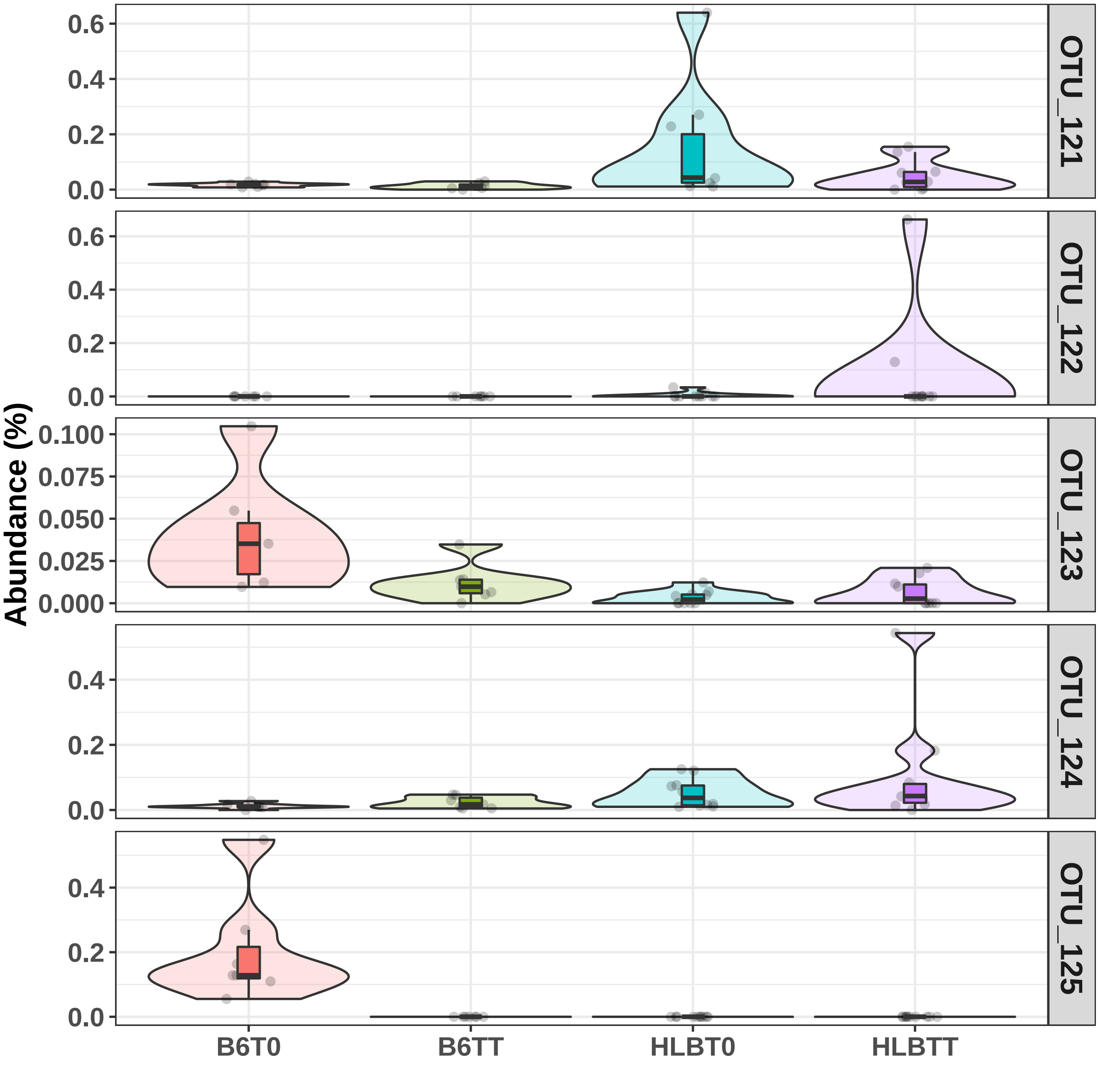

Abundance (%)
OTU_121
OTU_122
OTU_123
OTU_124
OTU_125
0.6
0.4
0.2
0.0
0.100
0.075
0.050
0.025
0.000
B6T0
B6TT
HLBT0
HLBTT

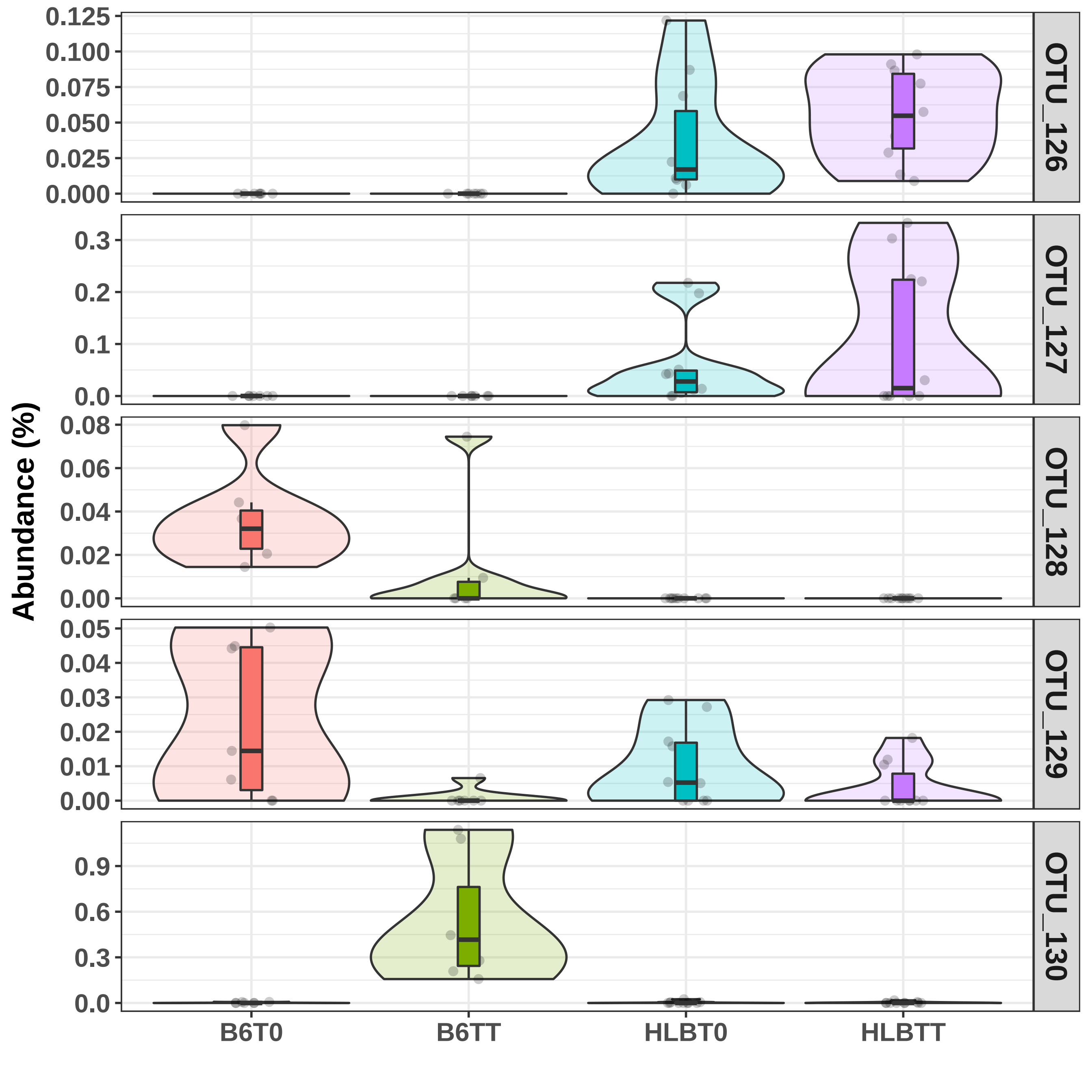

Abundance (%)
OTU_126
OTU_127
OTU_128
OTU_129
OTU_130
0.125
0.100
0.075
0.050
0.025
0.000
0.3
0.2
0.1
0.0
0.08
0.06
0.04
0.02
0.00
0.05
0.04
0.03
0.02
0.01
0.00
0.9
0.6
0.3
0.0
B6T0
B6TT
HLBT0
HLBTT

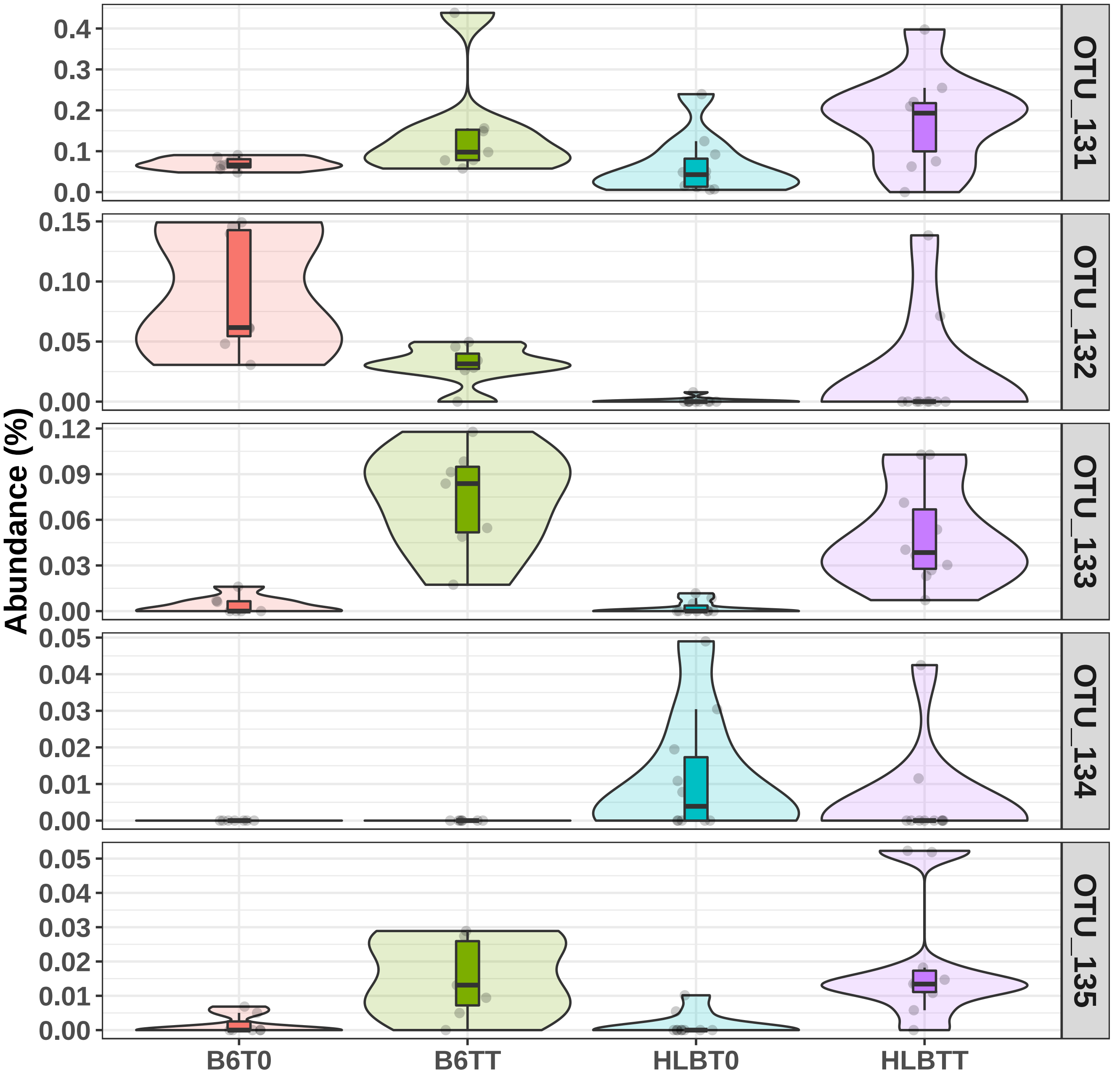

Abundance (%)
OTU_131
OTU_132
OTU_133
OTU_134
OTU_135
0.0
0.1
0.2
0.3
0.4
0.00
0.05
0.10
0.15
0.00
0.03
0.06
0.09
0.12
0.00
0.01
0.02
0.03
0.04
0.05
0.00
0.01
0.02
0.03
0.04
0.05
B6T0
B6TT
HLBT0
HLBTT

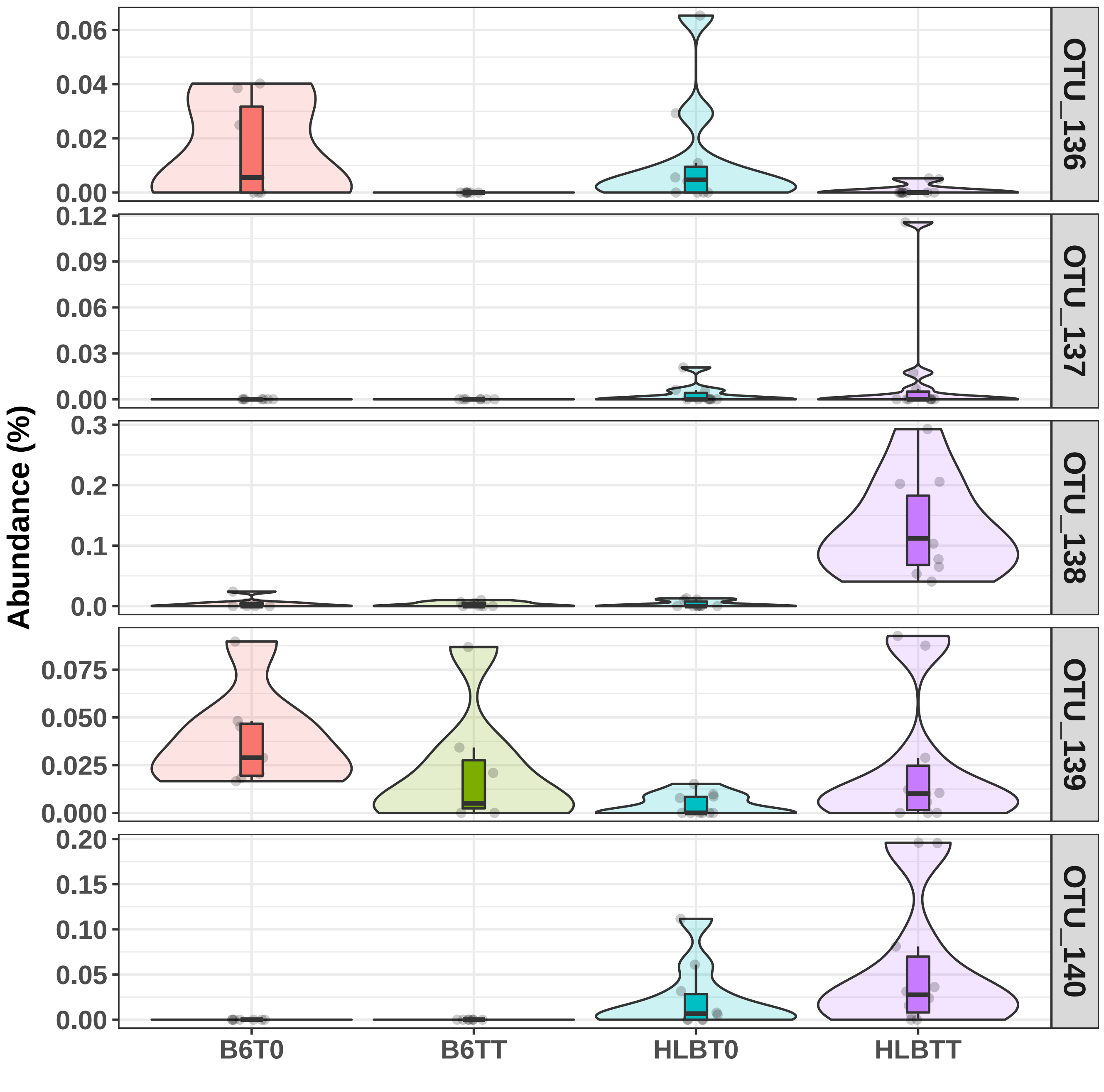

Abundance (%)
OTU_136
OTU_137
OTU_138
OTU_139
OTU_140
0.00
0.02
0.04
0.06
0.00
0.03
0.06
0.09
0.12
0.0
0.1
0.2
0.3
0.000
0.025
0.050
0.075
0.00
0.05
0.10
0.15
0.20
B6T0
B6TT
HLBT0
HLBTT

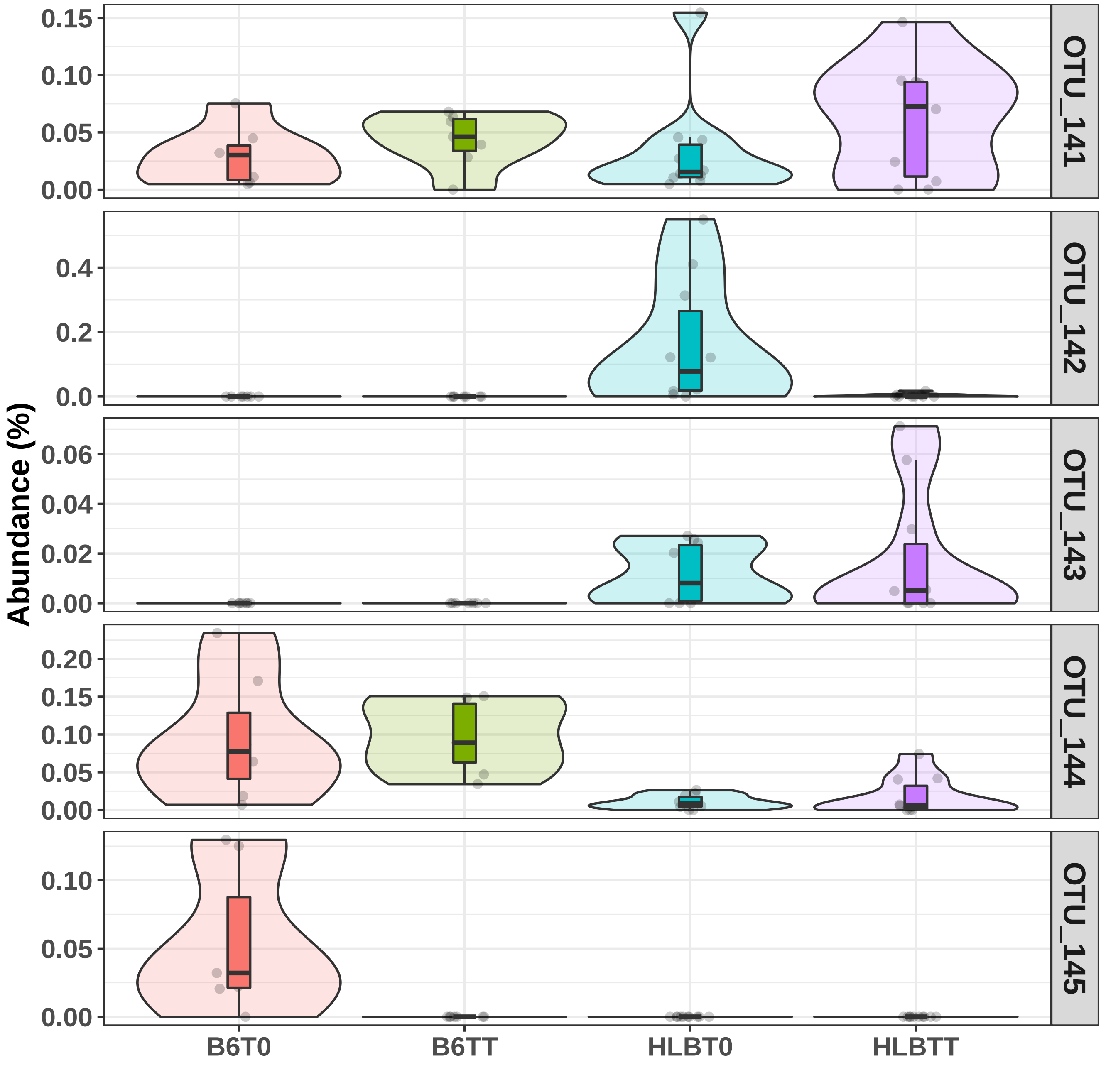

Abundance (%)
OTU_141
OTU_142
OTU_143
OTU_144
OTU_145
0.00
0.05
0.10
0.15
0.0
0.2
0.4
0.00
0.02
0.04
0.06
0.00
0.05
0.10
0.15
0.20
0.00
0.05
0.10
B6T0
B6TT
HLBT0
HLBTT

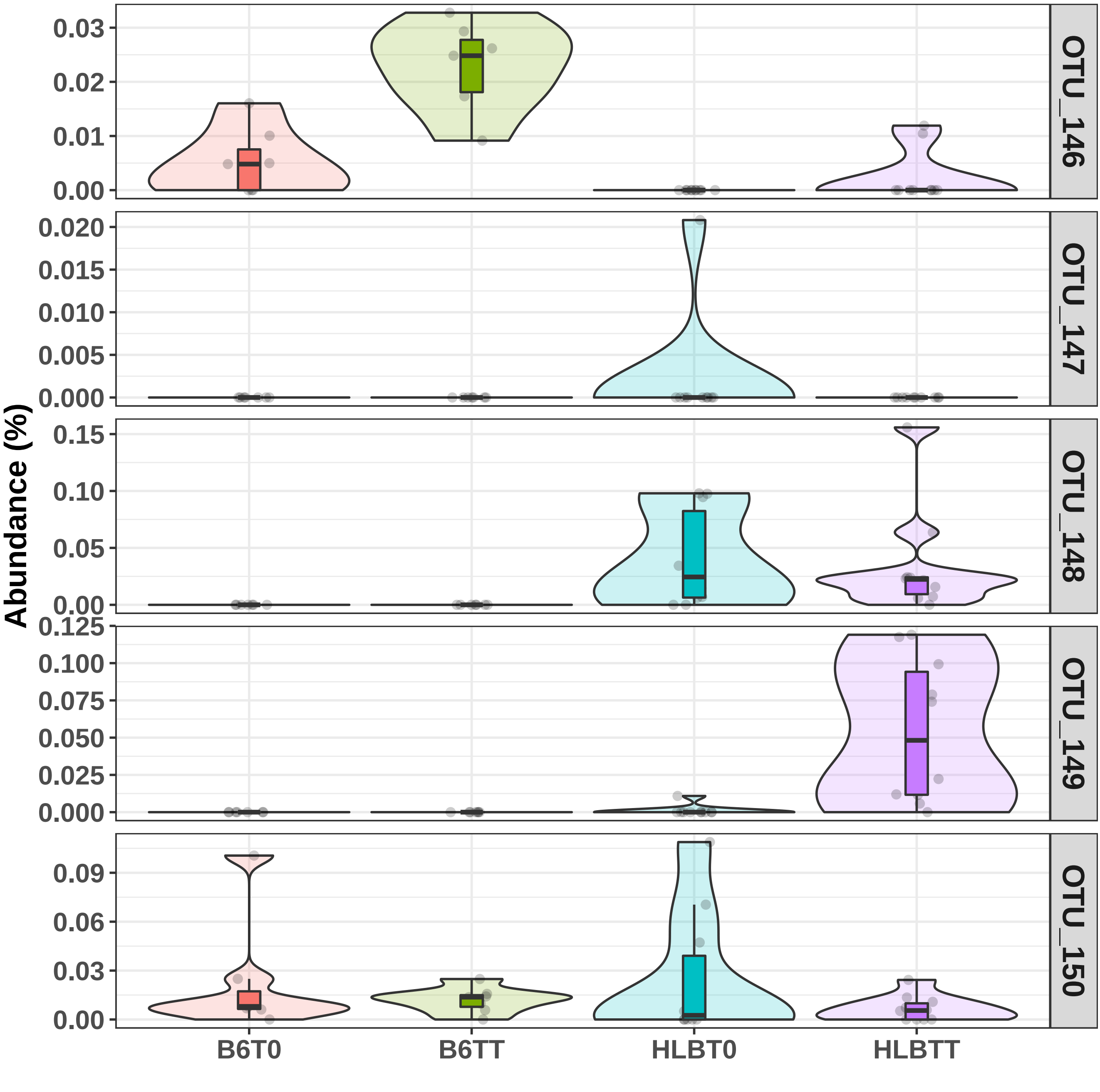
Abundance (%)
OTU_146
OTU_147
OTU_148
OTU_149
OTU_150
0.03
0.02
0.01
0.00
0.020
0.015
0.010
0.005
0.000
0.15
0.10
0.05
0.00
0.125
0.100
0.075
0.050
0.025
0.000
0.09
0.06
0.03
0.00
B6T0
B6TT
HLBT0
HLBTT

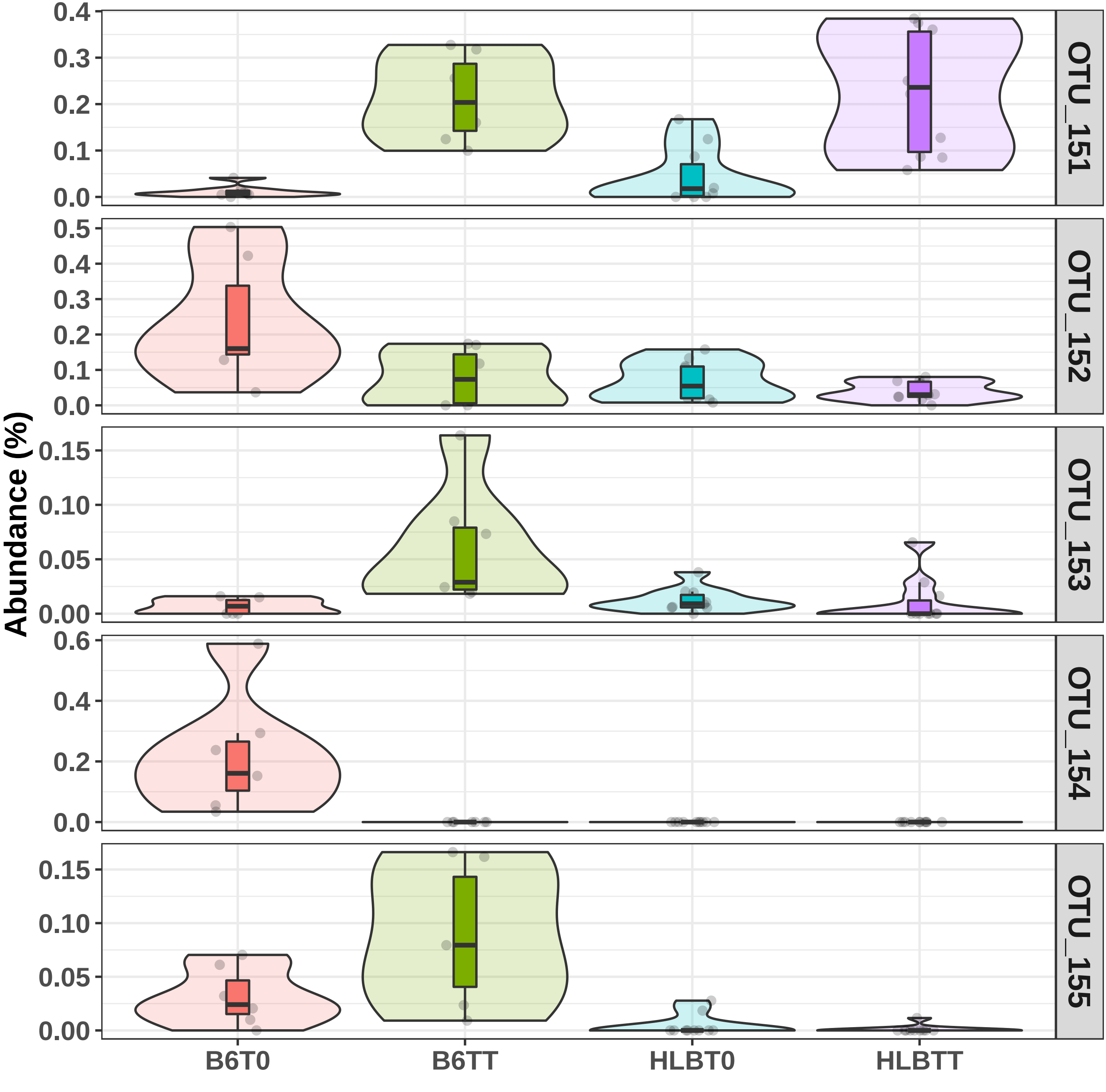

Abundance (%)
OTU_151
OTU_152
OTU_153
OTU_154
OTU_155
B6T0
B6TT
HLBT0
HLBTT

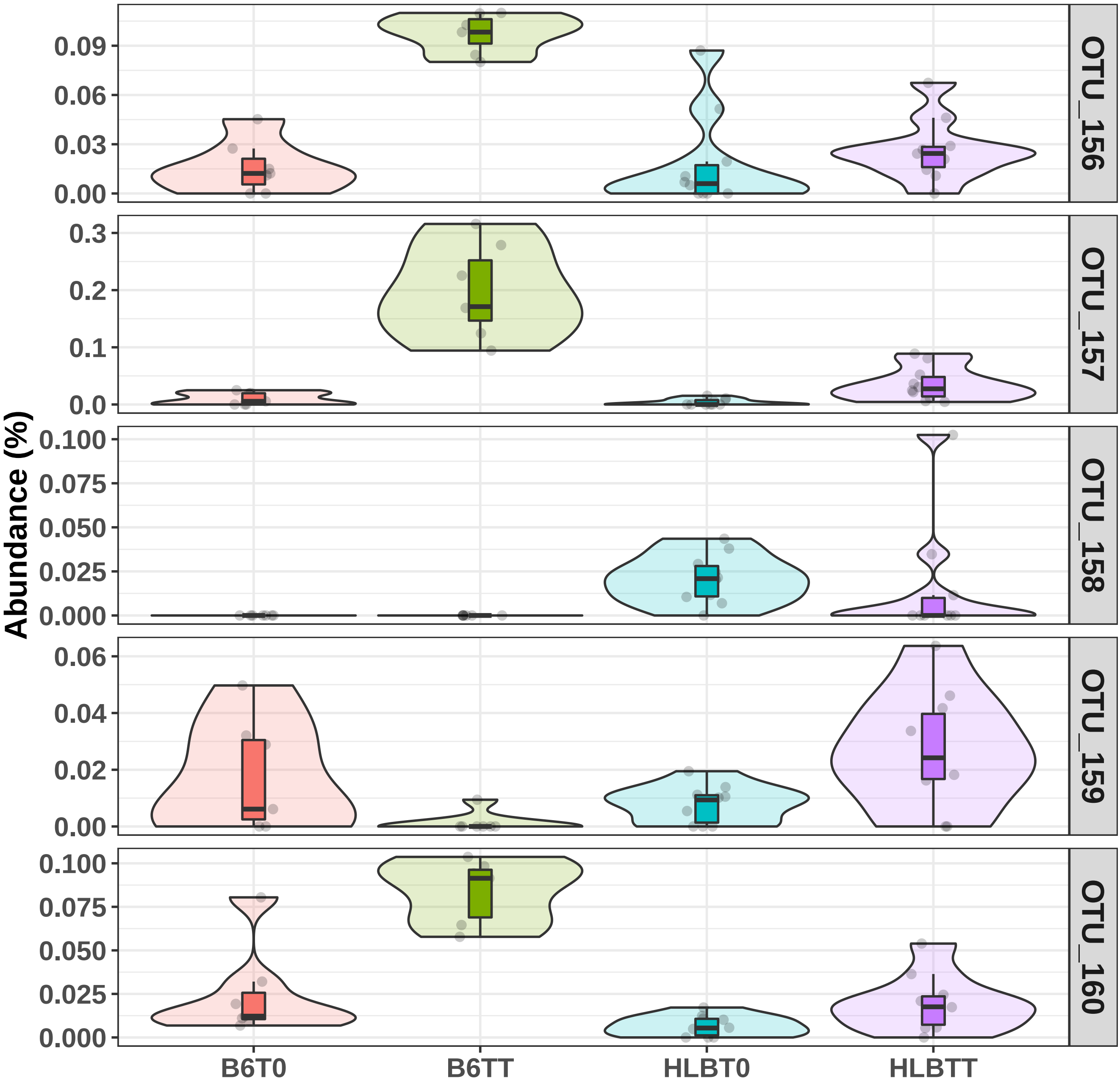

Abundance (%)
OTU_156
OTU_157
OTU_158
OTU_159
OTU_160
0.00
0.03
0.06
0.09
0.0
0.1
0.2
0.3
0.000
0.025
0.050
0.075
0.100
0.00
0.02
0.04
0.06
0.000
0.025
0.050
0.075
0.100
B6T0
B6TT
HLBT0
HLBTT

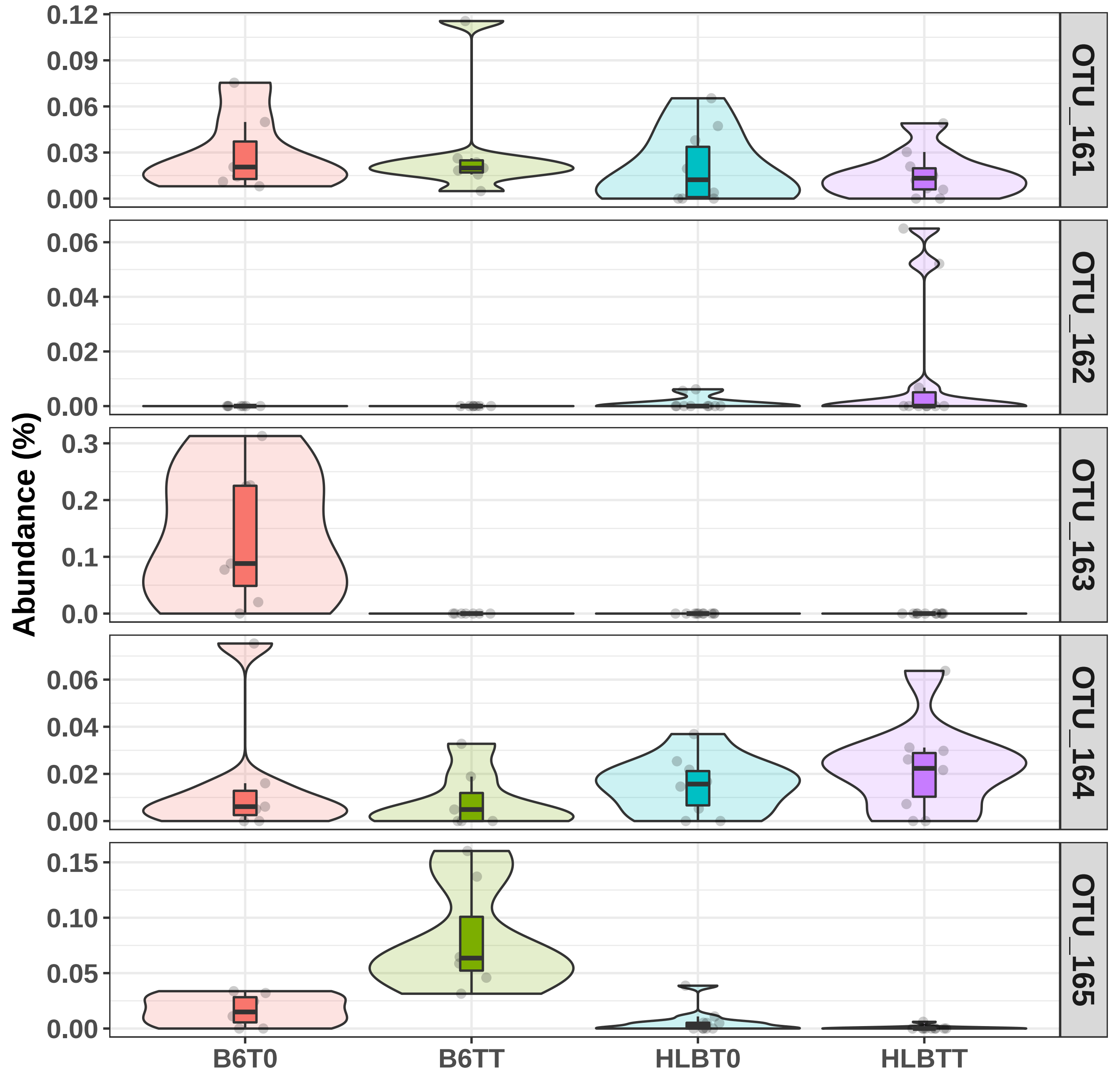
Abundance (%)
OTU_161
OTU_162
OTU_163
OTU_164
OTU_165
0.12
0.09
0.06
0.03
0.00
0.06
0.04
0.02
0.00
0.3
0.2
0.1
0.0
0.06
0.04
0.02
0.00
0.15
0.10
0.05
0.00
B6T0
B6TT
HLBT0
HLBTT

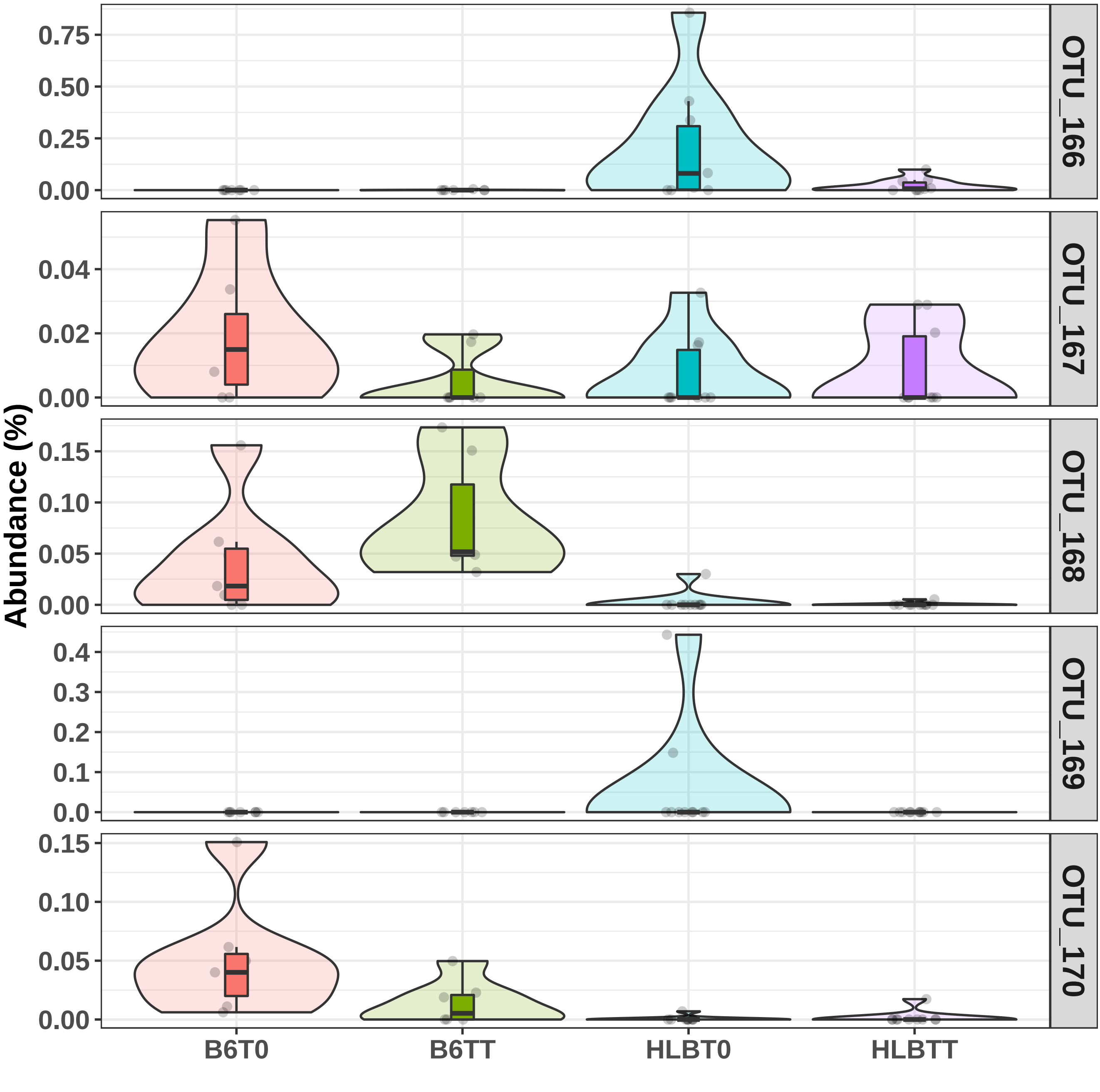

Abundance (%)
OTU_166
OTU_167
OTU_168
OTU_169
OTU_170
0.75
0.50
0.25
0.00
0.04
0.02
0.00
0.15
0.10
0.05
0.00
0.4
0.3
0.2
0.1
0.0
0.15
0.10
0.05
0.00
B6T0
B6TT
HLBT0
HLBTT

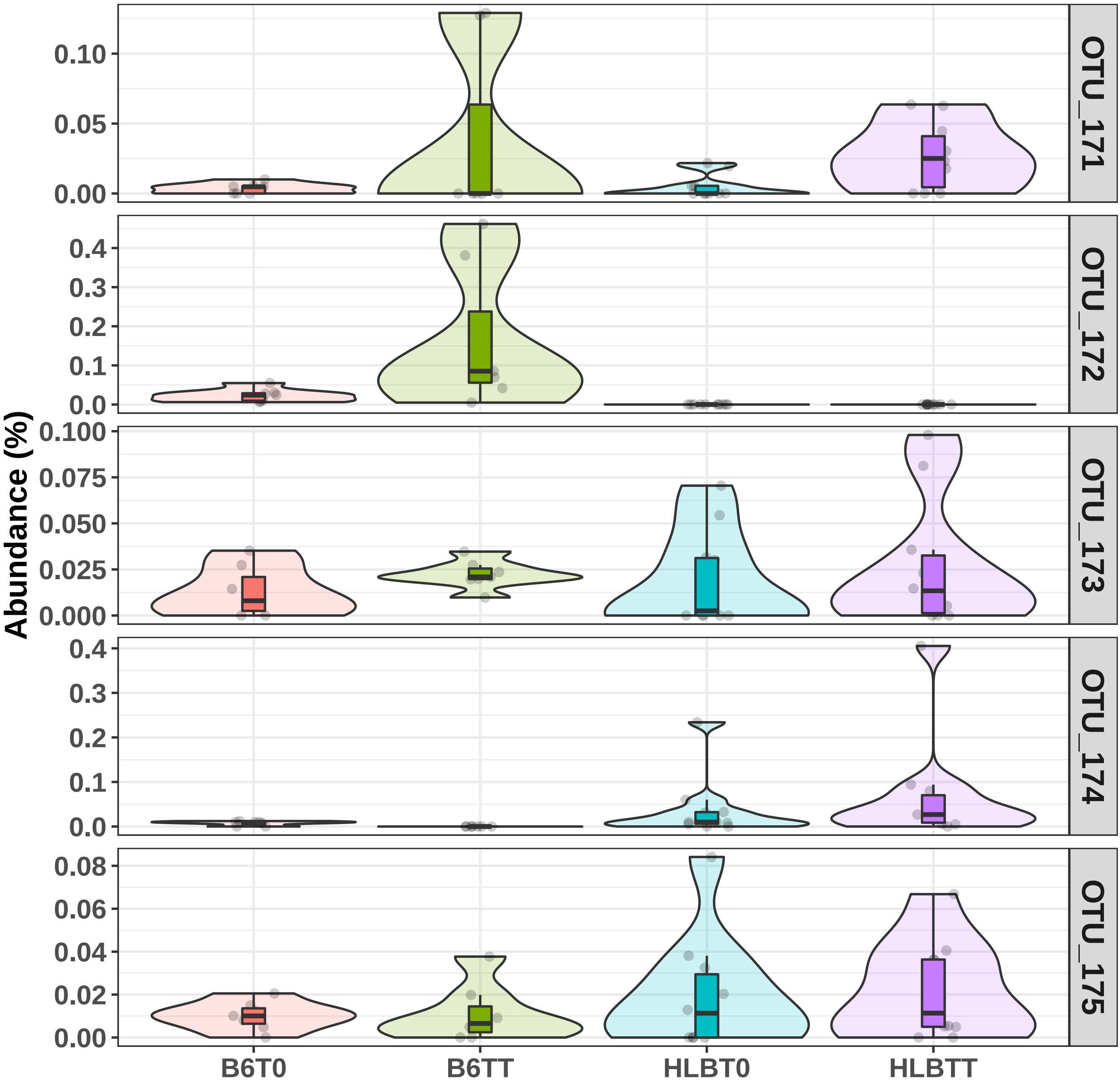

Abundance (%)
OTU_171
OTU_172
OTU_173
OTU_174
OTU_175
0.10
0.05
0.00
0.4
0.3
0.2
0.1
0.0
0.100
0.075
0.050
0.025
0.000
0.08
0.06
0.04
0.02
B6T0
B6TT
HLBT0
HLBTT

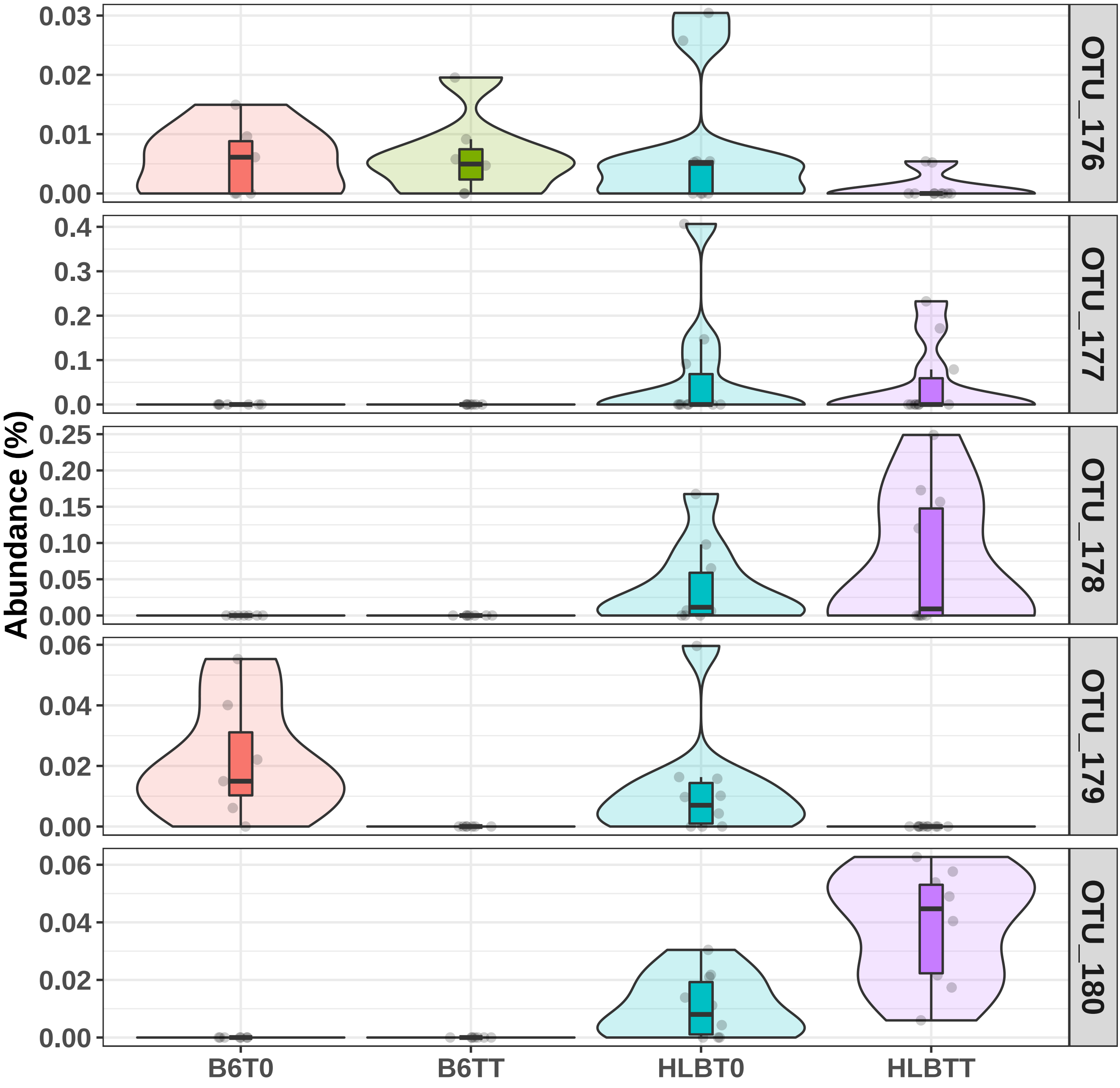

Abundance (%)
OTU_176
OTU_177
OTU_178
OTU_179
OTU_180
0.03
0.02
0.01
0.00
0.4
0.3
0.2
0.1
0.0
0.25
0.20
0.15
0.10
0.05
0.00
0.06
0.04
0.02
0.00
0.06
0.04
0.02
0.00
B6T0
B6TT
HLBT0
HLBTT

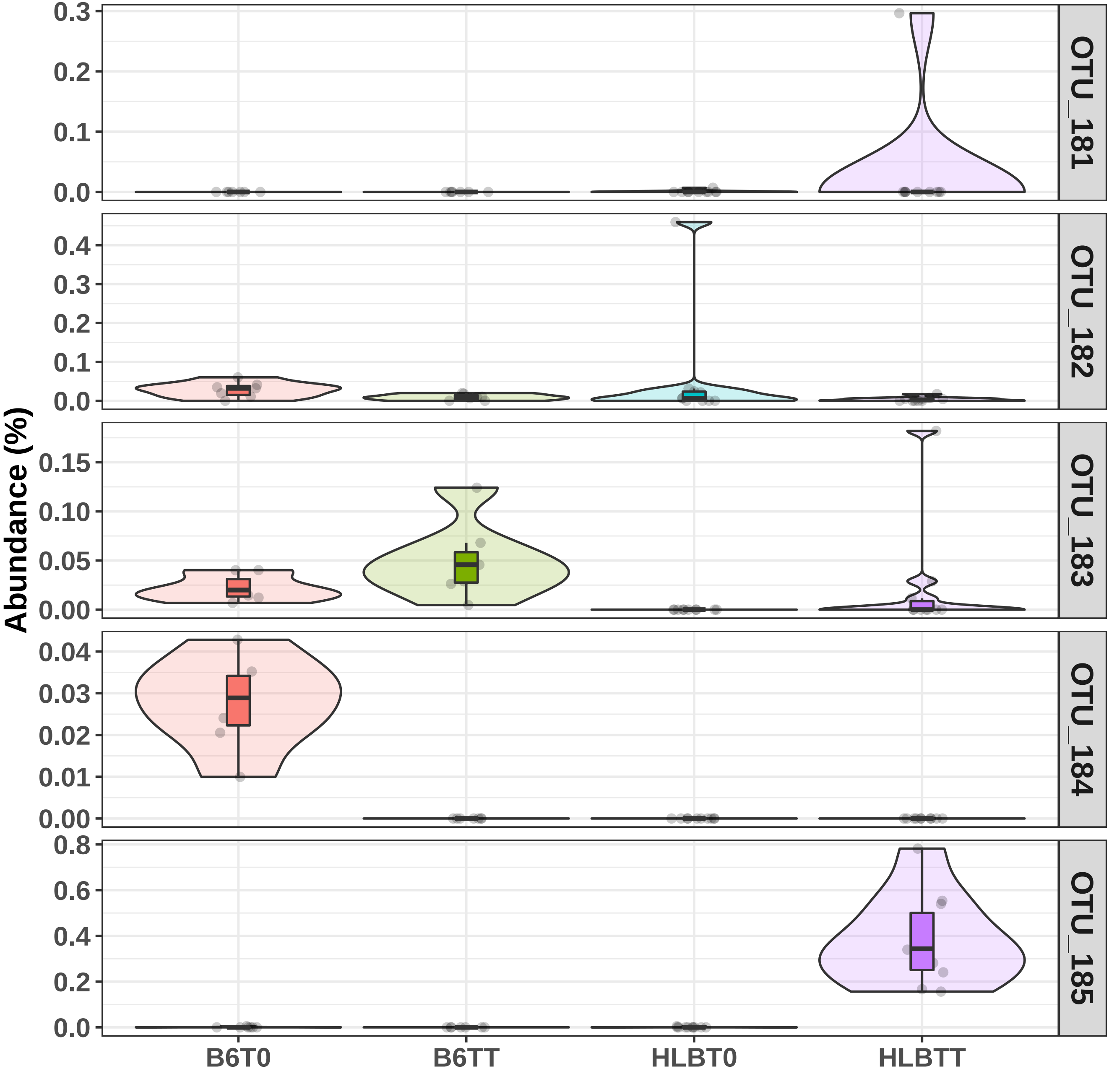
Abundance (%)
OTU_181
OTU_182
OTU_183
OTU_184
OTU_185
B6T0
B6TT
HLBT0
HLBTT

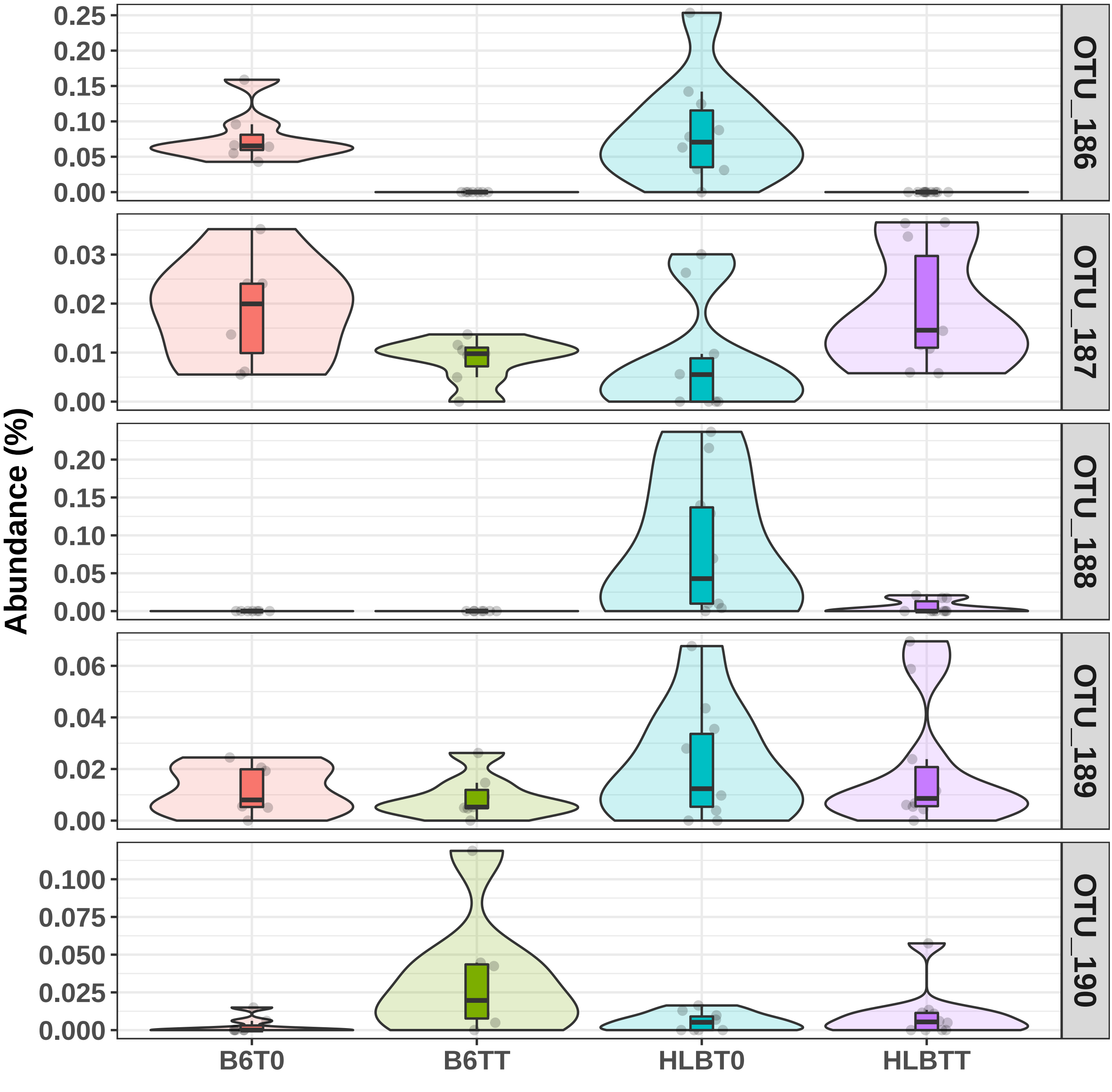
Abundance (%)
OTU_186
OTU_187
OTU_188
OTU_189
OTU_190
0.25
0.20
0.15
0.10
0.05
0.00
0.03
0.02
0.01
0.06
0.04
0.100
0.075
0.050
0.025
0.000
B6T0
B6TT
HLBT0
HLBTT

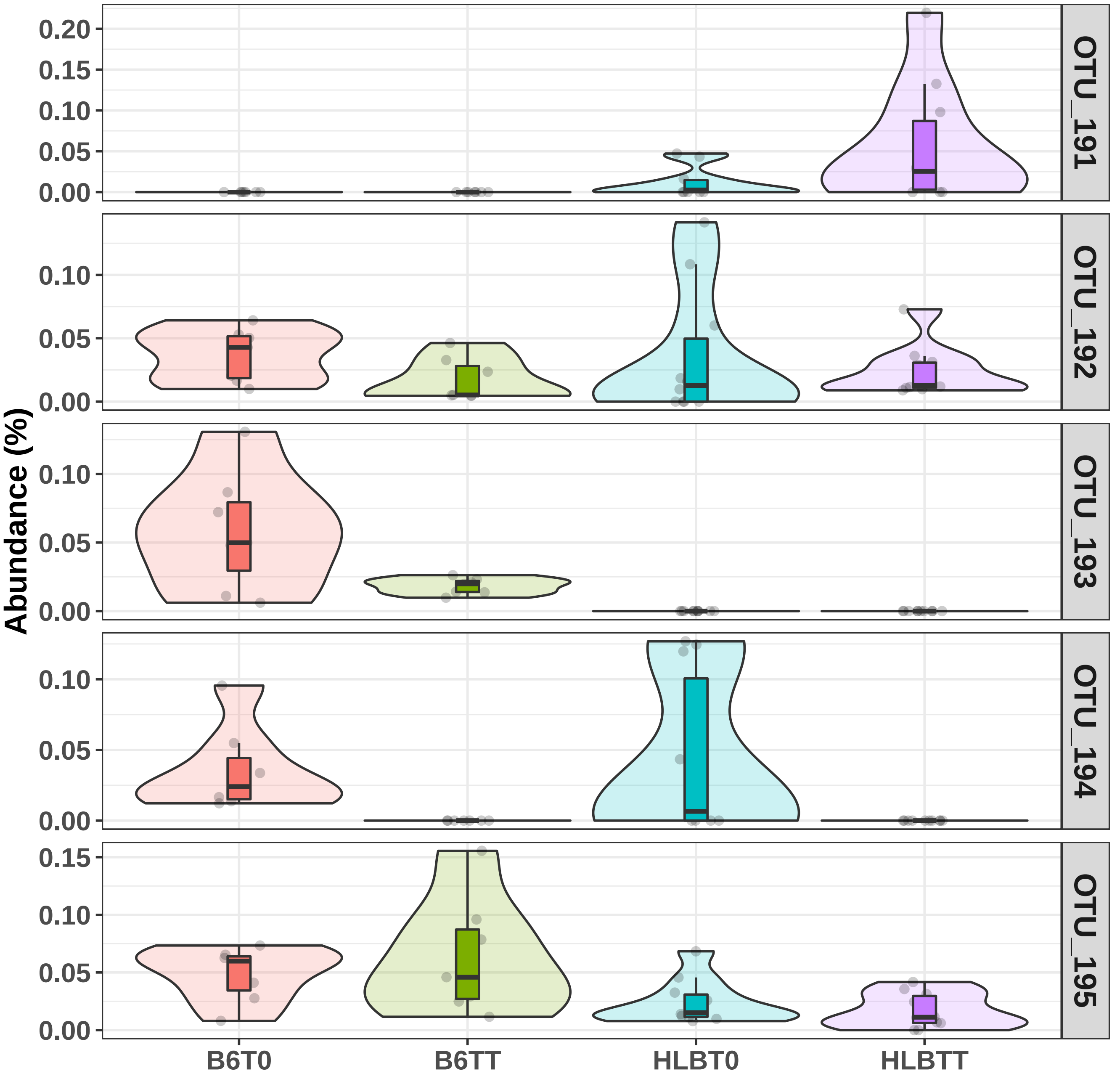

Abundance (%)
OTU_191
OTU_192
OTU_193
OTU_194
OTU_195
0.00
0.05
0.10
0.15
0.20
B6T0
B6TT
HLBT0
HLBTT

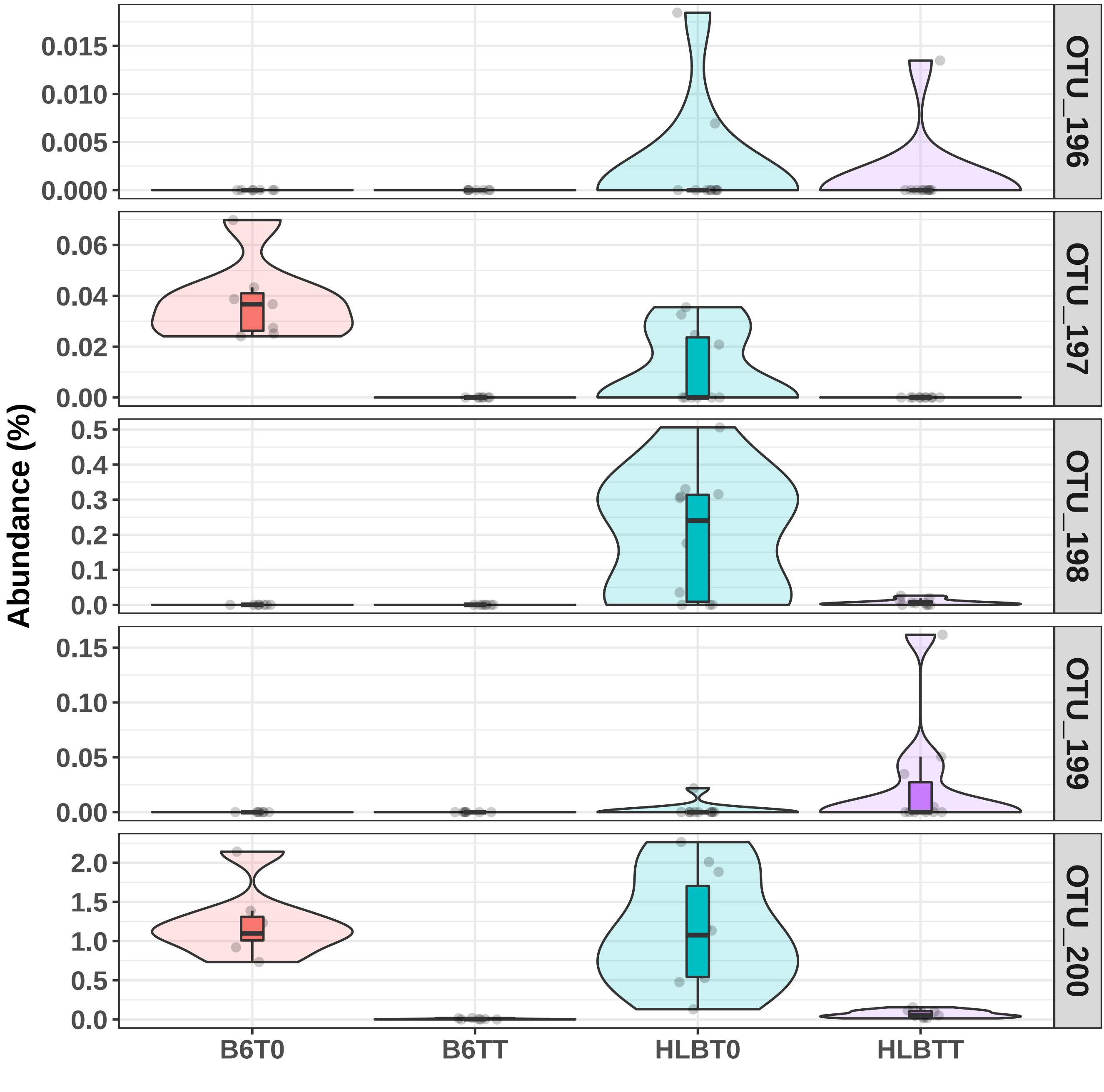
Abundance (%)
OTU_196
OTU_197
OTU_198
OTU_199
OTU_200
0.000
0.005
0.010
0.015
0.00
0.02
0.04
0.06
0.0
0.1
0.2
0.3
0.4
0.5
0.00
0.05
0.10
0.15
0.0
0.5
1.0
1.5
2.0
B6T0
B6TT
HLBT0
HLBTT

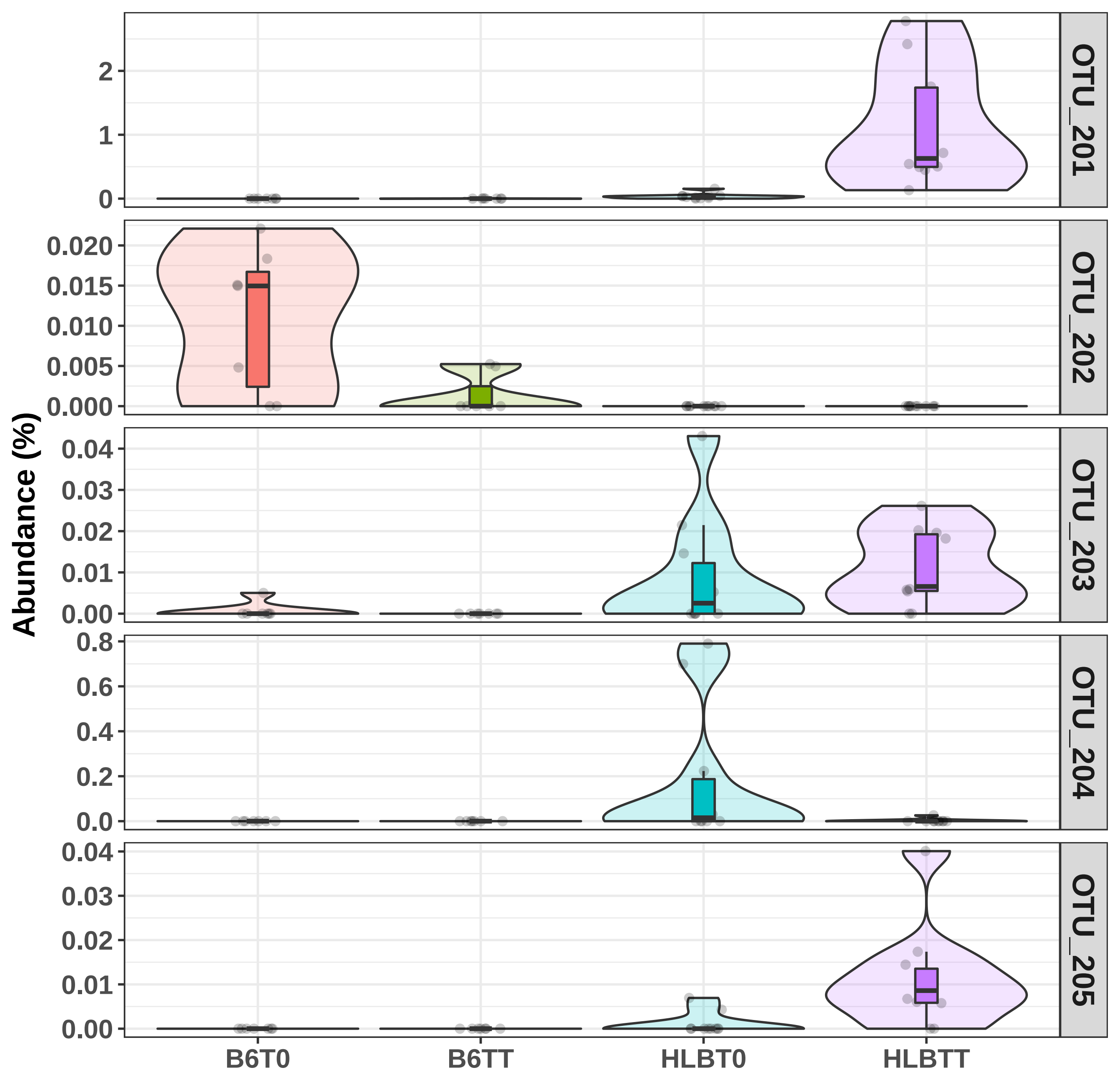
Abundance (%)
OTU_201
OTU_202
OTU_203
OTU_204
OTU_205
B6T0
B6TT
HLBT0
HLBTT

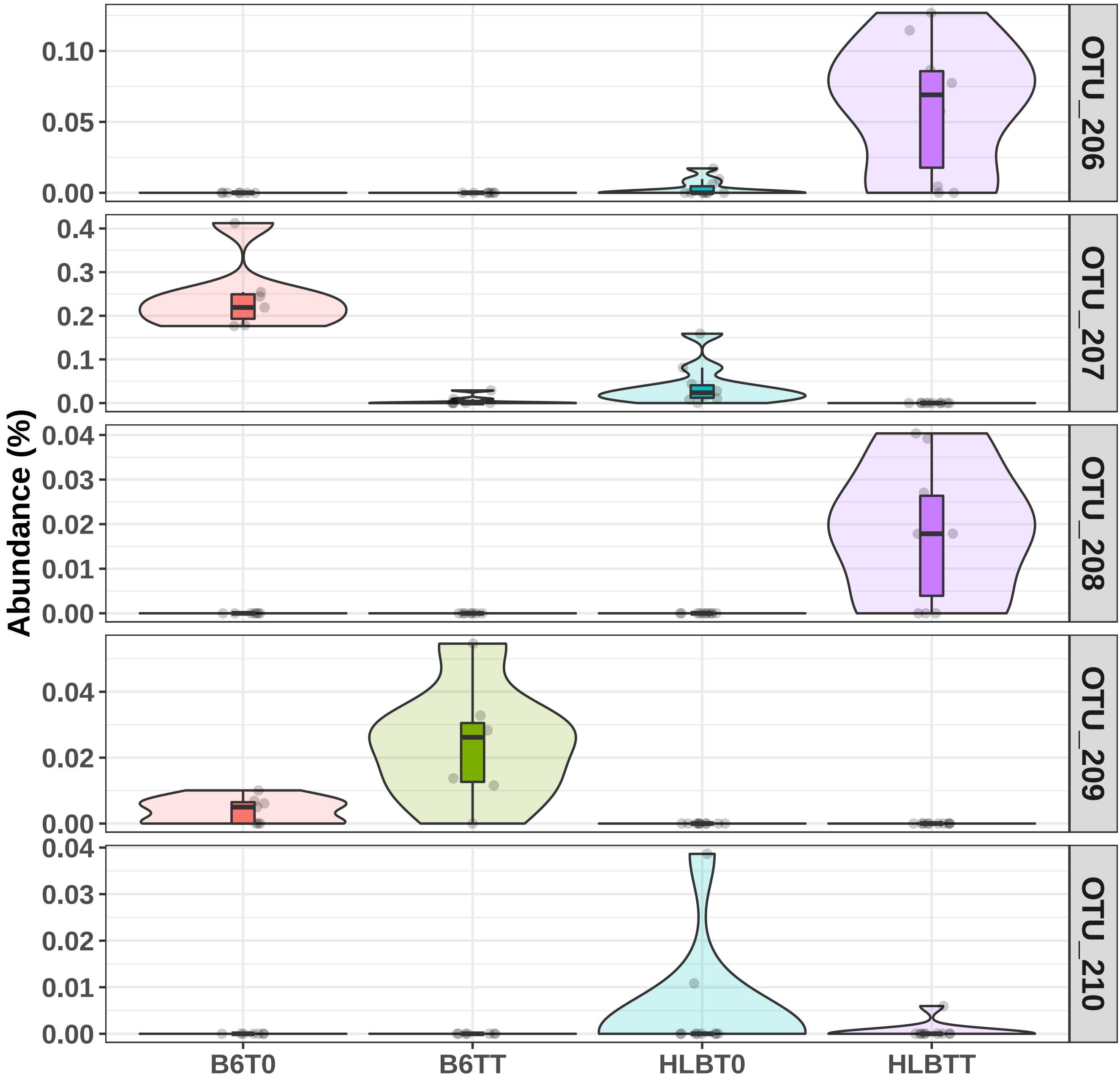

Abundance (%)
OTU_206
OTU_207
OTU_208
OTU_209
OTU_210
0.10
0.05
0.00
0.4
0.3
0.2
0.1
0.0
0.04
0.03
0.02
0.01
0.00
0.04
0.02
0.00
0.04
0.03
0.02
0.01
0.00
B6T0
B6TT
HLBT0
HLBTT

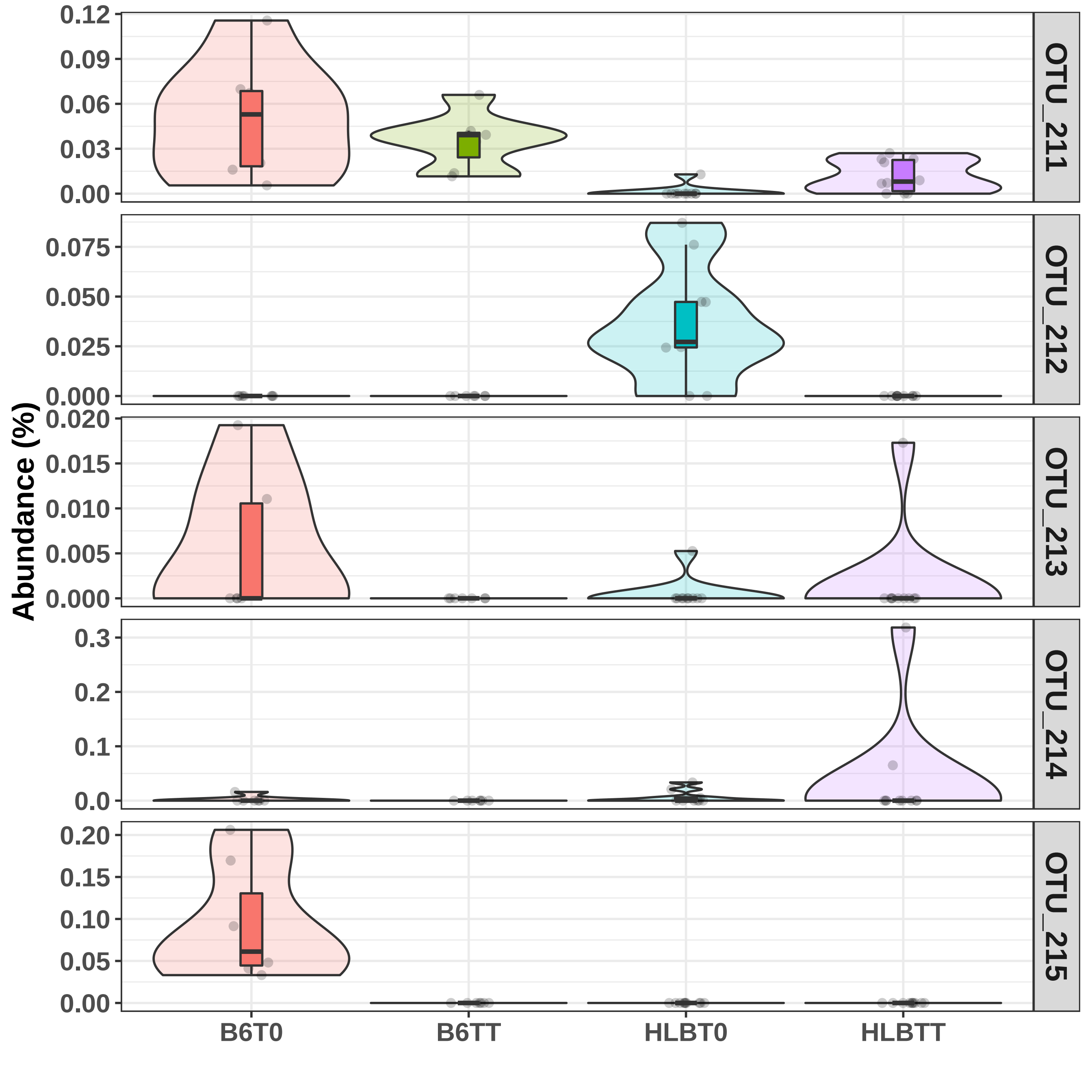

Abundance (%)
OTU_211
OTU_212
OTU_213
OTU_214
OTU_215
0.12
0.09
0.06
0.03
0.00
0.075
0.050
0.025
0.000
0.020
0.015
0.010
0.005
0.000
0.3
0.2
0.1
0.0
0.20
0.15
0.10
0.05
0.00
B6T0
B6TT
HLBT0
HLBTT

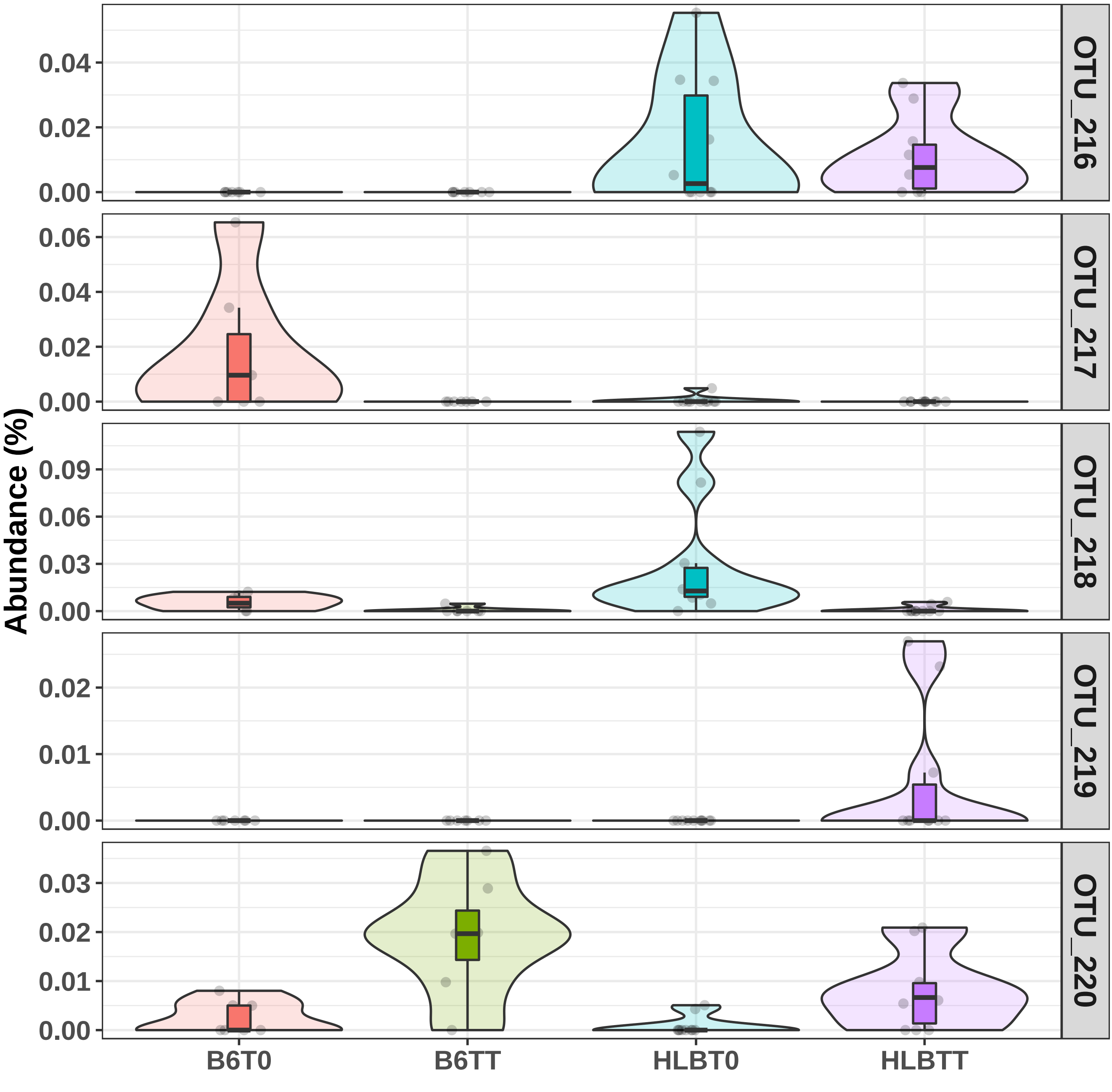

Abundance (%)
OTU_216
OTU_217
OTU_218
OTU_219
OTU_220
0.00
0.02
0.04
0.06
0.03
0.09
0.01
B6T0
B6TT
HLBT0
HLBTT

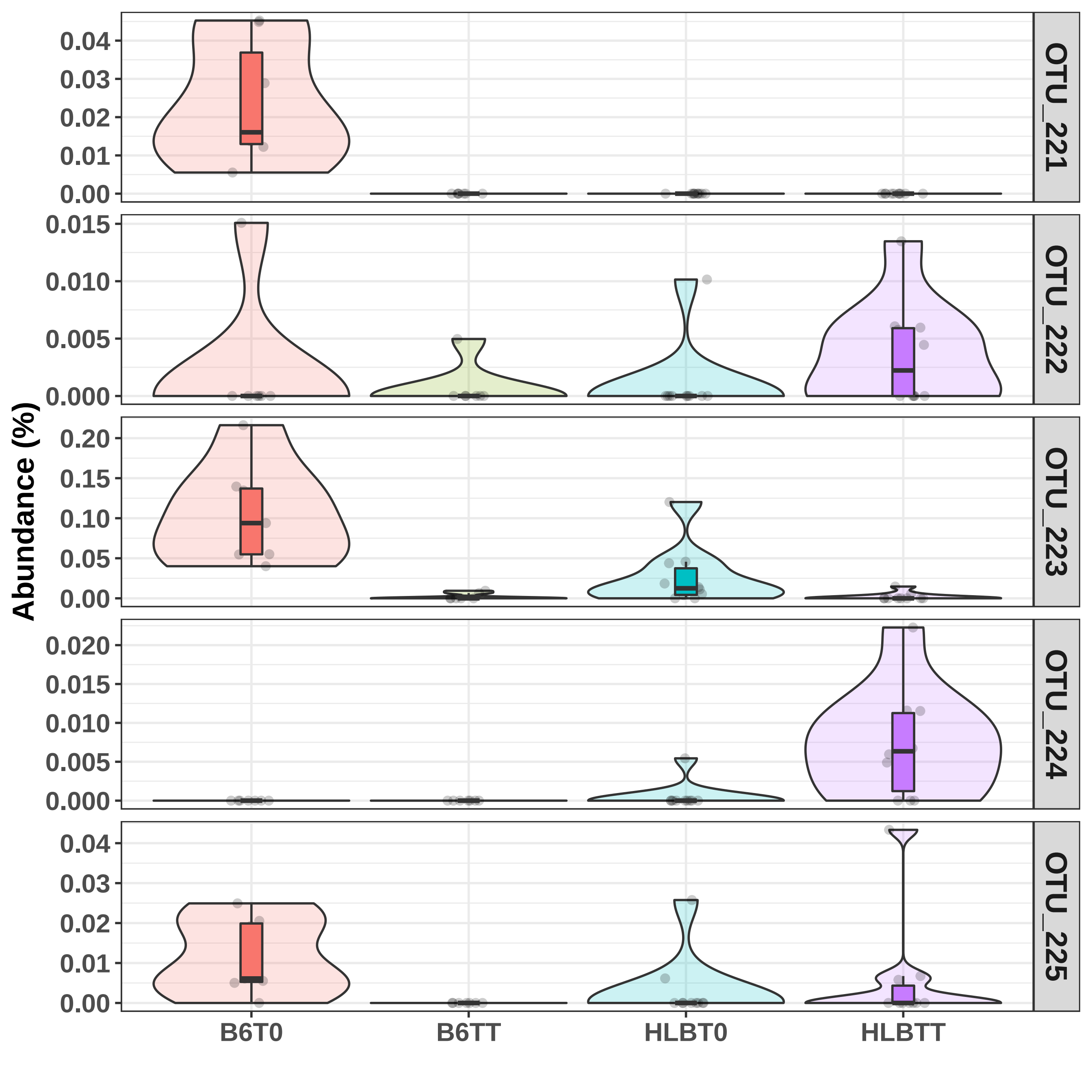

Abundance (%)
OTU_221
OTU_222
OTU_223
OTU_224
OTU_225
0.04
0.03
0.02
0.01
0.00
0.015
0.010
0.005
0.000
0.20
0.15
0.10
0.05
0.00
0.020
0.015
0.010
0.005
0.000
0.04
0.03
0.02
0.01
0.00
B6T0
B6TT
HLBT0
HLBTT

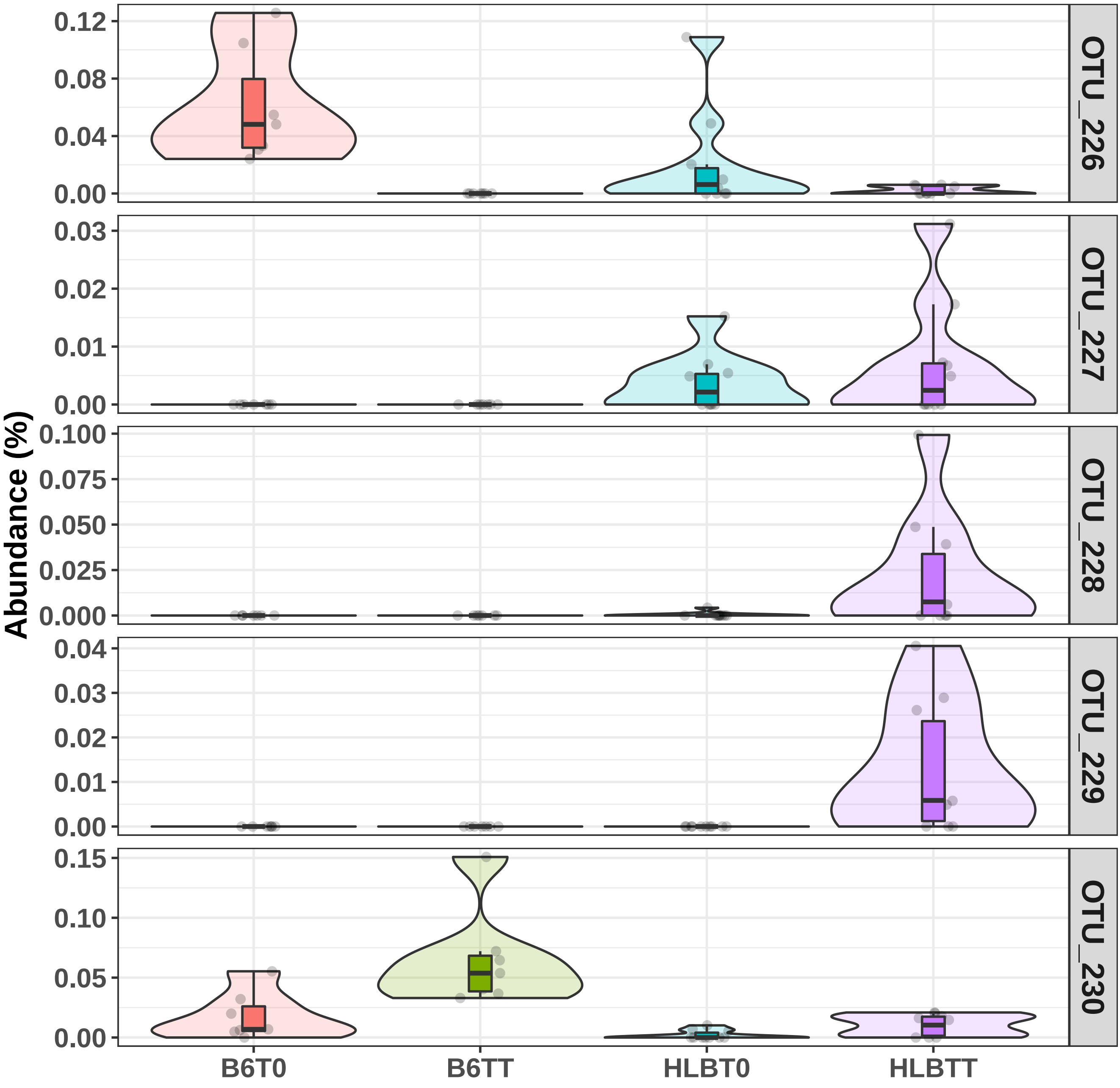

Abundance (%)
OTU_226
OTU_227
OTU_228
OTU_229
OTU_230
0.12
0.08
0.04
0.00
0.03
0.02
0.01
0.100
0.075
0.050
0.025
0.000
0.04
0.15
0.10
0.05
B6T0
B6TT
HLBT0
HLBTT

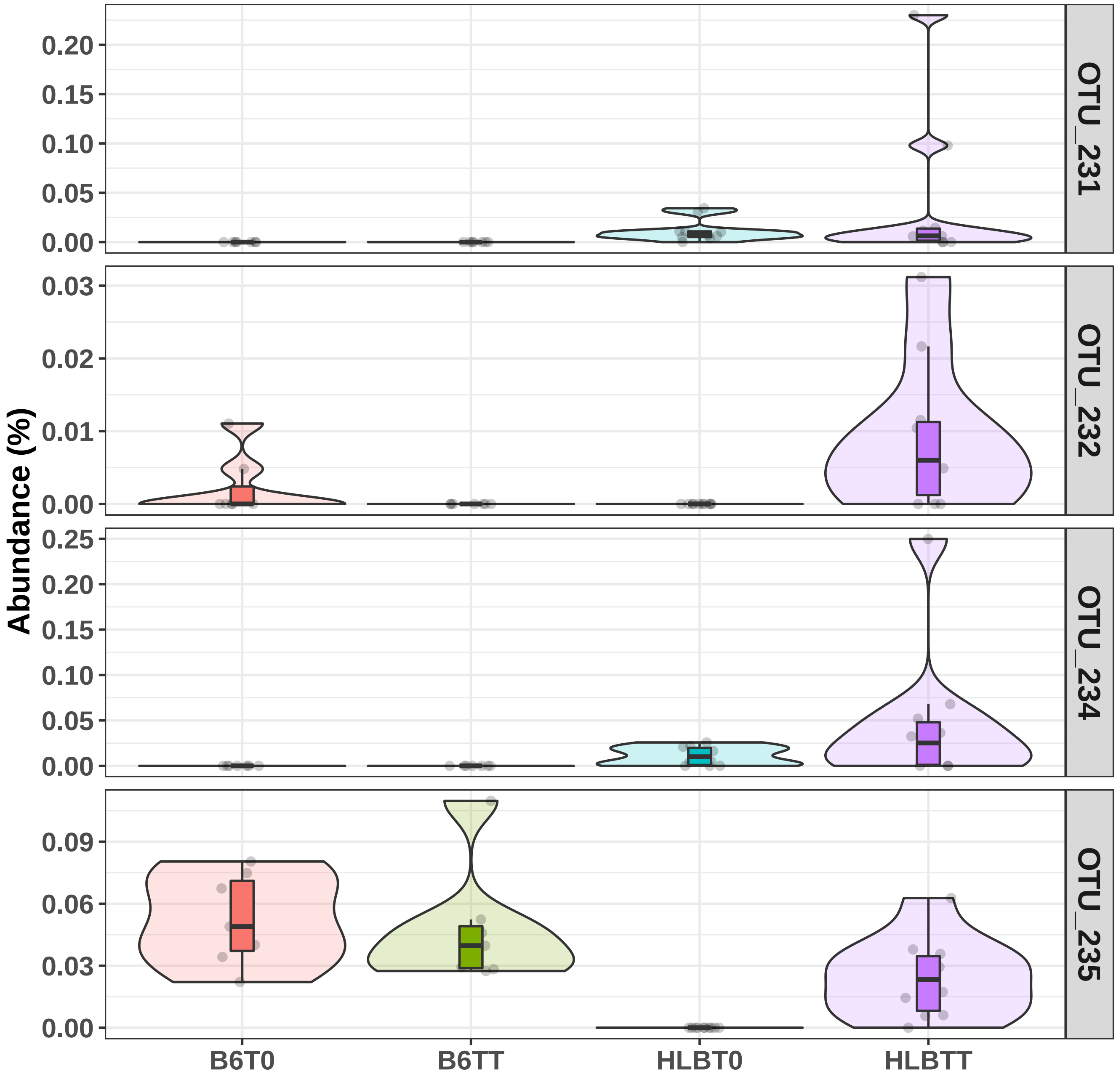

Abundance (%)
OTU_231
OTU_232
OTU_234
OTU_235
0.00
0.05
0.10
0.15
0.20
0.01
0.02
0.03
0.25
0.03
0.06
0.09
B6T0
B6TT
HLBT0
HLBTT

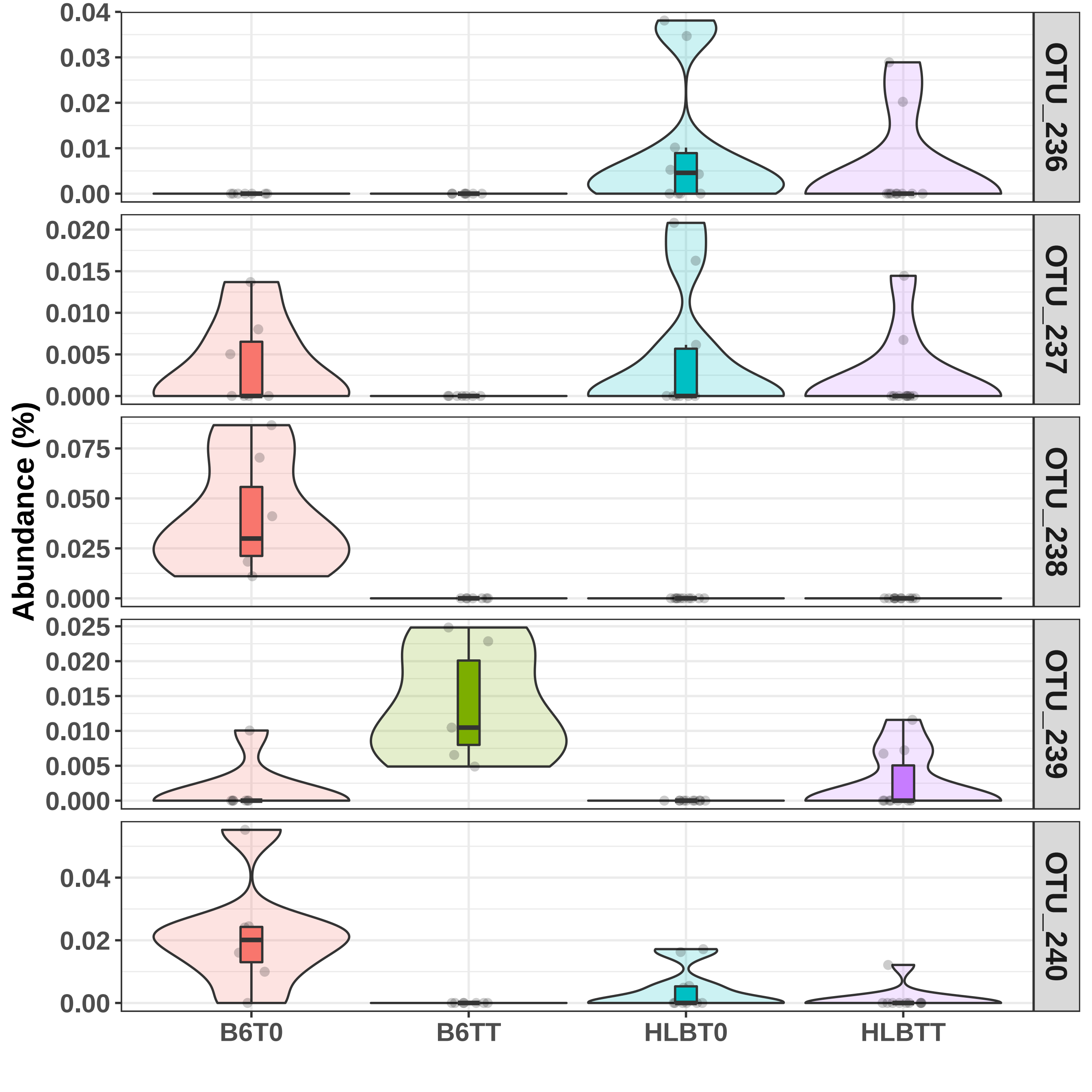
Abundance (%)
OTU_236
OTU_237
OTU_238
OTU_239
OTU_240
B6T0
B6TT
HLBT0
HLBTT

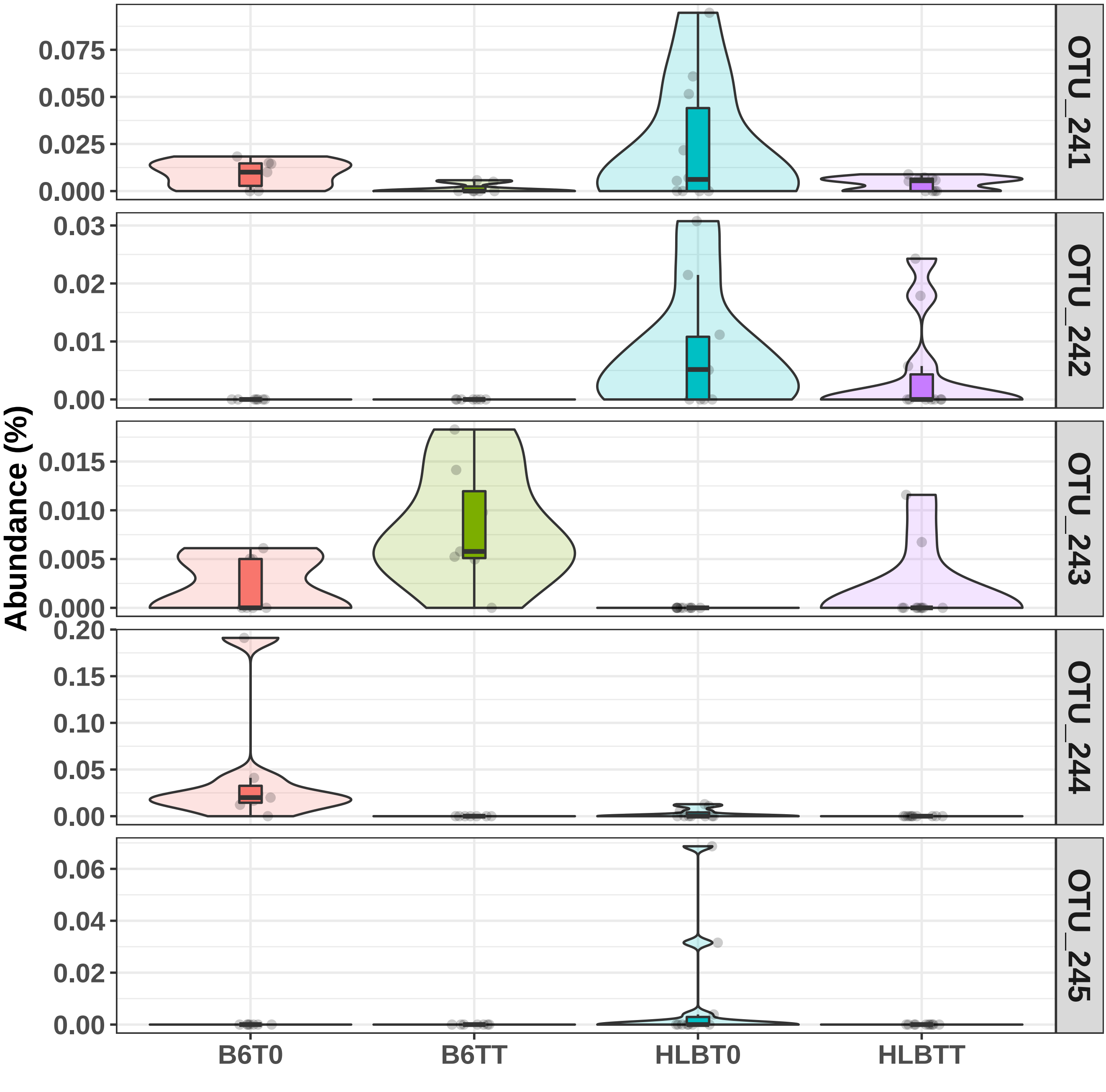
Abundance (%)
OTU_241
OTU_242
OTU_243
OTU_244
OTU_245
0.075
0.050
0.025
0.000
0.03
0.02
0.01
0.00
0.015
0.010
0.005
0.20
0.15
0.10
0.05
0.06
0.04
B6T0
B6TT
HLBT0
HLBTT

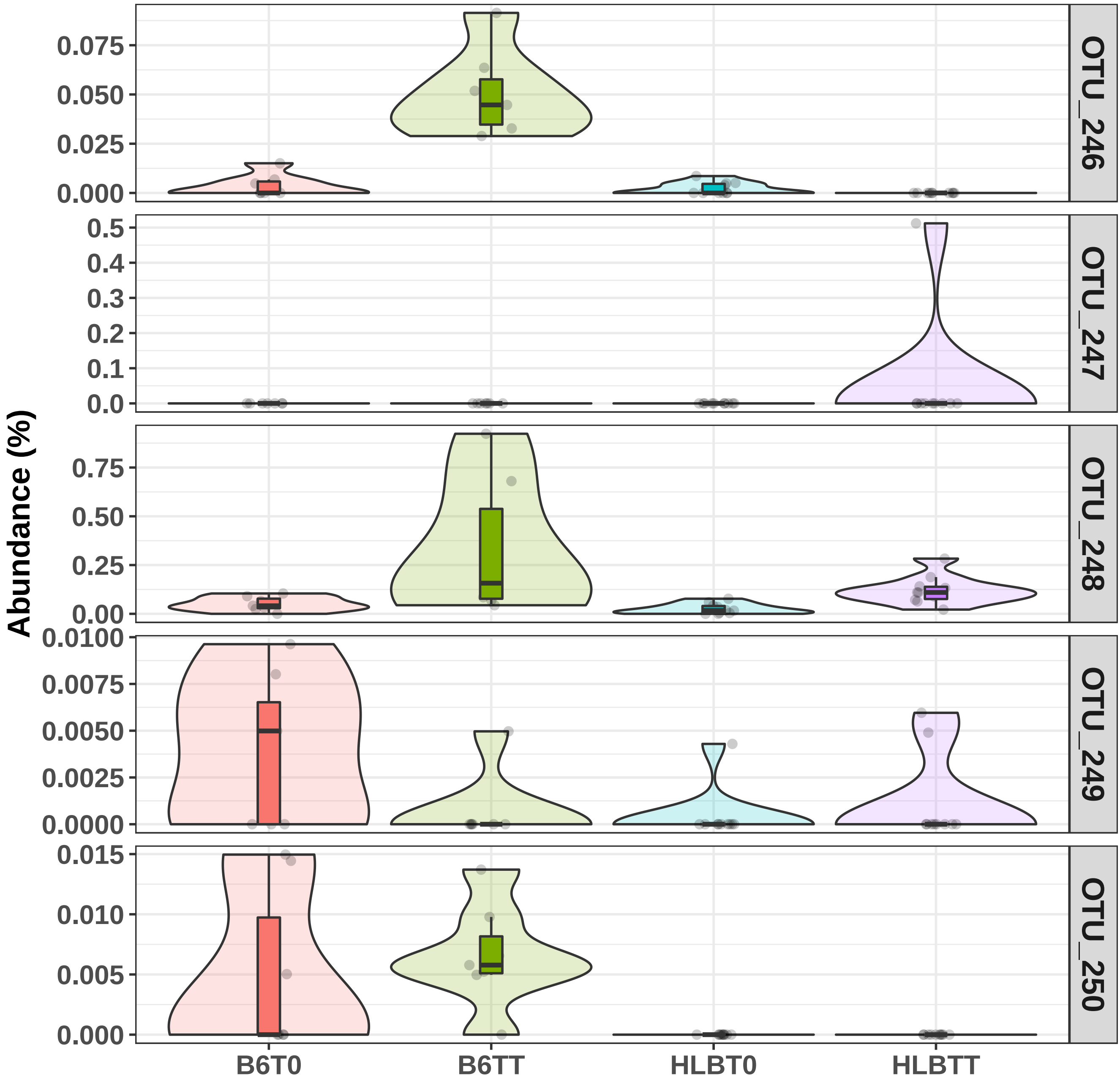

Abundance (%)
OTU_246
OTU_247
OTU_248
OTU_249
OTU_250
0.075
0.050
0.025
0.000
0.5
0.4
0.3
0.2
0.1
0.0
0.75
0.50
0.25
0.00
0.0100
0.0075
0.0050
0.0025
0.0000
0.015
0.010
0.005
0.000
B6T0
B6TT
HLBT0
HLBTT

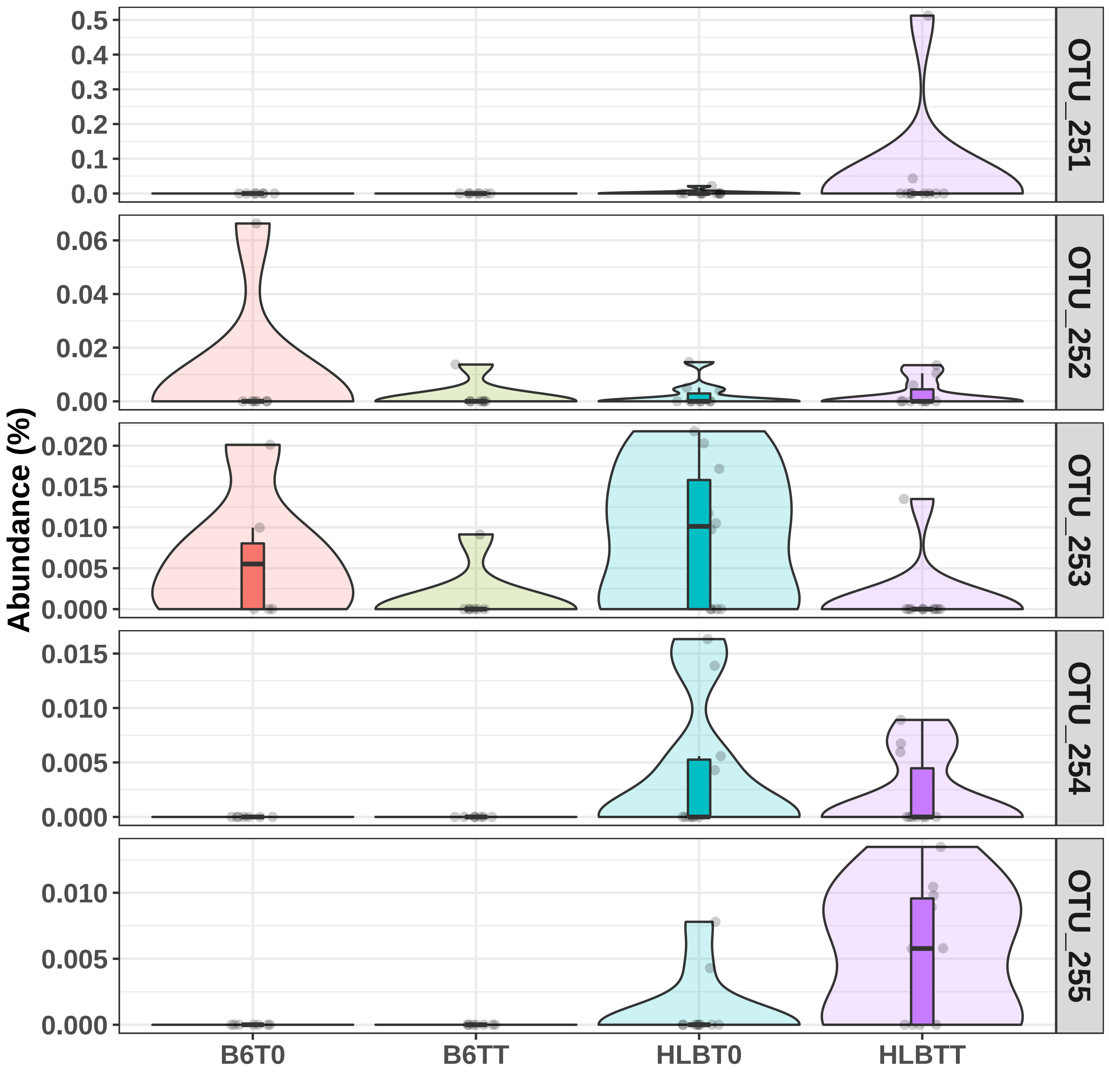

Abundance (%)
OTU_251
OTU_252
OTU_253
OTU_254
OTU_255
0.5
0.4
0.3
0.2
0.1
0.0
0.06
0.04
0.02
0.00
0.020
0.015
0.010
0.005
0.000
B6T0
B6TT
HLBT0
HLBTT

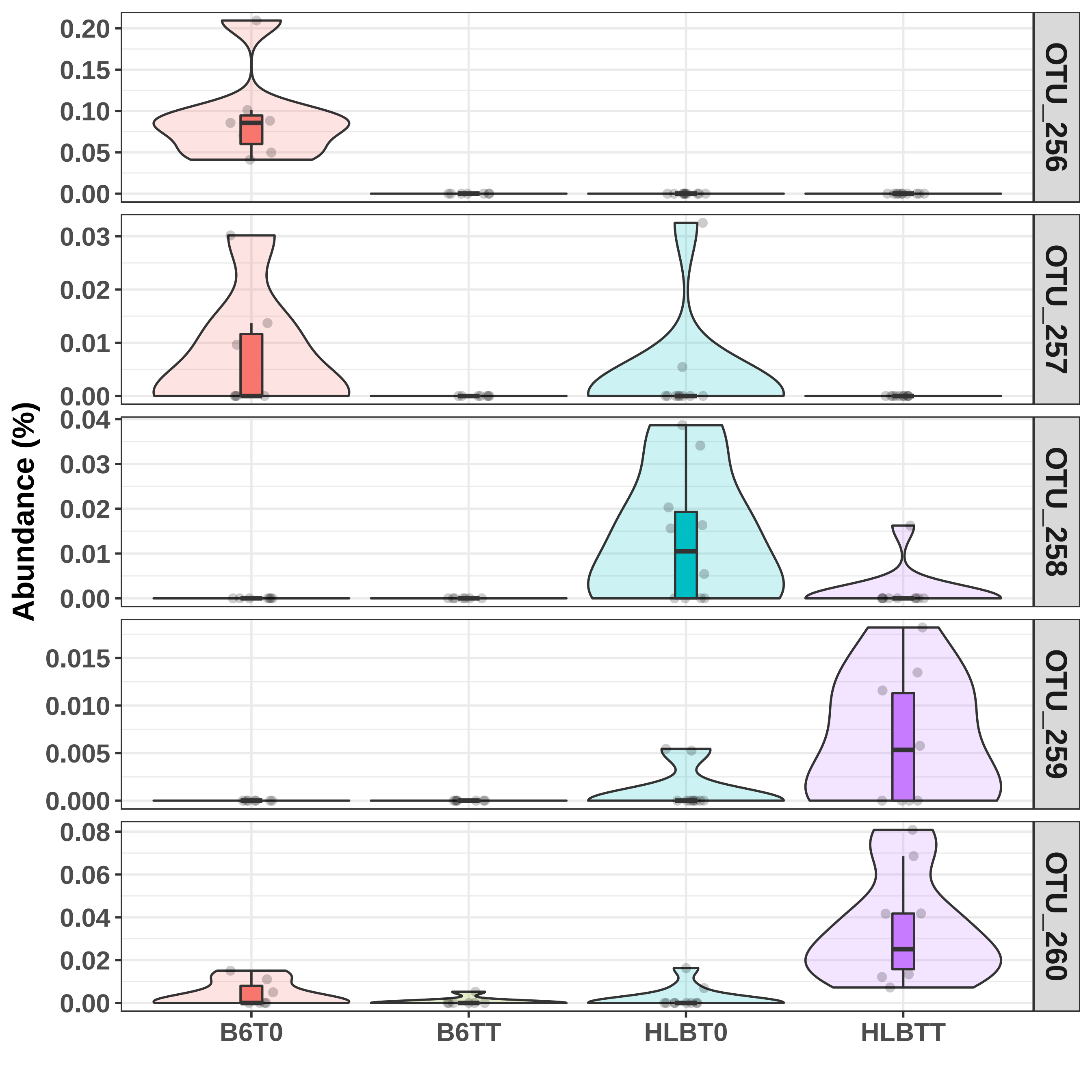

Abundance (%)
OTU_256
OTU_257
OTU_258
OTU_259
OTU_260
0.20
0.15
0.10
0.05
0.00
0.03
0.02
0.01
0.00
0.04
0.03
0.02
0.01
0.00
0.015
0.010
0.005
0.000
0.08
0.06
0.04
0.02
0.00
B6T0
B6TT
HLBT0
HLBTT

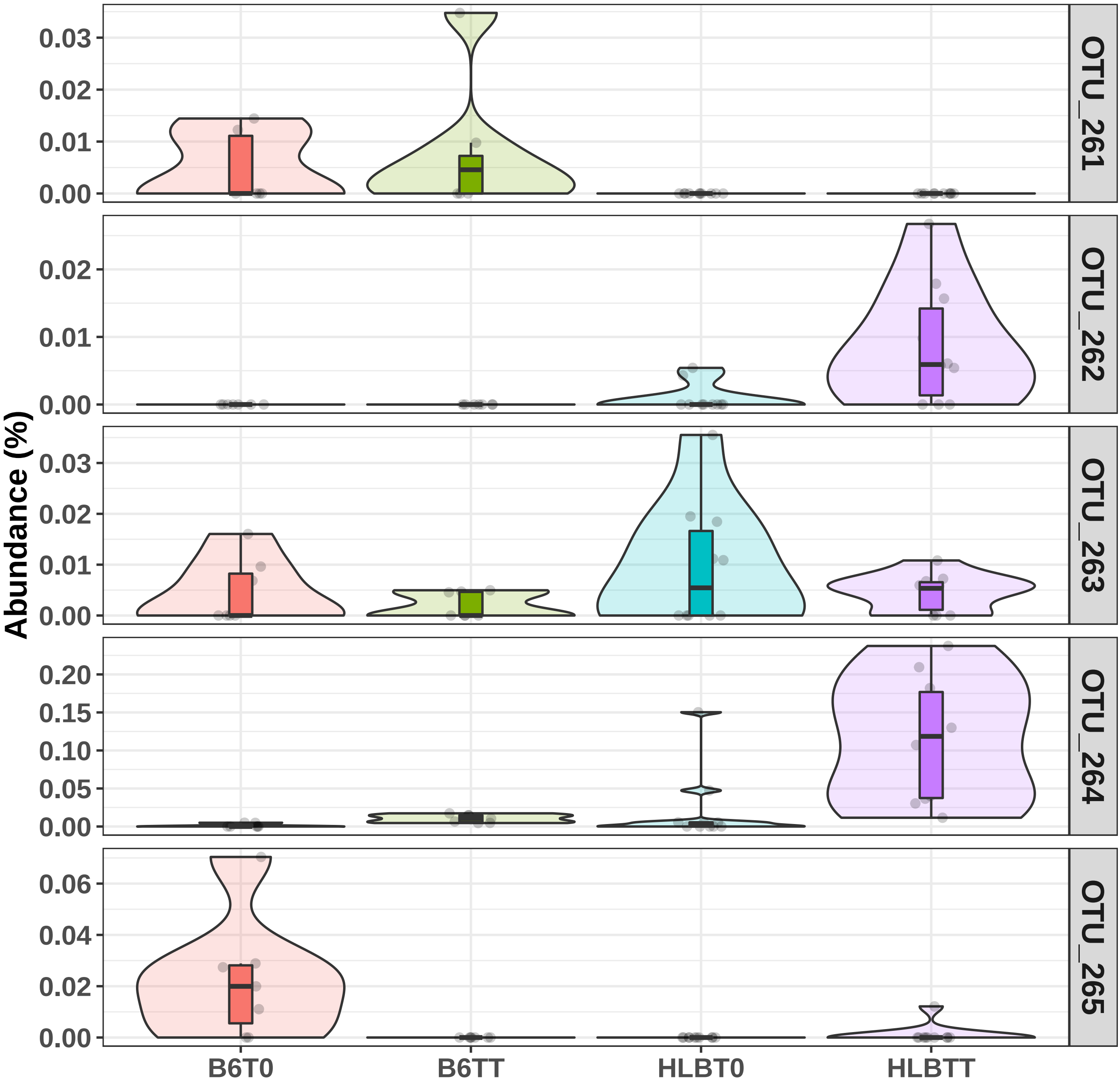
Abundance (%)
OTU_261
OTU_262
OTU_263
OTU_264
OTU_265
0.00
0.01
0.02
0.03
0.05
0.10
0.15
0.20
0.04
0.06
B6T0
B6TT
HLBT0
HLBTT

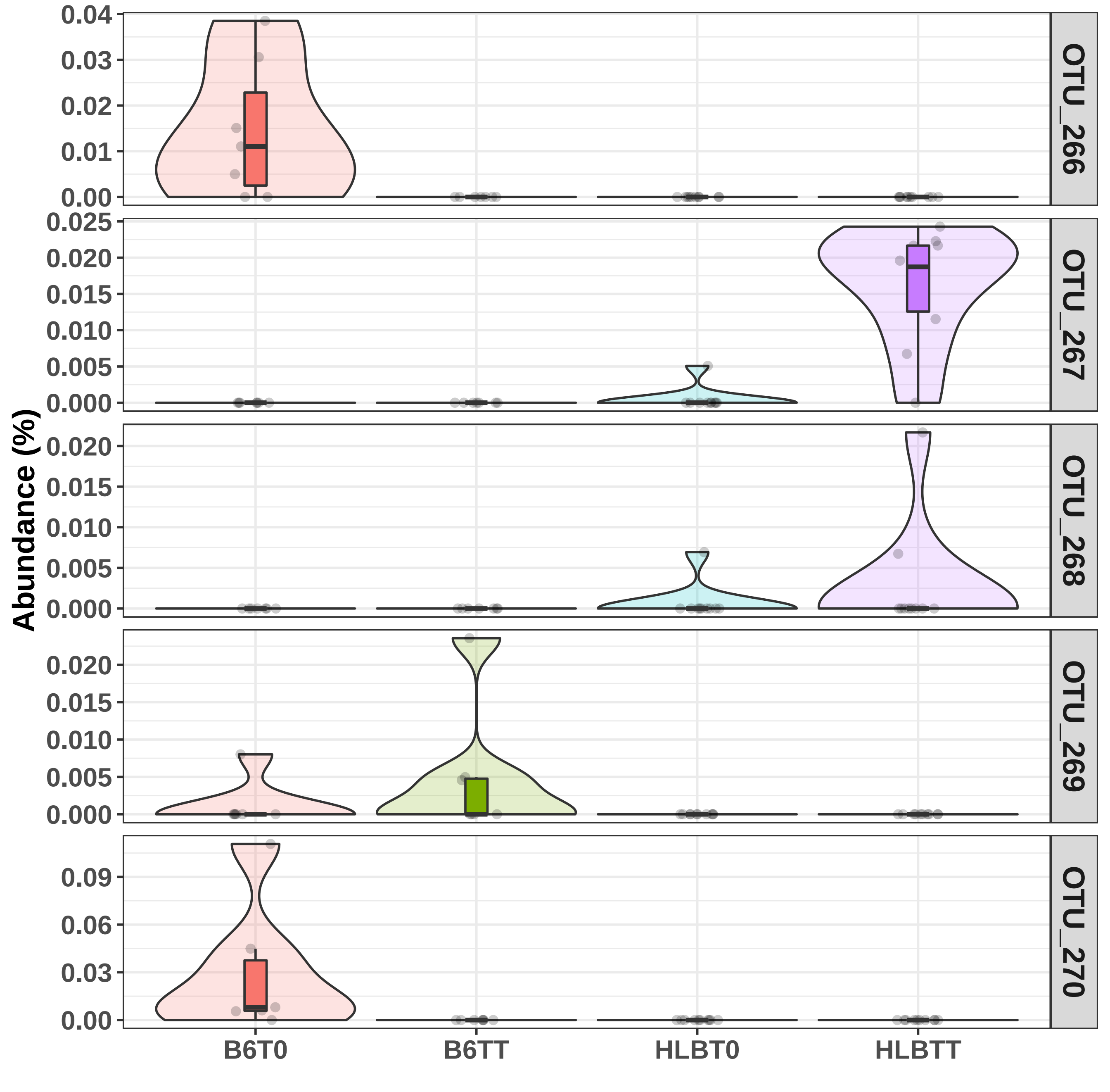
Abundance (%)
OTU_266
OTU_267
OTU_268
OTU_269
OTU_270
B6T0
B6TT
HLBT0
HLBTT
0.00
0.01
0.02
0.03
0.04
0.000
0.005
0.010
0.015
0.020
0.025
0.06
0.09

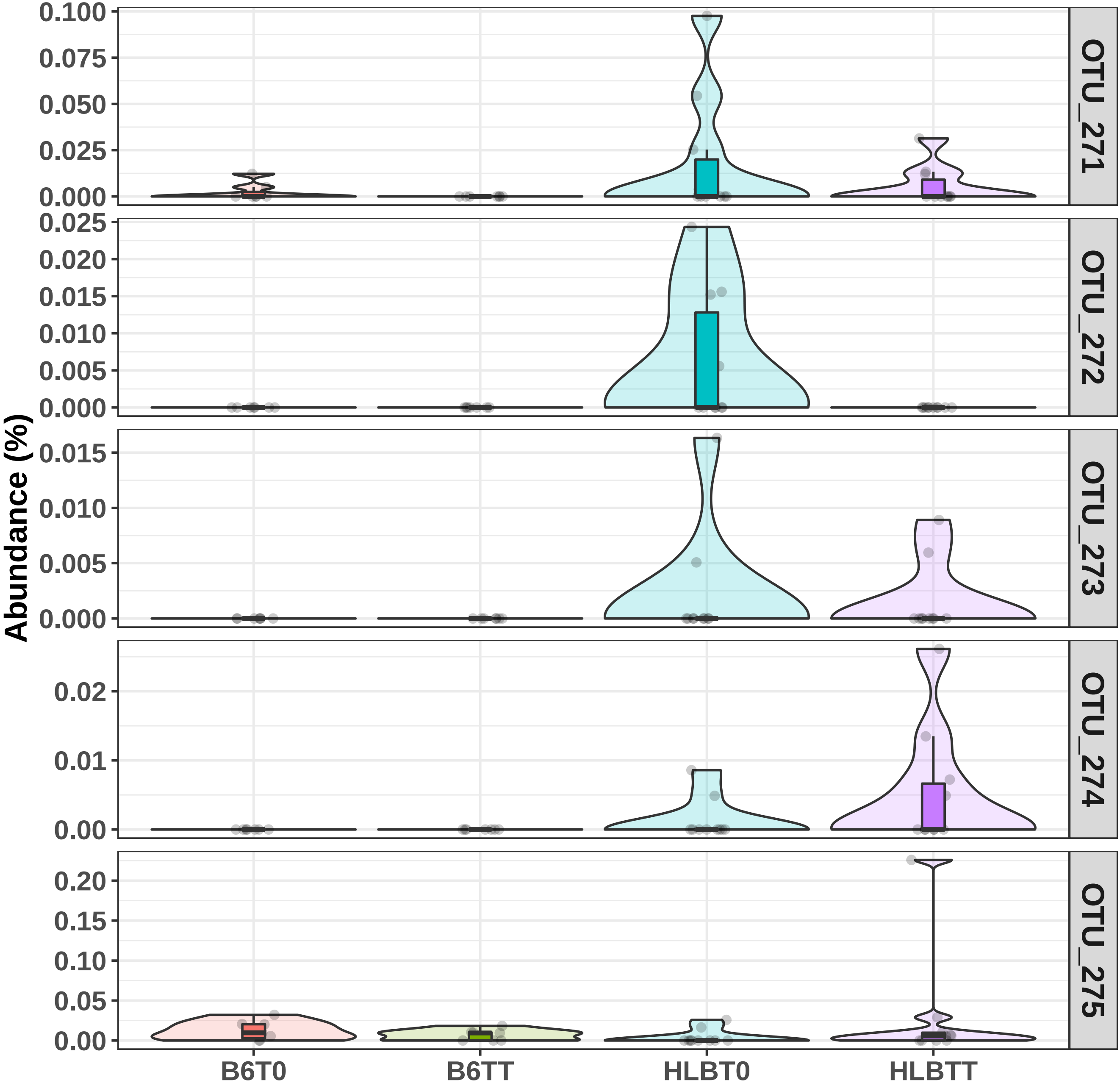

Abundance (%)
OTU_271
OTU_272
OTU_273
OTU_274
OTU_275
0.100
0.075
0.050
0.025
0.000
0.025
0.020
0.015
0.010
0.005
0.000
0.015
0.010
0.005
0.000
0.02
0.01
0.00
0.20
0.15
0.10
0.05
0.00
B6T0
B6TT
HLBT0
HLBTT

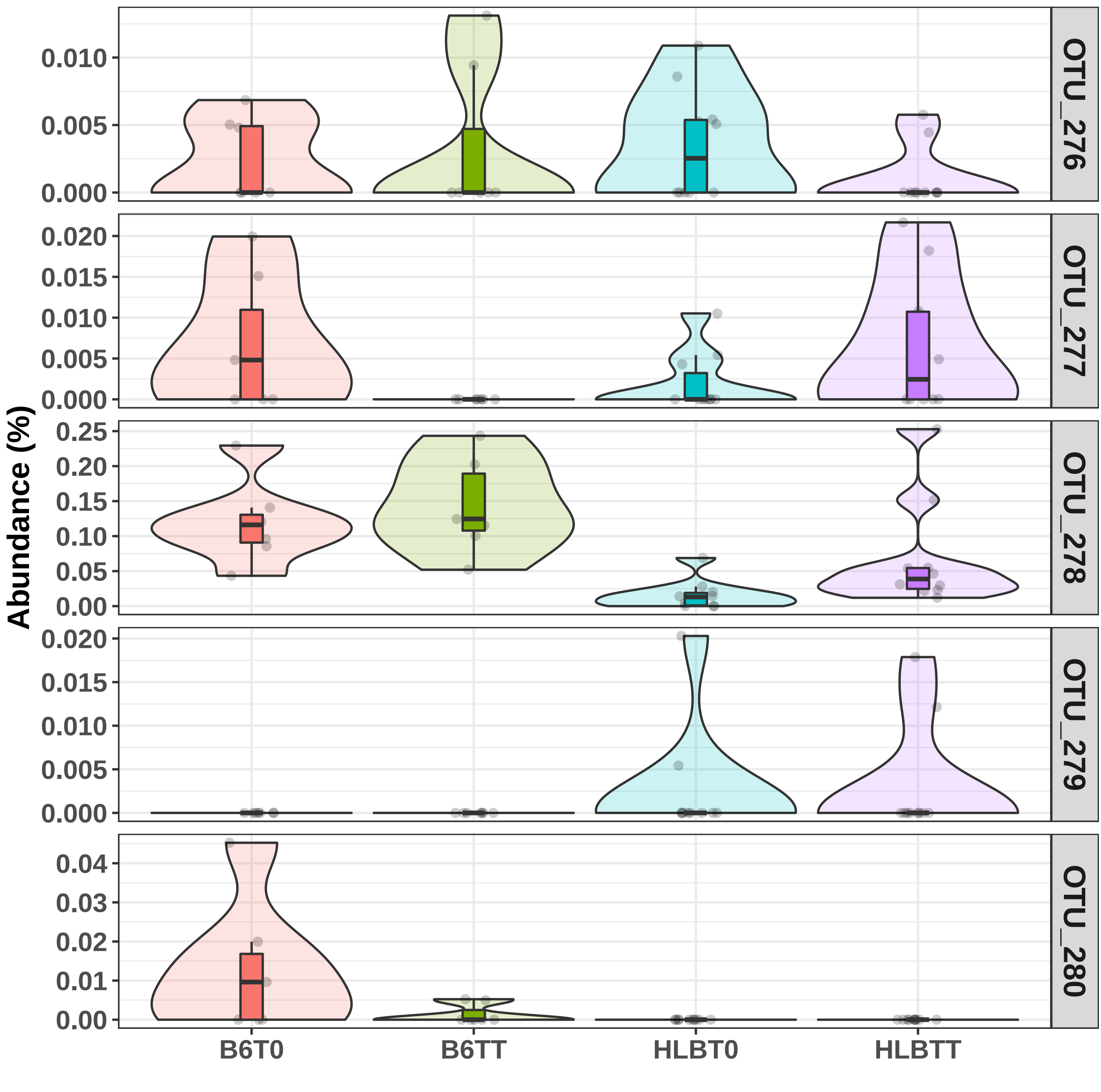

Abundance (%)
0.010
0.005
0.000
OTU_276
0.020
0.015
0.010
0.005
0.000
OTU_277
0.25
0.20
0.15
0.10
0.05
0.00
OTU_278
0.020
0.015
0.010
0.005
0.000
OTU_279
0.04
0.03
0.02
0.01
0.00
OTU_280
B6T0
B6TT
HLBT0
HLBTT

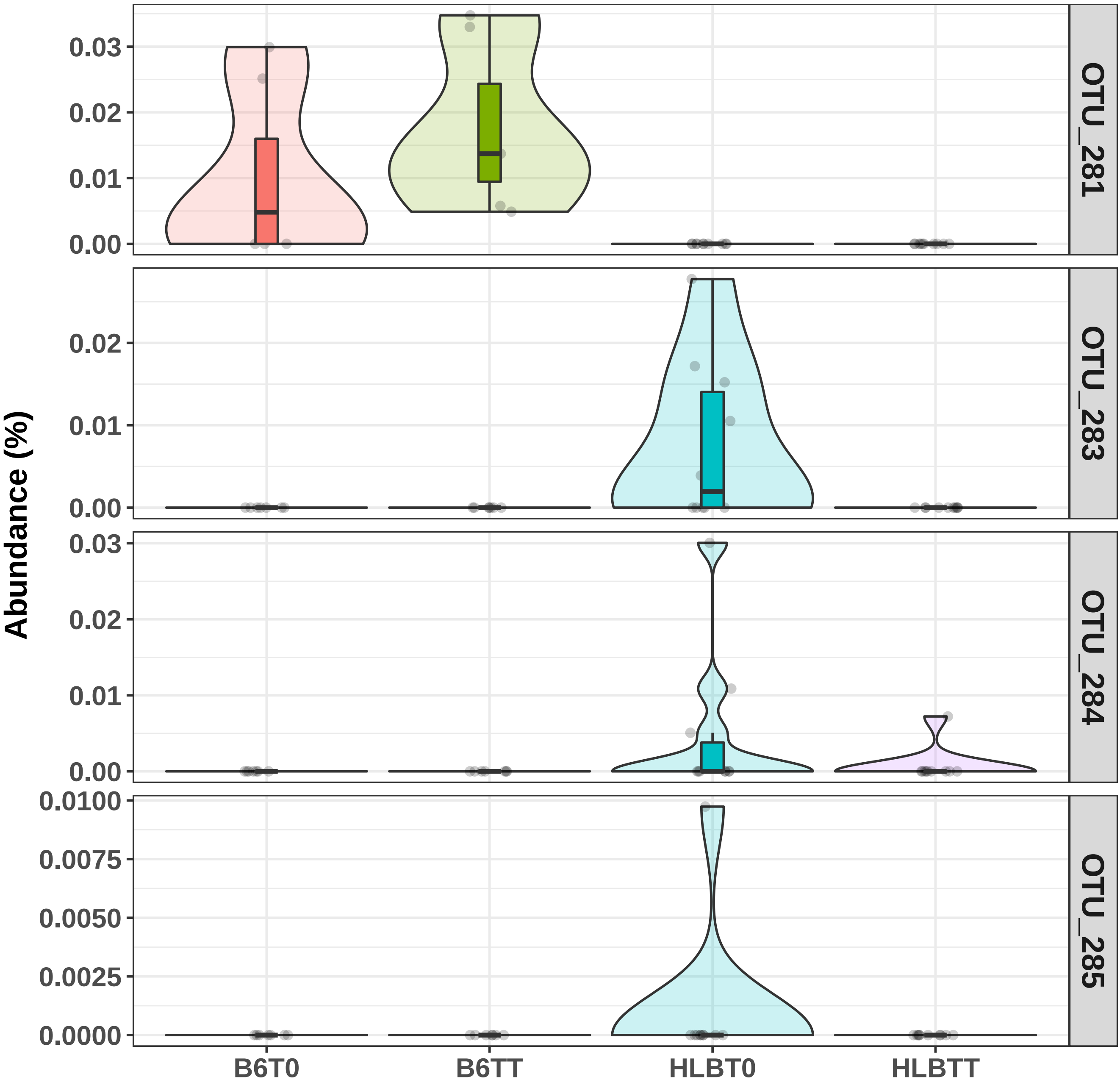

Abundance (%)
OTU_281
OTU_283
OTU_284
OTU_285
0.03
0.02
0.01
0.00
0.0100
0.0075
0.0050
0.0025
0.0000
B6T0
B6TT
HLBT0
HLBTT

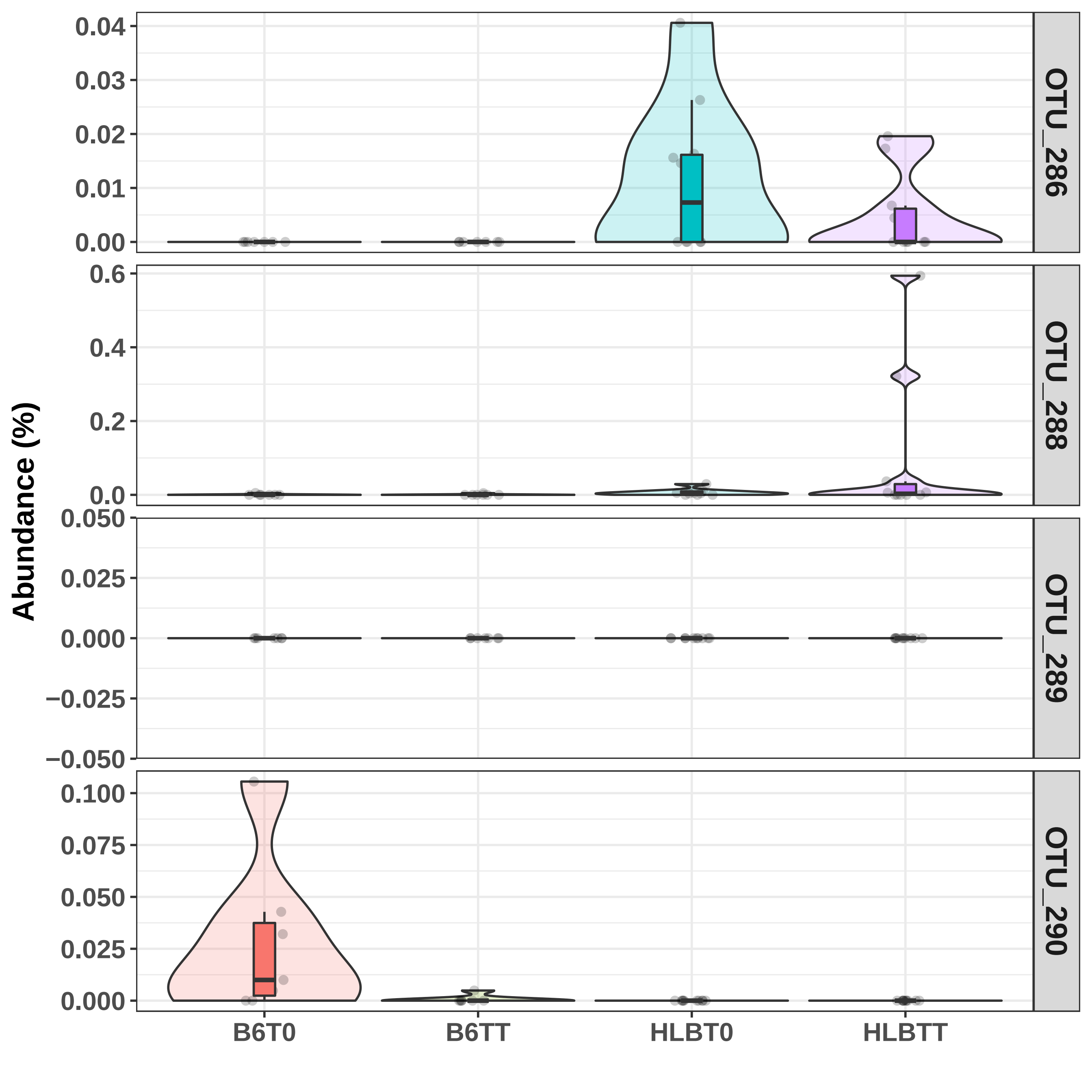
Abundance (%)
OTU_286
OTU_288
OTU_289
OTU_290
0.04
0.03
0.02
0.01
0.00
0.6
0.4
0.2
0.0
0.050
0.025
0.000
−0.025
−0.050
0.100
0.075
0.050
0.025
0.000
B6T0
B6TT
HLBT0
HLBTT

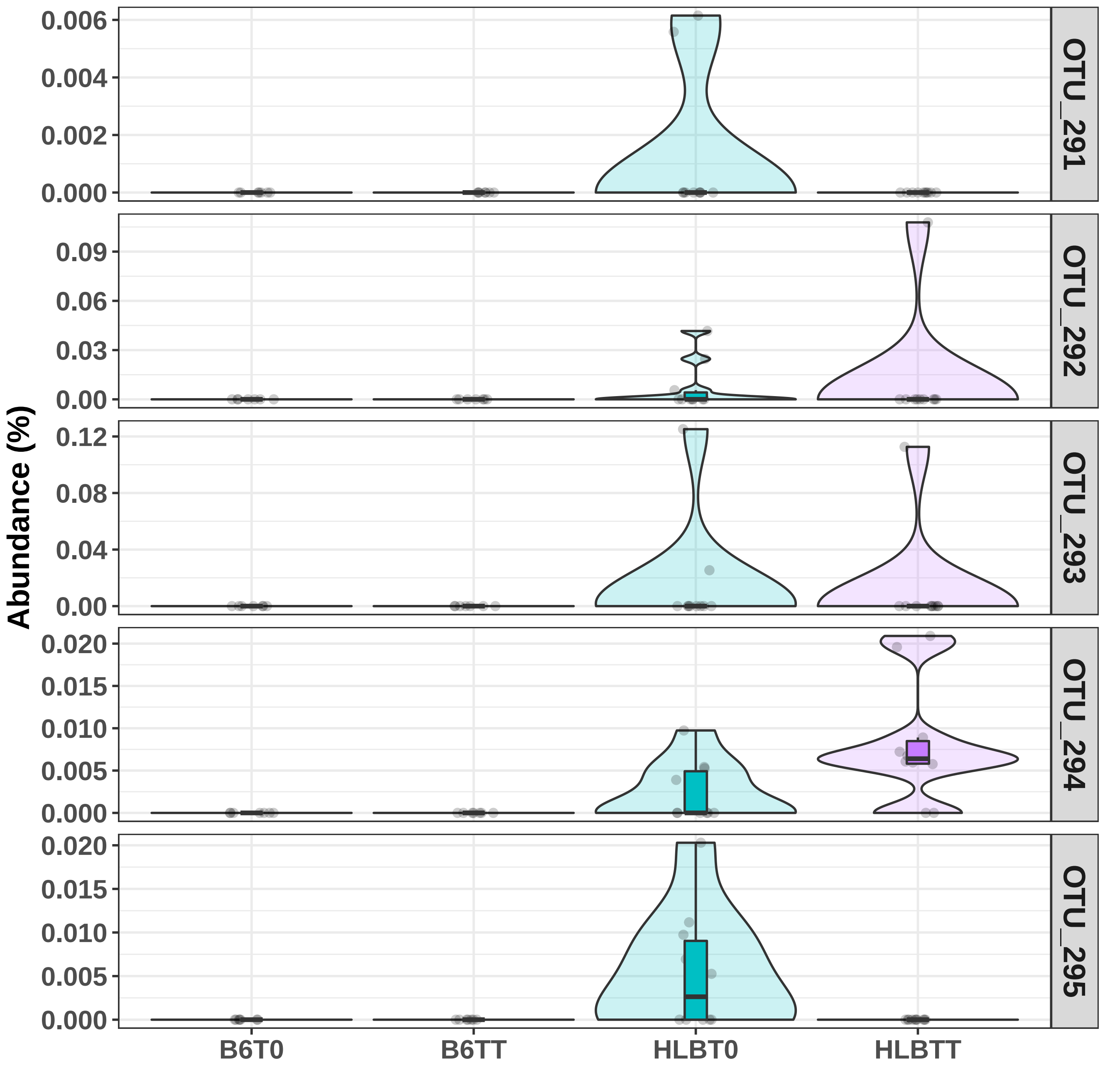
Abundance (%)
OTU_291
OTU_292
OTU_293
OTU_294
OTU_295
0.006
0.004
0.002
0.000
0.09
0.06
0.03
0.00
0.12
0.08
0.04
0.00
0.020
0.015
0.010
0.005
0.000
0.020
0.015
0.010
0.005
0.000
B6T0
B6TT
HLBT0
HLBTT

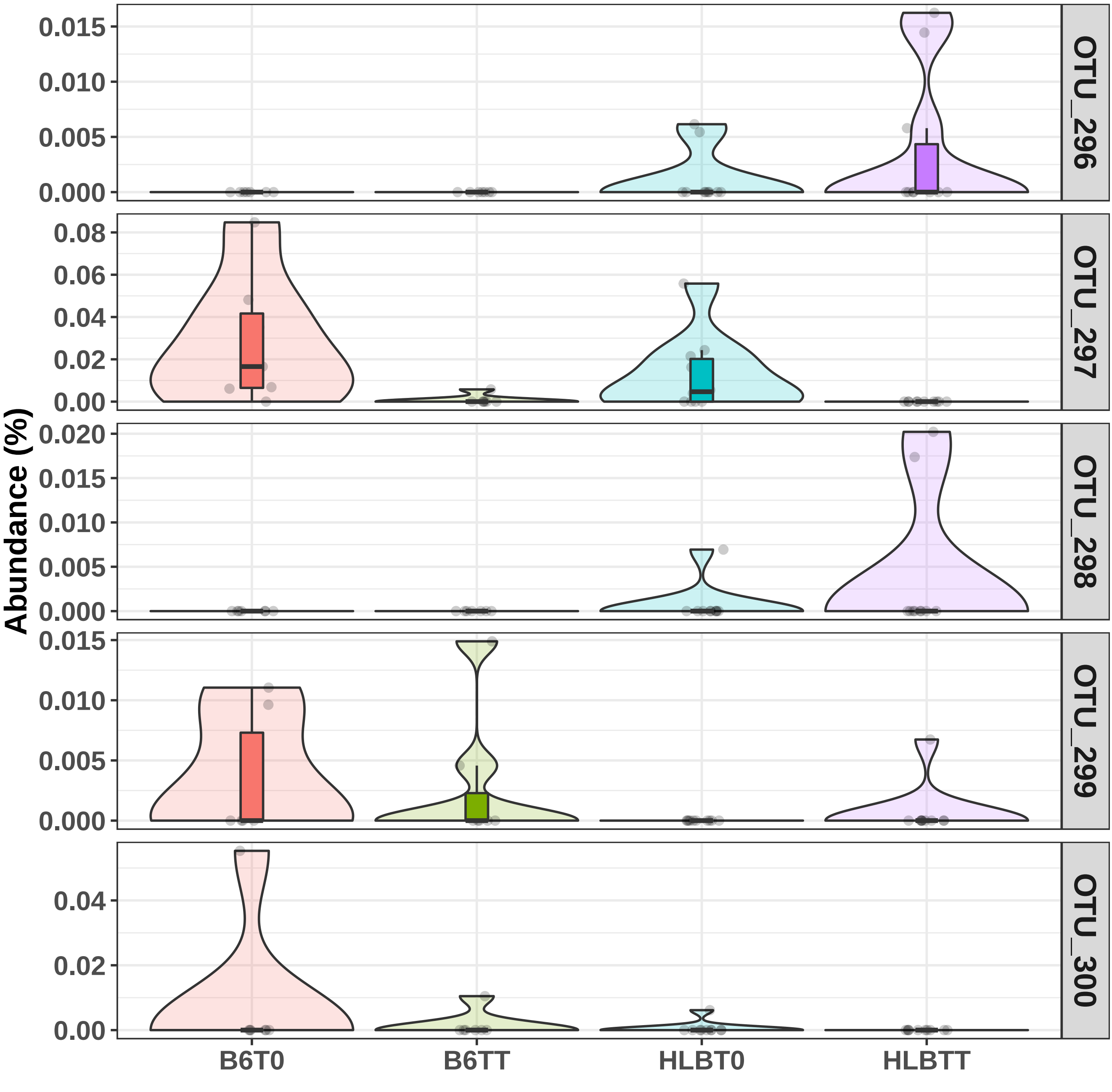

Abundance (%)
OTU_296
OTU_297
OTU_298
OTU_299
OTU_300
0.015
0.010
0.005
0.000
0.08
0.06
0.04
0.02
0.00
0.020
0.015
0.010
0.005
0.000
0.015
0.010
0.005
0.000
0.04
0.02
0.00
B6T0
B6TT
HLBT0
HLBTT

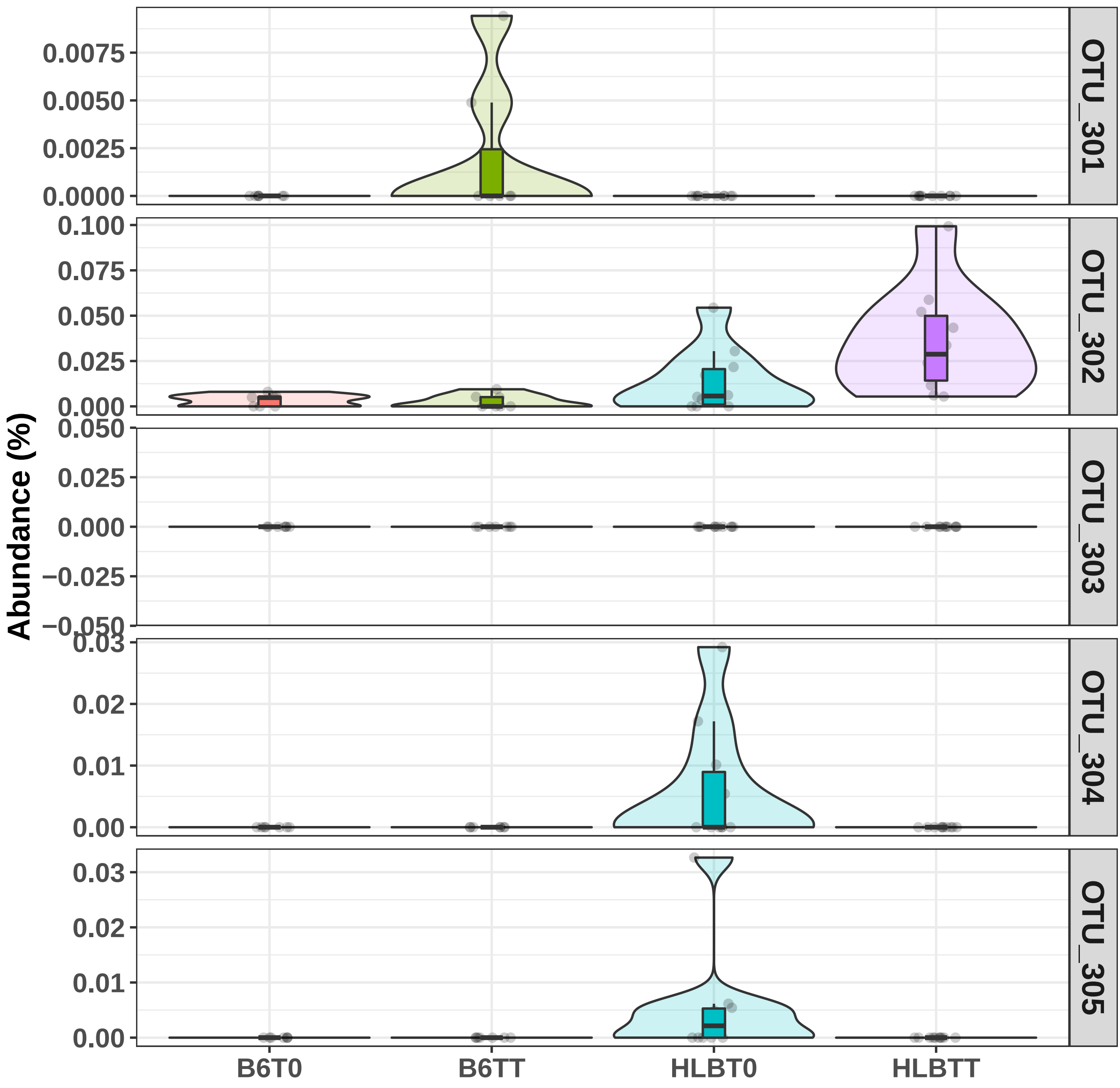

Abundance (%)
0.0075
0.0050
0.0025
0.0000
0.100
0.075
0.050
0.025
0.000
0.050
0.025
0.000
−0.025
−0.050
0.03
0.02
0.01
0.00
0.03
0.02
0.01
0.00
OTU_301
OTU_302
OTU_303
OTU_304
OTU_305
B6T0
B6TT
HLBT0
HLBTT

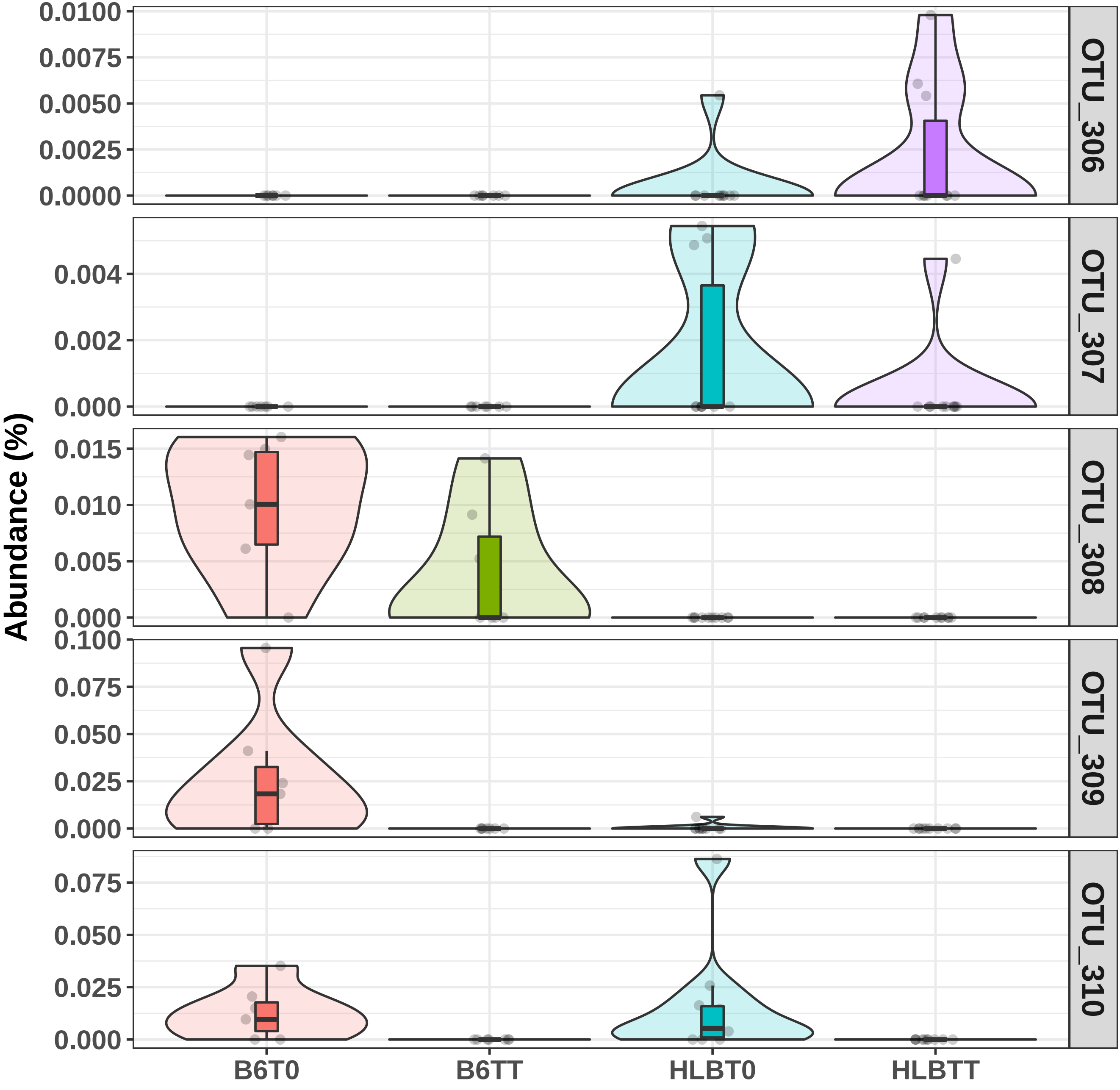

Abundance (%)
OTU_306
OTU_307
OTU_308
OTU_309
OTU_310
0.0100
0.0075
0.0050
0.0025
0.0000
0.004
0.002
0.000
0.015
0.010
0.005
0.000
0.100
0.075
0.050
0.025
0.000
0.075
0.050
0.025
0.000
B6T0
B6TT
HLBT0
HLBTT

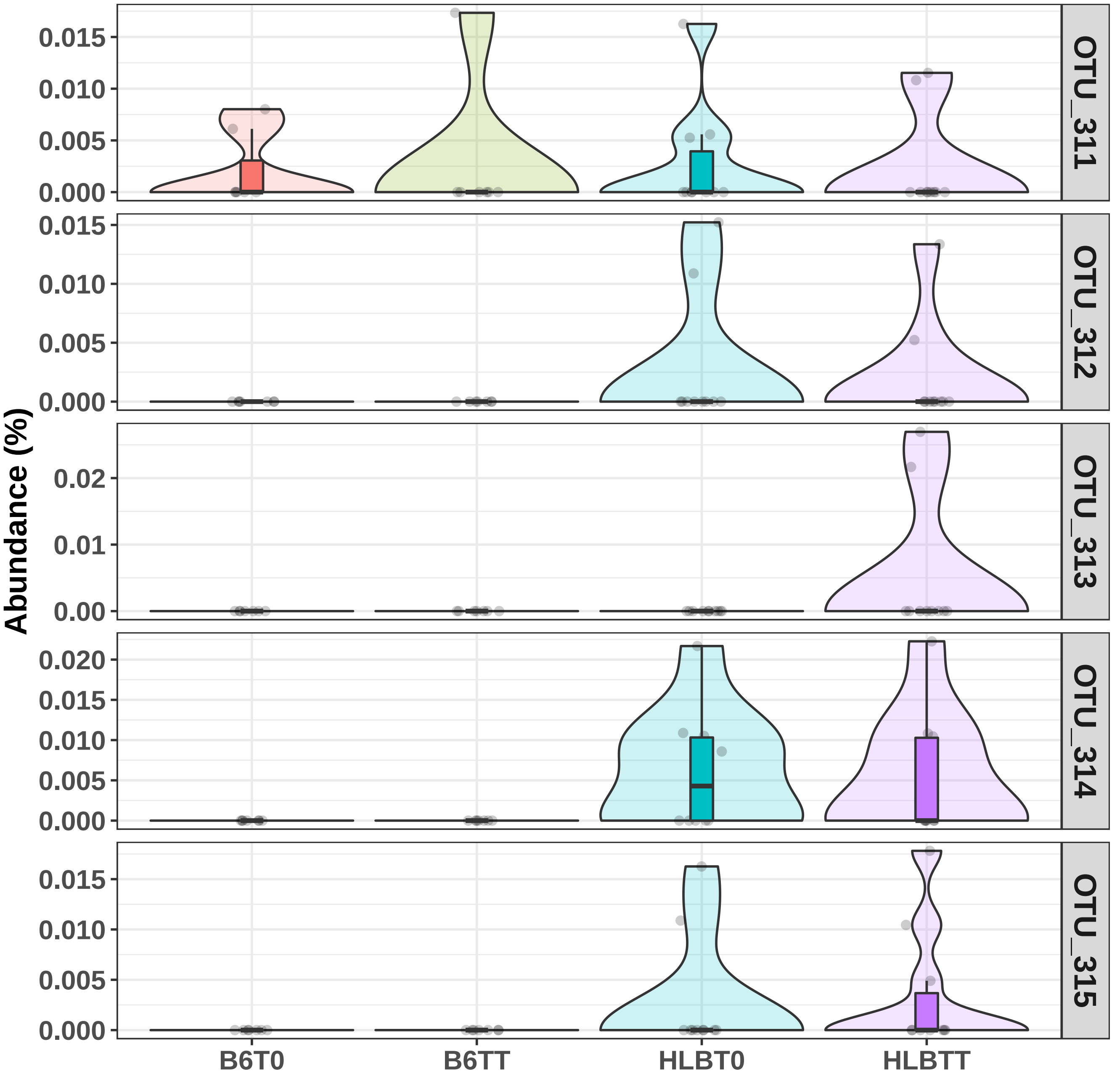

Abundance (%)
OTU_311
OTU_312
OTU_313
OTU_314
OTU_315
0.000
0.005
0.010
0.015
0.00
0.01
0.02
0.020
B6T0
B6TT
HLBT0
HLBTT

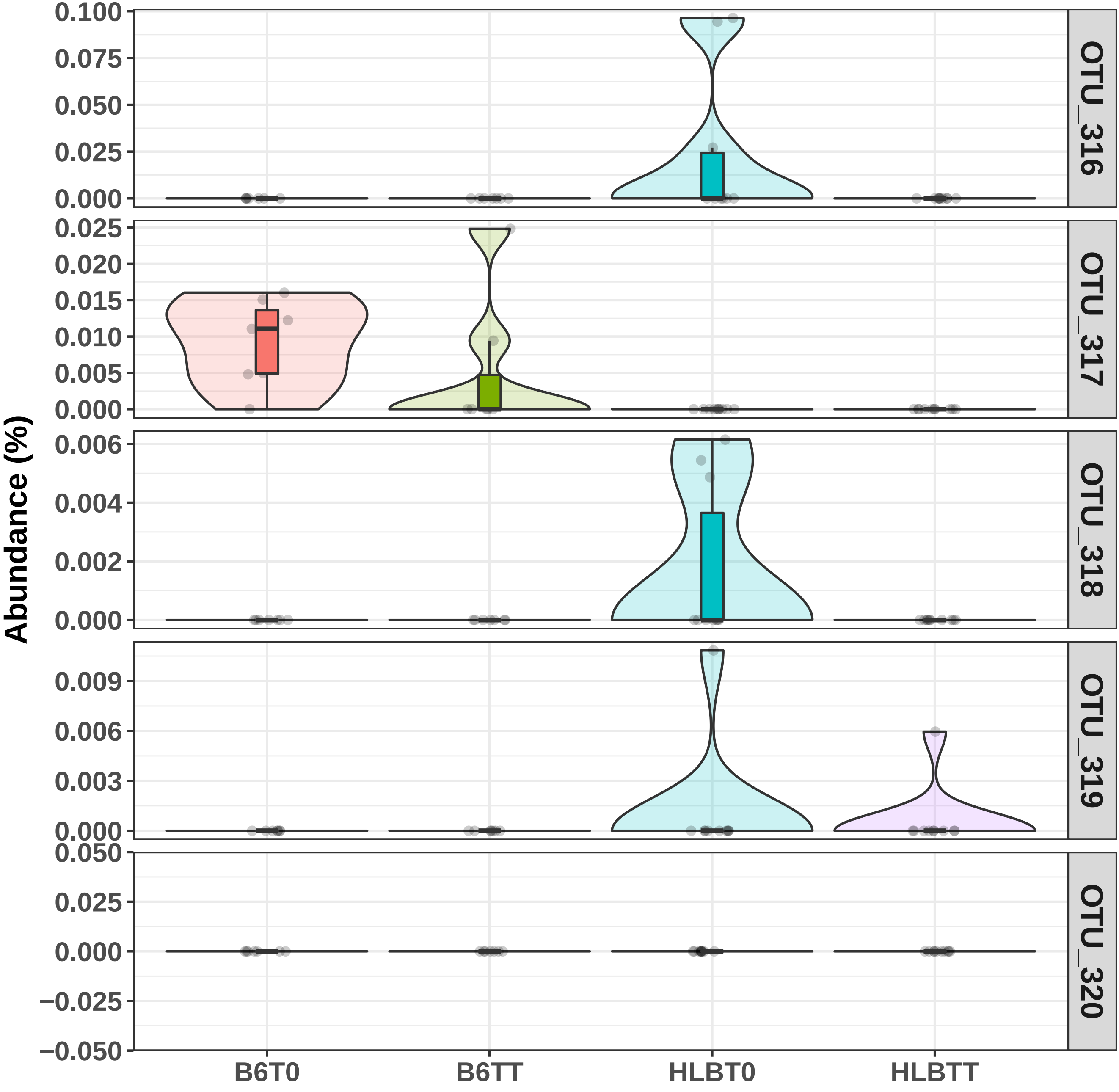

Abundance (%)
OTU_316
OTU_317
OTU_318
OTU_319
OTU_320
0.100
0.075
0.050
0.025
0.000
0.025
0.020
0.015
0.010
0.005
0.000
0.006
0.004
0.002
0.000
0.009
0.006
0.003
0.000
0.050
0.025
0.000
−0.025
−0.050
B6T0
B6TT
HLBT0
HLBTT

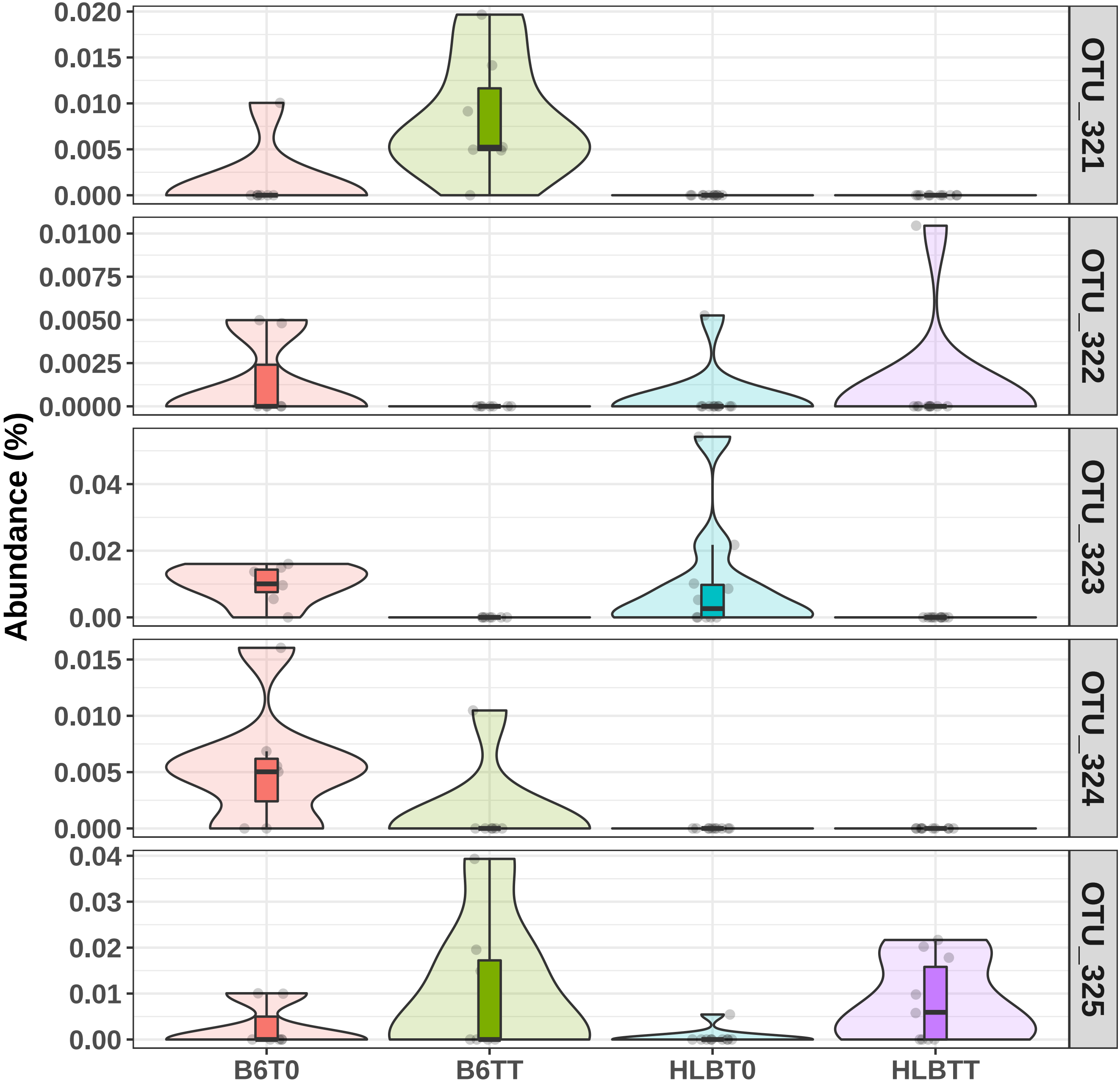

Abundance (%)
OTU_321
OTU_322
OTU_323
OTU_324
OTU_325
0.020
0.015
0.010
0.005
0.000
0.0100
0.0075
0.0050
0.0025
0.0000
0.04
0.02
0.00
0.015
0.010
0.005
0.000
0.04
0.03
0.02
0.01
0.00
B6T0
B6TT
HLBT0
HLBTT

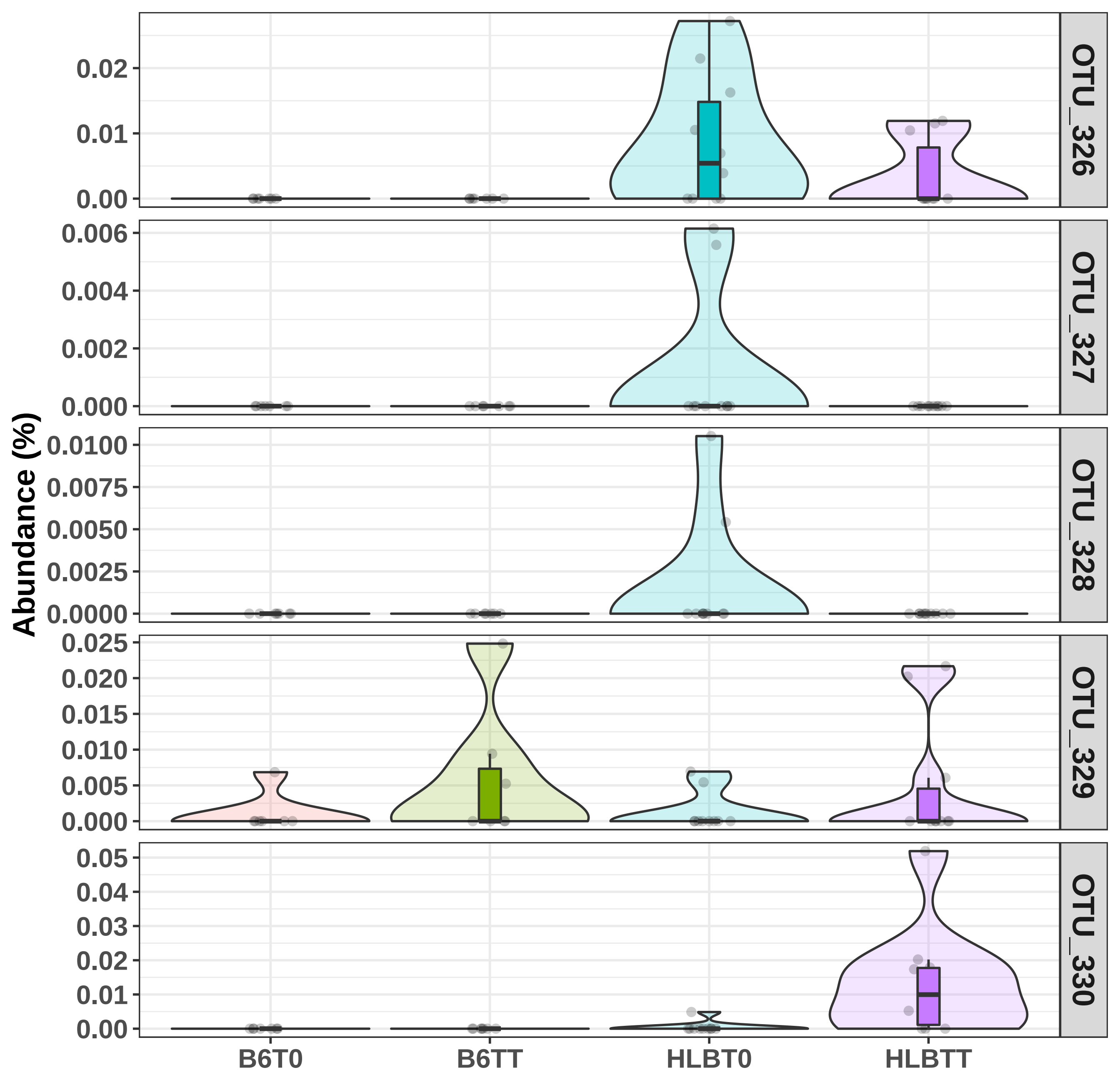

Abundance (%)
OTU_326
OTU_327
OTU_328
OTU_329
OTU_330
0.00
0.01
0.02
0.000
0.002
0.004
0.006
0.0000
0.0025
0.0050
0.0075
0.0100
0.000
0.005
0.010
0.015
0.020
0.025
0.00
0.01
0.02
0.03
0.04
0.05
B6T0
B6TT
HLBT0
HLBTT

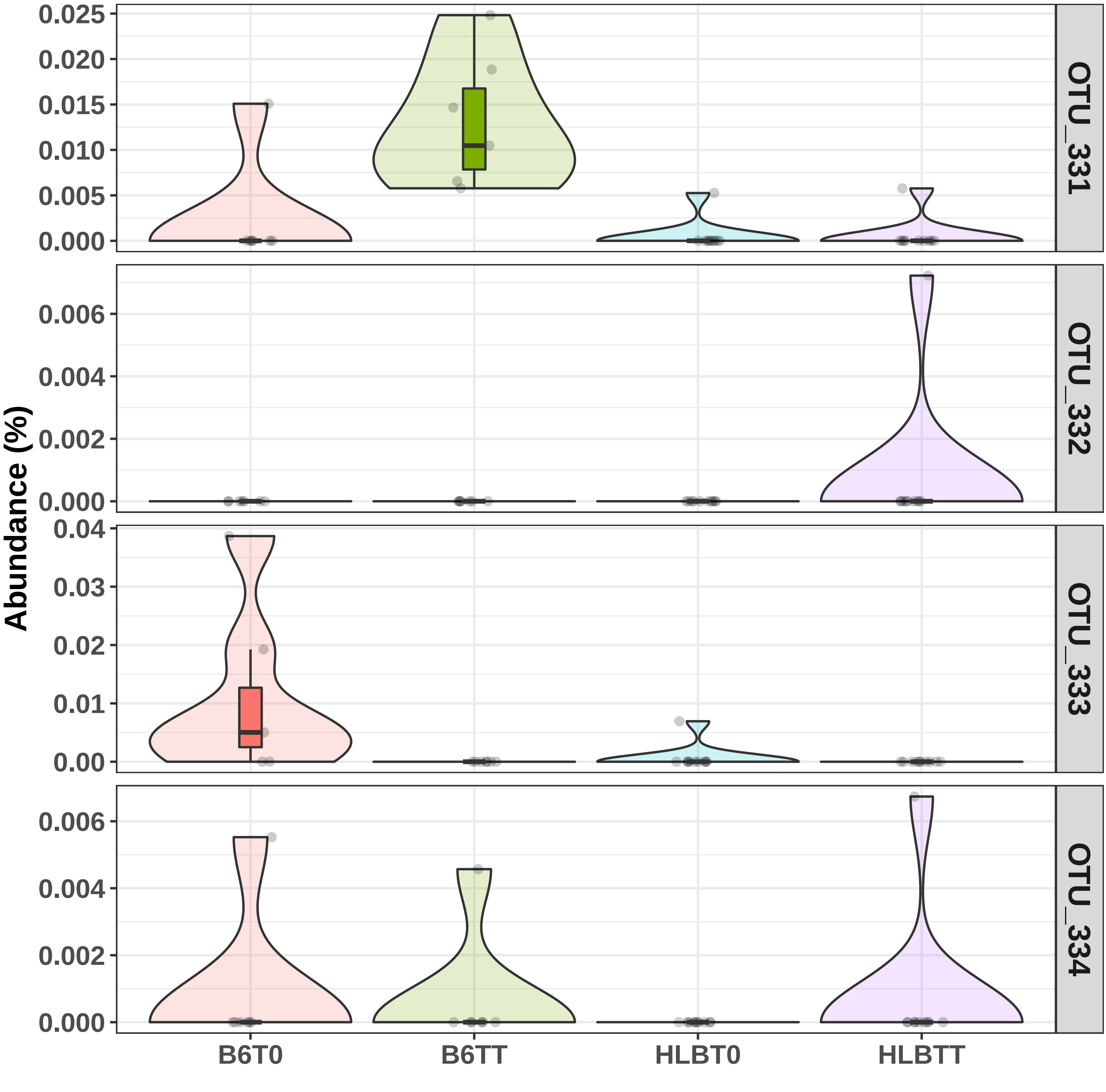

Abundance (%)
OTU_331
OTU_332
OTU_333
OTU_334
0.025
0.020
0.015
0.010
0.005
0.000
0.006
0.004
0.002
0.04
0.03
0.02
0.01
0.00
B6T0
B6TT
HLBT0
HLBTT

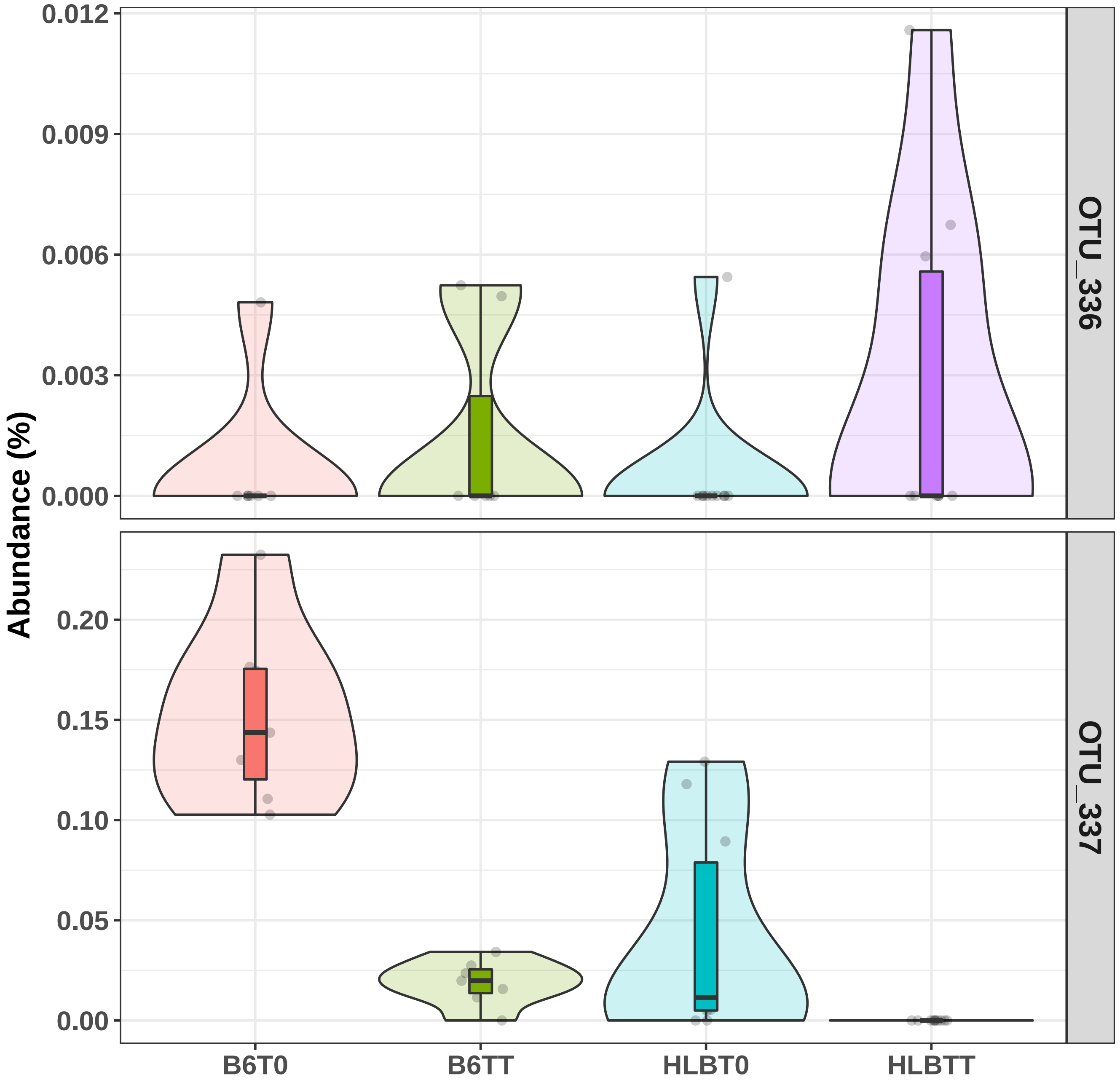

Abundance (%)
0.012
0.009
0.006
0.003
0.000
OTU_336
0.20
0.15
0.10
0.05
0.00
OTU_337
B6T0
B6TT
HLBT0
HLBTT
