# Supplementary material for: A mutation in mouse Krüppel-like factor 15 alters the gut microbiome and response to obesogenic diet
Source: PLoS One. 2019 Sep 25;14(9):e0222536. doi: 10.1371/journal.pone.0222536 (PMC6760833; doi:10.1371/journal.pone.0222536)
Supplement: S1 Table — (PDF) [file pone.0222536.s005.pdf]

Table S1. Liver RNAseq GSEA

	Size	ES	NES	Nom. p-val	FDR q-val.	FWER p-val.	Rank at Max	Leading Edge
**S1.1**	**Hallmark gene sets; Increased in HLB444**							
**hallmark unfolded protein response**	105	0.52	2.13	0	0	0	2413	tags=47%, list=23%, signal=60%
**S1.2**	**Hallmark gene sets; Decreased in HLB444**							
**hallmark fatty acid metabolism**	140	-0.49	-1.99	0	0.001	0.001	2298	tags=48%, list=22%, signal=60%
**hallmark adipogenesis**	180	-0.47	-1.97	0	0.001	0.001	2078	tags=38%, list=20%, signal=46%
**hallmark interferon gamma response**	168	-0.47	-1.95	0	0	0.001	1785	tags=37%, list=17%, signal=44%
**hallmark interferon alpha response**	85	-0.51	-1.93	0	0.001	0.002	2640	tags=53%, list=25%, signal=70%
**hallmark oxidative phosphorylation**	180	-0.45	-1.87	0	0.001	0.003	3190	tags=46%, list=30%, signal=65%
**hallmark xenobiotic metabolism**	180	-0.44	-1.84	0	0.001	0.004	1587	tags=35%, list=15%, signal=40%
**hallmark bile acid metabolism**	104	-0.47	-1.8	0	0.001	0.006	1610	tags=37%, list=15%, signal=43%
**hallmark peroxisome**	90	-0.45	-1.72	0	0.004	0.026	2716	tags=44%, list=25%, signal=59%
**hallmark allograft rejection**	116	-0.38	-1.48	0.013	0.059	0.399	2205	tags=33%, list=21%, signal=41%

ES = Enrichment Score; NES = Normalized Enrichment Score; FWER = Family-Wise Error Rate

Table S1 cont. Liver RNAseq GSEA

S1.3	GO Terms; Increased in HLB444 relative to Control							
	Size	*ES*	*NES*	*Nom. p-val*	*FDR q-val.*	*FWER p-val.*	*Rank at Max*	*Leading Edge*
**GO response to topologically incorrect protein**	137	0.54	2.31	0	0.002	0.002	2387	tags=42%, list=20%, signal=52%
**GO ER nucleus signaling pathway**	31	0.67	2.17	0	0.01	0.017	923	tags=35%, list=8%, signal=38%
**GO cellular response to topologically incorrect protein**	103	0.51	2.12	0	0.016	0.042	2714	tags=45%, list=23%, signal=57%
**GO unfolded protein binding**	76	0.53	2.08	0	0.022	0.075	1216	tags=29%, list=10%, signal=32%
**GO protein refolding**	15	0.75	2.03	0	0.036	0.147	430	tags=40%, list=4%, signal=41%
**GO regulation of cellular response to heat**	66	0.53	2.02	0	0.033	0.164	2034	tags=36%, list=17%, signal=44%
**GO peptidyl asparagine modification**	34	0.6	2	0	0.042	0.229	2433	tags=50%, list=20%, signal=63%
**GO response to endoplasmic reticulum stress**	196	0.44	1.98	0	0.046	0.28	2714	tags=40%, list=23%, signal=51%
**GO protein N linked glycosylation**	63	0.53	1.98	0	0.041	0.282	2802	tags=51%, list=23%, signal=66%
**GO IRE1 mediated unfolded protein response**	51	0.54	1.95	0	0.054	0.385	2697	tags=53%, list=22%, signal=68%
**GO ER associated ubiquitin dependent protein catabolic process**	57	0.53	1.94	0	0.056	0.427	1220	tags=28%, list=10%, signal=31%
**GO ERAD pathway**	67	0.5	1.91	0	0.077	0.56	2208	tags=31%, list=18%, signal=38%

ES = Enrichment Score; NES = Normalized Enrichment Score; FWER = Family-Wise Error Rate

Table S1 cont. Liver RNAseq GSEA

S1.4	GO Terms; Decreased in HLB444 relative to Control							
	Size	*ES*	*NES*	*Nom. p-val*	*FDR q-val.*	*FWER p-val.*	*Rank at Max*	*Leading Edge*
**GO cellular amino acid catabolic process**	93	-0.65	-2.45	0	0	0	771	tags=35%, list=6%, signal=38%
**GO carboxylic acid catabolic process**	173	-0.57	-2.38	0	0	0	2121	tags=45%, list=18%, signal=53%
**GO small molecule catabolic process**	260	-0.54	-2.35	0	0	0	2121	tags=40%, list=18%, signal=48%
**GO alpha amino acid catabolic process**	79	-0.63	-2.35	0	0	0	734	tags=32%, list=6%, signal=33%
**GO organic acid catabolic process**	173	-0.57	-2.34	0	0	0	2121	tags=45%, list=18%, signal=53%
**GO ADP metabolic process**	32	-0.72	-2.23	0	0	0.002	1132	tags=41%, list=9%, signal=45%
**GO ribonucleoside diphosphate metabolic process**	44	-0.67	-2.22	0	0	0.003	1132	tags=34%, list=9%, signal=37%
**GO pyruvate metabolic process**	45	-0.65	-2.15	0	0.001	0.008	1427	tags=40%, list=12%, signal=45%
**GO branched chain amino acid metabolic process**	22	-0.75	-2.15	0	0.001	0.008	1033	tags=55%, list=9%, signal=60%
**GO monosaccharide catabolic process**	40	-0.66	-2.12	0	0.002	0.02	1132	tags=38%, list=9%, signal=41%
**GO ATP generation from ADP**	26	-0.71	-2.11	0	0.002	0.02	1132	tags=42%, list=9%, signal=47%
**GO monocarboxylic acid catabolic process**	83	-0.56	-2.09	0	0.002	0.027	2466	tags=52%, list=21%, signal=65%
**GO nucleoside diphosphate metabolic process**	56	-0.6	-2.07	0	0.003	0.036	1287	tags=30%, list=11%, signal=34%
**GO positive regulation of anion transport**	30	-0.68	-2.06	0	0.003	0.048	2421	tags=53%, list=20%, signal=67%
**GO nucleotide phosphorylation**	39	-0.64	-2.06	0	0.003	0.05	1287	tags=36%, list=11%, signal=40%
**GO coenzyme binding**	149	-0.51	-2.05	0	0.003	0.057	2496	tags=38%, list=21%, signal=48%
**GO hexose catabolic process**	32	-0.65	-2.05	0	0.003	0.061	1132	tags=38%, list=9%, signal=41%

ES = Enrichment Score; NES = Normalized Enrichment Score; FWER = Family-Wise Error Rate

Table S1 cont. Liver RNAseq GSEA

S1.4	GO Terms; Decreased in HLB444 relative to Control							
	Size	*ES*	*NES*	*Nom. p-val*	*FDR q-val.*	*FWER p-val.*	*Rank at Max*	*Leading Edge*
**GO carbohydrate catabolic process**	75	-0.55	-2.04	0	0.004	0.077	1162	tags=29%, list=10%, signal=32%
**GO glutathione derivative biosynthetic process**	15	-0.78	-2.02	0	0.005	0.106	1564	tags=60%, list=13%, signal=69%
**GO glutathione derivative metabolic process**	15	-0.78	-2.02	0	0.005	0.107	1564	tags=60%, list=13%, signal=69%
**GO cellular modified amino acid metabolic process**	145	-0.49	-2.01	0	0.005	0.115	1413	tags=30%, list=12%, signal=33%
**GO NADH metabolic process**	28	-0.65	-2	0	0.008	0.165	1132	tags=32%, list=9%, signal=35%
**GO oxidoreduction coenzyme metabolic process**	82	-0.54	-2	0	0.008	0.168	1427	tags=27%, list=12%, signal=30%
**GO coenzyme metabolic process**	215	-0.47	-1.99	0	0.008	0.185	1442	tags=26%, list=12%, signal=29%
**GO fatty acyl coA binding**	28	-0.65	-1.99	0	0.008	0.193	2355	tags=57%, list=20%, signal=71%
**GO monocarboxylic acid metabolic process**	346	-0.44	-1.96	0	0.013	0.306	1919	tags=34%, list=16%, signal=40%
**GO oxidoreductase activity acting on the CH CH group of donors**	50	-0.58	-1.96	0	0.013	0.306	2355	tags=40%, list=20%, signal=50%
**GO NAD binding**	48	-0.58	-1.95	0	0.013	0.327	2496	tags=42%, list=21%, signal=52%
**GO aromatic amino acid family metabolic process**	20	-0.71	-1.95	0	0.013	0.327	394	tags=45%, list=3%, signal=46%
**GO carbohydrate kinase activity**	17	-0.74	-1.95	0.002	0.012	0.33	1066	tags=53%, list=9%, signal=58%
**GO response to xenobiotic stimulus**	62	-0.56	-1.95	0	0.012	0.331	1803	tags=42%, list=15%, signal=49%
**GO aromatic amino acid family catabolic process**	17	-0.72	-1.95	0	0.012	0.337	394	tags=47%, list=3%, signal=49%
**GO regulation of organic acid transport**	30	-0.64	-1.95	0	0.012	0.34	1293	tags=33%, list=11%, signal=37%
**GO positive regulation of organic acid transport**	17	-0.74	-1.95	0	0.011	0.343	2099	tags=59%, list=17%, signal=71%

ES = Enrichment Score; NES = Normalized Enrichment Score; FWER = Family-Wise Error Rate

Table S1 cont. Liver RNAseq GSEA

S1.4	Size	ES	NES	Nom. p-val	FDR q-val.	FWER p-val.	Rank at Max	Leading Edge
	GO Terms; Decreased in HLB444 relative to Control							
**GO cofactor metabolic process**	276	-0.45	-1.94	0	0.014	0.406	1442	tags=25%, list=12%, signal=27%
**GO oxidoreductase activity acting on CH OH group of donors**	93	-0.51	-1.93	0	0.014	0.424	3220	tags=54%, list=27%, signal=73%
**GO alditol phosphate metabolic process**	21	-0.69	-1.92	0	0.018	0.509	808	tags=33%, list=7%, signal=36%
**GO fatty acid catabolic process**	64	-0.55	-1.91	0	0.018	0.528	2466	tags=56%, list=21%, signal=70%
**GO fatty acid beta oxidation using acyl coA dehydrogenase**	17	-0.71	-1.91	0	0.02	0.571	2355	tags=65%, list=20%, signal=80%
**GO carbohydrate phosphorylation**	18	-0.7	-1.9	0	0.021	0.61	1066	tags=50%, list=9%, signal=55%
**GO organic acid transmembrane transporter activity**	81	-0.51	-1.9	0	0.021	0.614	1487	tags=36%, list=12%, signal=41%
**GO lipid catabolic process**	168	-0.46	-1.9	0	0.021	0.626	2526	tags=46%, list=21%, signal=57%
**GO acetyl coA metabolic process**	22	-0.66	-1.89	0	0.022	0.644	1398	tags=50%, list=12%, signal=56%
**GO G protein beta gamma subunit complex binding**	15	-0.73	-1.89	0.002	0.023	0.657	207	tags=20%, list=2%, signal=20%
**GO aspartate family amino acid catabolic process**	15	-0.72	-1.88	0.002	0.023	0.675	734	tags=33%, list=6%, signal=35%
**GO cellular lipid catabolic process**	119	-0.47	-1.87	0	0.03	0.765	2526	tags=49%, list=21%, signal=61%
**GO vasodilation**	18	-0.68	-1.86	0.002	0.03	0.783	2197	tags=50%, list=18%, signal=61%
**GO neutral amino acid transmembrane transporter activity**	17	-0.7	-1.86	0	0.03	0.789	669	tags=29%, list=6%, signal=31%
**GO cellular aldehyde metabolic process**	67	-0.51	-1.86	0	0.03	0.792	1408	tags=28%, list=12%, signal=32%
**GO organonitrogen compound catabolic process**	256	-0.43	-1.86	0	0.03	0.802	1767	tags=26%, list=15%, signal=30%
**GO proteasome core complex**	18	-0.69	-1.86	0.002	0.03	0.807	3335	tags=83%, list=28%, signal=115%

ES = Enrichment Score; NES = Normalized Enrichment Score; FWER = Family-Wise Error Rate

Table S1 cont. Liver RNAseq GSEA

S1.4	GO Terms; Decreased in HLB444 relative to Control							
	Size	*ES*	*NES*	*Nom. p-val*	*FDR q-val.*	*FWER p-val.*	*Rank at Max*	*Leading Edge*
**GO electron carrier activity**	93	-0.48	-1.85	0.002	0.031	0.829	3029	tags=46%, list=25%, signal=61%
**GO organic acid transport**	156	-0.46	-1.85	0	0.031	0.832	1592	tags=32%, list=13%, signal=36%
**GO vitamin metabolic process**	80	-0.5	-1.84	0	0.035	0.877	1567	tags=38%, list=13%, signal=43%
**GO acyl coA dehydrogenase activity**	16	-0.69	-1.84	0	0.036	0.896	2355	tags=63%, list=20%, signal=78%
**GO glucose catabolic process**	21	-0.67	-1.84	0.004	0.036	0.9	1132	tags=38%, list=9%, signal=42%
**GO threonine type peptidase activity**	18	-0.68	-1.84	0	0.036	0.9	3335	tags=78%, list=28%, signal=107%
**GO bile acid metabolic process**	28	-0.61	-1.83	0.002	0.036	0.907	2202	tags=61%, list=18%, signal=74%
**GO homotypic cell-cell adhesion**	38	-0.58	-1.83	0	0.038	0.923	1197	tags=34%, list=10%, signal=38%
**GO neutral amino acid transport**	19	-0.67	-1.82	0.004	0.04	0.931	669	tags=26%, list=6%, signal=28%
**GO water soluble vitamin metabolic process**	63	-0.52	-1.82	0.003	0.041	0.937	1567	tags=40%, list=13%, signal=45%
**GO chemorepellent activity**	15	-0.7	-1.82	0.007	0.041	0.939	2603	tags=73%, list=22%, signal=93%
**GO mitochondrial matrix**	359	-0.41	-1.82	0	0.041	0.944	2493	tags=30%, list=21%, signal=36%
**GO oxidoreductase activity acting on the aldehyde OR OXO group of donors**	35	-0.57	-1.82	0.003	0.041	0.944	1767	tags=43%, list=15%, signal=50%
**GO carboxylic ester hydrolase activity**	81	-0.48	-1.81	0.002	0.042	0.948	2202	tags=40%, list=18%, signal=48%
**GO NAD metabolic process**	43	-0.56	-1.81	0	0.043	0.955	1132	tags=28%, list=9%, signal=31%
**GO 2 iron 2 sulfur cluster binding**	19	-0.65	-1.81	0.002	0.043	0.96	3029	tags=63%, list=25%, signal=84%
**GO flavin adenine dinucleotide binding**	65	-0.51	-1.81	0	0.044	0.969	1767	tags=31%, list=15%, signal=36%

ES = Enrichment Score; NES = Normalized Enrichment Score; FWER = Family-Wise Error Rate

Table S1 cont. Liver RNAseq GSEA

S1.4	GO Terms; Decreased in HLB444 relative to Control							
	Size	*ES*	*NES*	*Nom. p-val*	*FDR q-val.*	*FWER p-val.*	*Rank at Max*	*Leading Edge*
**GO phosphatidylserine metabolic process**	15	-0.71	-1.8	0	0.044	0.972	808	tags=33%, list=7%, signal=36%
**GO oxidoreductase activity acting on peroxide AS acceptor**	24	-0.62	-1.79	0.009	0.052	0.983	2441	tags=50%, list=20%, signal=63%
**GO cellular response to nutrient**	24	-0.61	-1.79	0.004	0.054	0.986	1100	tags=25%, list=9%, signal=27%
**GO positive regulation of lipid transport**	33	-0.57	-1.78	0.002	0.057	0.989	2482	tags=45%, list=21%, signal=57%
**GO dicarboxylic acid transmembrane transporter activity**	19	-0.65	-1.78	0.013	0.057	0.989	2947	tags=58%, list=25%, signal=77%
**GO oxidoreductase activity acting on paired donors with incorporation or reduction of molecular oxygen NAD P H as one donor and incorporation of one atom of oxygen**	21	-0.64	-1.78	0.004	0.057	0.989	1803	tags=52%, list=15%, signal=62%
**GO organic acid sodium symporter activity**	16	-0.67	-1.78	0.002	0.056	0.989	732	tags=44%, list=6%, signal=47%
**GO positive regulation of protein kinase B signaling**	47	-0.52	-1.78	0.002	0.056	0.989	2457	tags=43%, list=20%, signal=53%
**GO benzene containing compound metabolic process**	19	-0.65	-1.78	0.009	0.055	0.989	1483	tags=53%, list=12%, signal=60%
**GO organic anion transmembrane transporter activity**	95	-0.46	-1.77	0	0.062	0.997	1893	tags=36%, list=16%, signal=42%
**GO metal cluster binding**	57	-0.51	-1.77	0	0.061	0.997	3618	tags=53%, list=30%, signal=75%
**GO cofactor binding**	211	-0.42	-1.76	0	0.063	0.997	2496	tags=35%, list=21%, signal=43%
**GO cofactor catabolic process**	15	-0.68	-1.76	0.011	0.063	0.997	1398	tags=40%, list=12%, signal=45%
**GO glutathione metabolic process**	35	-0.55	-1.76	0	0.064	0.998	1408	tags=37%, list=12%, signal=42%
**GO organic acid transmembrane transport**	60	-0.5	-1.75	0.002	0.066	0.999	1741	tags=43%, list=14%, signal=50%
**GO aerobic respiration**	45	-0.53	-1.75	0.005	0.068	1	4632	tags=69%, list=39%, signal=112%

ES = Enrichment Score; NES = Normalized Enrichment Score; FWER = Family-Wise Error Rate

Table S1 cont. Liver RNAseq GSEA

S1.4	GO Terms; Decreased in HLB444 relative to Control							
	Size	*ES*	*NES*	*Nom. p-val*	*FDR q-val.*	*FWER p-val.*	*Rank at Max*	*Leading Edge*
**GO glucose metabolic process**	89	-0.46	-1.75	0	0.07	1	1274	tags=28%, list=11%, signal=31%
**GO symporter activity**	72	-0.48	-1.74	0.007	0.071	1	1289	tags=32%, list=11%, signal=36%
**GO glyoxylate metabolic process**	27	-0.58	-1.74	0.005	0.073	1	1408	tags=33%, list=12%, signal=38%
**GO negative regulation of actin filament depolymerization**	18	-0.64	-1.73	0.004	0.079	1	951	tags=28%, list=8%, signal=30%
**GO amino acid transmembrane transport**	34	-0.55	-1.73	0.005	0.082	1	1741	tags=44%, list=14%, signal=51%
**GO fatty acid beta oxidation**	46	-0.52	-1.72	0.008	0.086	1	2458	tags=54%, list=20%, signal=68%
**GO amino acid betaine metabolic process**	16	-0.66	-1.72	0.015	0.088	1	1295	tags=44%, list=11%, signal=49%
**GO lipid oxidation**	63	-0.48	-1.72	0.003	0.087	1	2464	tags=51%, list=20%, signal=64%
**GO sulfur compound binding**	137	-0.43	-1.72	0	0.088	1	2398	tags=36%, list=20%, signal=44%
**GO organic anion transport**	226	-0.4	-1.72	0	0.089	1	1800	tags=29%, list=15%, signal=33%
**GO secondary active transmembrane transporter activity**	112	-0.44	-1.71	0.002	0.089	1	1487	tags=29%, list=12%, signal=33%
**GO regulation of amine transport**	29	-0.57	-1.71	0	0.091	1	573	tags=24%, list=5%, signal=25%
**GO cellular biogenic amine catabolic process**	19	-0.62	-1.71	0.007	0.092	1	263	tags=32%, list=2%, signal=32%
**GO anion cation symporter activity**	27	-0.59	-1.7	0.005	0.096	1	1142	tags=41%, list=9%, signal=45%
**GO purine nucleoside biphosphate biosynthetic process**	17	-0.64	-1.7	0.009	0.096	1	829	tags=24%, list=7%, signal=25%
**GO nucleoside biphosphate biosynthetic process**	17	-0.64	-1.7	0.011	0.096	1	829	tags=24%, list=7%, signal=25%
**GO negative regulation of innate immune response**	22	-0.59	-1.7	0.005	0.098	1	2179	tags=45%, list=18%,

ES = Enrichment Score; NES = Normalized Enrichment Score; FWER = Family-Wise Error Rate
