# Supplementary material for: A mutation in mouse Krüppel-like factor 15 alters the gut microbiome and response to obesogenic diet
Source: PLoS One. 2019 Sep 25;14(9):e0222536. doi: 10.1371/journal.pone.0222536 (PMC6760833; doi:10.1371/journal.pone.0222536)
Supplement: S2 Table — (PDF) [file pone.0222536.s006.pdf]

Table S2. qPCR

S2.1	*Klf15* Expression<br>*ΔΔCT*	*Fold change*	*T-test p-value*
**Brown adipose tissue**	1.6	3.11	0.313
**Heart**	-3.4	0.09	0.026
**Kidney**	-0.7	0.62	0.004
**Liver**	1.3	2.43	0.394
**Skeletal muscle**	-1.2	0.43	0.016
**White adipose tissue**	-0.2	0.86	0.819
**S2.2**	***Pparγ* Expression**		
**Brown adipose tissue**	3.09	8.49	0.161
**Heart**	-2.21	0.22	0.0547
**Kidney**	-0.50	0.71	0.123
**Liver**	3.22	9.31	0.053
**Skeletal muscle**	1.73	3.31	0.530

ΔΔCT = detectable delta (change) in cycle threshold between comparison groups (HLB444 and Control)
