# Supplementary material for: A mutation in mouse Krüppel-like factor 15 alters the gut microbiome and response to obesogenic diet
Source: PLoS One. 2019 Sep 25;14(9):e0222536. doi: 10.1371/journal.pone.0222536 (PMC6760833; doi:10.1371/journal.pone.0222536)
Supplement: S3 Table — (PDF) [file pone.0222536.s007.pdf]

Table S3. Descriptive Statistics for 16S Data

	B6					HLB				
	T0	*T1**	*T2**	*T3**	TT	T0	*T1**	*T2**	*T3**	TT
**N**	**7**	*6*	*7*	*7*	**7**	**11**	*10*	*8*	*11*	**10**
**Mean read depth/sample ± SD**	**17463 ± 3117**	*18432 ± 1457*	*21592 ± 6929*	*20789 ± 2020*	**19336 ± 2335**	**19428 ± 3082**	*17966 ± 1747*	*19671 ± 1894*	*19464 ± 2250*	**17704 ± 2546**
**Mean # OTUs/sample ± SD**	**166 ± 16**	*187 ± 7*	*156 ± 10*	*146 ± 11*	**129 ± 13**	**153 ± 30**	*201 ± 12*	*189 ± 5*	*178 ± 9*	**154 ± 11**

**not used in most analyses.* Sample count for each genotype by time point along with mean read depth per sample and mean number of OTUs per sample ± standard deviation. There were not large community differences between T1 and T3, thus all analyses were performed on T0 and TT.
