# Supplementary material for: A mutation in mouse Krüppel-like factor 15 alters the gut microbiome and response to obesogenic diet
Source: PLoS One. 2019 Sep 25;14(9):e0222536. doi: 10.1371/journal.pone.0222536 (PMC6760833; doi:10.1371/journal.pone.0222536)
Supplement: S4 Table — (PDF) [file pone.0222536.s008.pdf]

Table S4. Inferential Statistics for 16S Data

	Main & Interaction Effects
**S4.1**	***Shannon Diversity***
**Genotype**	F(1,12) = 9.942, p = .008
**Diet (Time)**	F(4,48) = 2.385, p = .064
**Genotype * Diet**	F(4,48) = 5.133, p = .002
**S4.2**	***Observed Diversity***
**Genotype**	F(1,12) = 20.077, p < .001
**Diet (Time)**	F(4,48) = 25.795, p < .001
**Genotype * Diet**	F(4,48) = 8.510, p < .001
**S4.3**	***Read Depth, Shannon Diversity***
**B6**	r^2 = .013, t(32) = -0.657, p = 0.517
**HLB444**	r^2 = .036, t(48) = -1.345, p = 0.185
**S4.4**	***Read Depth, Observed Diversity***
**B6**	r^2 = .005, t(32) = 0.401, p = 0.691
**HLB444**	r^2 = .003, t(48) = 0.385, p = .702

S4.5 ***PERMANOVA***

	T0	*T1**	*T2**	*T3**	*TT*
**Genotype**	F(1,14) = 22.246, p < .001	F(1,12) = 10.478, p < .001	F(1,11) = 13.810, p < .001	F(1,14) = 11.429, p < .001	F(1,13) = 16.421, p < .001
**Sex**	F(1,14) = 2.724, p = 0.054	F(1,12) = 2.402, p = .056	F(1,11) = 1.148, p = .288	F(1,14) = 0.979, p = .393	F(1,13) = 1.620, p = 0.177
**Genotype * Sex**	F(1,14) = 1.383, p = 0.212	F(1,12) = 2.301, p = .065	F(1,11) = 1.366, p = .220	F(1,14) = 1.636, p = .137	F(1,13) = 1.941, p = 0.116

**not used in most analyses.* Parametric and non-parametric statistics for gut microbiome (Figure 6).
