# Supplementary material for: A mutation in mouse Krüppel-like factor 15 alters the gut microbiome and response to obesogenic diet
Source: PLoS One. 2019 Sep 25;14(9):e0222536. doi: 10.1371/journal.pone.0222536 (PMC6760833; doi:10.1371/journal.pone.0222536)
Supplement: S1 File — (PDF) [file pone.0222536.s009.pdf]

Supplemental Methods

Primers used for amplification of *Klf15* mutation: PCR was performed at 60°C annealing temperature. A 331-bp amplicon containing the T->A variant was generated using the following primers: Forward: 5'- CCACACCTGGCTGCACCTAATTCTT-3'; Reverse: 5'- GGCTGCACGGTAAGCAGGATTACAA-3'.

Tissue expression: RT-PCR was performed on cDNA that was reverse-transcribed from liver, heart, lung, muscle and fat RNA from both control and HLB444 animals with the exon spanning primers Primers used for PPAR-gamma were forward: 5'- ATATGACCTGAAGCTCCAAG- 3' and reverse: 5'- CAGGTTCTACTTTGATCGCA-3'. Primers used for Klf15 were forward: 5'- TACACCAAGAGCAGCCACCT-3' and reverse: 5'-AACTCATCTGAGCGGGAAAA-3'. Primers used for Gapdh were forward: 5'-GTGCCAGCCTCGTCCCGT-3' and reverse: 5'- CTGTGCCGTTGAATTTGCCGT."

Metabolic cages: After gas calibration and calibration of Mass Modules to measure food update and water consumption for each cage, mice were housed in Promethion Systems metabolic cages (Sable Systems International, Las Vegas, NV) for a period of three days- two nights. Activity was monitored via pedometer, O_2 and CO_2 levels were continuously sampled. Data was collected using MetaScreen software (v. 2.2.19). Data was analyzed using ExpeData software (v. 1.8.4). Outputs included Respiratory Quotient and associated Metabolic Rate, Relative Humidity, kcal/hr, food uptake and water consumption.

Electrocardiogram assessment: Electrocardiogram traces were obtained from unanesthetized mice using the ECGenie™ system (Mouse Specifics, Inc., Quincy, MA). The system features a

platform standing 18” above the laboratory bench containinga plate with three electrodes upon which mice are placed and allowed to acclimate for 5 minutes. The trace is initiated when mice contact the electrodes with three paws. Traces are obtained over a 30-60 second period. Fast Fourier analysis detects P, Q, R, S, and T waves for evaluation of heart rate, heart rate variability, and PR, QT, QRS complex, and ST interval durations. The QT interval duration is corrected for heart rate, producing the QTC value.

Blood pressure measurements: Systolic and diastolic blood pressure and pulse were measured using a tail-cuff method (Visitech Systems, Apex, NC). Mice were trained to the apparatus for 2 days before measurements were recorded over the following 2 days. Detection equipment includes a warmed platform on which unanesthetized mice are held in place with a magnetic restraining box that is open at the nose end, and the tail is guided through the cuff. A computer drives the inflation and deflation of the tail cuff, as a sensor positioned at the base of the tail detects when blood flow starts and stops. Training and recording are performed in the morning over a period of 20 min on a platform that accommodates six mice. Each test day comprises 30 measurements. Hence, at the end of the test, average parameter values obtained per mouse are based on 60 measurements.
